# The T Cell Epitope Landscape of SARS-CoV-2 Variants of Concern

**DOI:** 10.3390/vaccines10071123

**Published:** 2022-07-14

**Authors:** Simen Tennøe, Marius Gheorghe, Richard Stratford, Trevor Clancy

**Affiliations:** NEC OncoImmunity AS, Oslo Cancer Cluster, Ullernchausseen 64/66, 0379 Oslo, Norway; simen@oncoimmunity.com (S.T.); marius.gheorghe@oncoimmunity.com (M.G.); richard@oncoimmunity.com (R.S.)

**Keywords:** SARS-CoV-2, COVID-19, immunogenicity, variants of concern, Alpha, Beta, Gamma, Delta, Omicron

## Abstract

During the COVID-19 pandemic, several SARS-CoV-2 variants of concern (VOC) emerged, bringing with them varying degrees of health and socioeconomic burdens. In particular, the Omicron VOC displayed distinct features of increased transmissibility accompanied by antigenic drift in the spike protein that partially circumvented the ability of pre-existing antibody responses in the global population to neutralize the virus. However, T cell immunity has remained robust throughout all the different VOC transmission waves and has emerged as a critically important correlate of protection against SARS-CoV-2 and its VOCs, in both vaccinated and infected individuals. Therefore, as SARS-CoV-2 VOCs continue to evolve, it is crucial that we characterize the correlates of protection and the potential for immune escape for both B cell and T cell human immunity in the population. Generating the insights necessary to understand T cell immunity, experimentally, for the global human population is at present a critical but a time consuming, expensive, and laborious process. Further, it is not feasible to generate global or universal insights into T cell immunity in an actionable time frame for potential future emerging VOCs. However, using computational means we can expedite and provide early insights into the correlates of T cell protection. In this study, we generated and revealed insights on the T cell epitope landscape for the five main SARS-CoV-2 VOCs observed to date. We demonstrated using a unique AI prediction platform, a significant conservation of presentable T cell epitopes across all mutated peptides for each VOC. This was modeled using the most frequent HLA alleles in the human population and covers the most common HLA haplotypes in the human population. The AI resource generated through this computational study and associated insights may guide the development of T cell vaccines and diagnostics that are even more robust against current and future VOCs, and their emerging subvariants.

## 1. Introduction

During the COVID-19 pandemic [1], the international community experienced several SARS-CoV-2 variants that caused various degrees of altered infectivity, potential immune escape, or both, compared to the original wildtype Wuhan strain. Some of these variants had characteristic mutations that changed the epidemiology of the pandemic, and indeed some of these variants altered the clinical impact of COVID-19. There were five main lineages that were designated as SARS-CoV-2, so called, “variants of concern” (VOC). These lineages systematically ushered in new waves of infection, changing the nature of the pandemic, and were accompanied by different health and socioeconomic challenges. These five VOCs (Alpha, Beta, Gamma, Delta, and Omicron) shared several mutations, as well as harboring several VOC-specific variants. The most recent VOC that emerged, the hyper-transmissible Omicron, was a presage to us all that VOCs will continue to emerge and pose a continuous health and socioeconomic threat, particularly now, as the SARS-CoV-2 virus now becomes endemic in the human population [2].

The VOCs were primarily characterized based on their increased infectivity through specific mutations within the receptor binding domain (RBD) of the spike protein with key properties that often rendered the mutated RBDs with either (1) increased potential to enter host cells through an increased binding affinity for its cognate receptor, or (2) evasion of neutralizing antibody protection through a diminished capacity of host antibodies to bind to the mutated RBD and inhibit its ability to bind to the host receptor [3]. A more deadly variant that confers both an increased infectivity and an evasion of vaccine- or infection-induced immune protection will create very difficult challenges [2,4]. Therefore, developing broadly protective vaccines with a focus on emerging virulent VOCs will be important to ensure the human population maintains protective immunity against future emerging SARS-CoV-2 VOCs [5].

In the quest to develop broadly protective vaccines against emerging VOCs, researchers have increasingly looked beyond the traditional antibody-centric approaches and begun to focus on characterizing the requisite T cell responses that correlate with robust and lasting protection against COVID-19 [6]. Although neutralizing antibody protection from infection or vaccination is the current gold standard, there was widespread escape from pre-existing vaccine-induced, or naturally acquired, neutralizing antibody responses during the Omicron wave [7]. Interestingly, many infected SARS-CoV-2 individuals during the COVID-19 pandemic demonstrated virus-specific T cell responses in the absence of measurable virus-specific antibodies [8,9]. Numerous studies during the pandemic demonstrated that T cell immunity played a critical role in protection against the virus [10,11]. For example, a systems immunology approach that analyzed high dimensional molecular data from 139 COVID-19 patients representing various degrees of disease severity, revealed that CD8^+^ T cells were associated with an improved clinical outcome [12]. Additionally, common T cell epitopes were identified between SARS-CoV-2 and human seasonal coronaviruses (HCoV) and T cell responses against these cross-reactive epitopes were important for the observed clinical protection against COVID-19 [13,14,15,16]. Although individual viral mutations do have the potential to diminish or destroy effector T cell responses at the level of the individual by destroying important HLA-restricted epitopes, it is highly unlikely that these same mutations will confer a selective advantage (via immune escape) in other individuals with different HLA haplotypes, or at the level of a population, which has a highly diverse HLA haplotype landscape. This rational is further strengthened when we consider the size of the SARS-CoV-2 RNA genome, which is approximately 30 kb encoding 14 open reading frames (some of which are overlapping) and offers ample protein “real estate” for HLA-restricted T cell immunity. Consequently, T-cells can exploit the whole viral proteome, and are not limited to the real estate offered by the spike protein, particularly the RBD domain, unlike the humoral response. Furthermore, additional T cell protection can be acquired from cross-reactive T-cells stimulated in populations previously exposed to other members of the betacoronavirus family. For example, T cell immunity reported during the Middle Eastern respiratory virus (MERS) outbreak was demonstrated to be more robust compared to the B cell antibody response [17].

These findings and observations strongly suggest that T cell immunity is a key correlate of protection against beta-coronavirus infections including SARS-CoV-2 and its VOCs, and that adopting vaccine strategies that can drive broader and more potent T-cell responses will be the key to developing more efficacious and broadly protective vaccines in the future. Whilst neutralizing antibody responses are essential to prevent transmission and provide sterile immunity, they are short-lived, as has been observed for SARS-CoV-1, SARS-CoV-2, and the seasonal coronaviruses [18]. For example, while antibody responses were shown to be short-lived in patients infected with SARS-CoV-1 [19,20], the corresponding T cell responses in the same patients were detected 17 years after infection [19]. Interestingly, the current mRNA-based spike-centric vaccines against SARS-CoV-2 have been shown to induce broad T cell responses that recognize several SARS-CoV-2 variants and human seasonal coronaviruses [21] in addition to driving neutralizing antibody responses. T cell responses were measured and detected in most vaccine studies during the COVID-19 pandemic [22]. In fact, protective clinical benefits of vaccination were seen as early as 11 days after the first vaccination, coinciding with a robust T cell response [23]. Even before the emergence of the most recent VOC, Omicron, T cell responses induced by vaccines demonstrated strong cross-protection against different VOC [24].

In terms of the most recent hyper-transmissible Omicron VOC, its increased infectivity cannot be explained by a higher viral load alone [25,26], pointing to immune evasion as an explanation [27]. Further, it was demonstrated very early in Omicron’s rapid spread that it evades antibody neutralization from both vaccinated and convalescent individuals [28,29,30,31,32]. In addition, the large number of new mutations in the Omicron VOC (32 mutations in the spike protein alone) also raised fears that these changes might enable the virus to circumvent pre-existing T-cell immunity induced by the vaccine or by natural infection with earlier VOCs. However, despite Omicron’s extensive number of mutations and its ability to escape from neutralizing antibodies in vaccinated and convalescent individuals, T cell responses induced by both vaccination and infection remained robust and were able to eliminate Omicron-infected cells [33,34]. This observation led to the speculation that well-preserved T cell immunity to Omicron contributes to protection from severe COVID-19 disease [34,35]. Interestingly, T cell immunity induced by SARS-CoV-2 vaccines was demonstrated to be highly cross-reactive against the Omicron and Delta variant [36], which suggests that the current vaccines may actually be providing some protection against severe disease via the cellular arm of the immune system, by stimulating T-cell responses against the HLA restricted T cell epitopes on the spike protein.

However, if the current vaccines do indeed mediate some of their protection by stimulating the cellular arm of the immune system, such protection is clearly limited to the HLA-restricted epitopes that exist in the spike protein and it would be clearly beneficial to consider additional epitope-rich regions that exist across the entire proteome of the virus. This would be critically important if we envisage designing future T-cell-centric vaccines that can provide universal protection across the global spectrum of HLA haplotypes in the human population. Consequently, a better mapping of the T-cell epitope landscape across multiple different HLA’s will empower the vaccine community with the necessary knowledge and cellular immunity toolkit to guide future vaccine design and development and facilitate the development of vaccines that are better equipped to combat future VOCs.

In this study, we have mapped the predicted T cell epitope landscape for the five main SARS-CoV-2 VOCs. Through the exploitation of state-of-the-art in silico methods that incorporate advanced artificial intelligence predictors of T cell immunogenicity, we profiled the Class I HLA-restricted epitope landscape for the five VOCs against 156 of the most frequent HLA alleles in the human population. We demonstrate conclusively in this extensive analysis that immunogenic T cell epitopes are conserved across all VOCs, for almost all HLA alleles in the human population. The experimental accumulation of this evidence using in vitro or in vivo approaches would require extensive, laborious, expensive, and time-consuming efforts. The far more comprehensive in silico analysis described in this study and the subsequent community resource it generated corroborates the existing recent experimental evidence of robust T cell protection against VOCs (whereby the experimental evidence is supported by only a very limited number of HLA alleles). Taken together, the resource generated through this computational study may guide the development of future T cell vaccines and diagnostics that are more robust against current and future VOCs and their subvariants. In addition, the statistical and AI predictive metrics applied here may be deployed to quickly gauge the potential alteration of cellular immunity against future SARS-CoV-2 lineages that may characterize emerging VOCs, as we show for the Omicron-BA2 strain.

## 2. Results

### 2.1. A Resource of Mutated Epitopes in VOCs from the Perspective of Their Antigen Presentation Potential

We performed a pairwise comparative analysis of the predicted antigen presentation (AP) scores between each of the VOCs and the original SARS-CoV-2 strain identified in Wuhan. The AP scores were computed using a state-of-the-art AI engine that predicts the potential of HLA-epitope complexes to be presented on the surface of host infected cells by Class I HLA alleles, and therefore recognizable by cognate T cells (see Methods). The peptides originating from the Wuhan strain are referred to here as the “wildtype”, while the mutated peptides emerging from any of the VOC are referred to as “mutant”. In this study, we limited the scope of our analysis to an examination of the differences in AP potential among either the mutated or wildtype peptides that were predicted to have a high likelihood of being bound to HLA and presented to the cell surface. Therefore, in the following analyses, the AP scores were filtered such that only peptides with an AP score >0.5 (in a scoring system that ranges from 0 to 1) in either the mutant or the corresponding wildtype peptide were considered. Consequently, the analyses presented here only considers potential T cell epitopes that either have a high AP score in both the wildtype and the associated mutant peptide or were presented with a gain or loss of AP potential as an outcome of the mutation. The selection of 0.5 as an AP score threshold was motivated by (i) the need for a sufficiently high AP potential, such that we ignore peptides with a negligible or low chance of being presented on the cell surface, and (ii) the need to have enough viable epitopes for the comparison of AP score distributions between the mutant and the wildtype (i.e., to ensure statistical power). However, the exact choice of threshold is not critical since we only compare differences between variants. The threshold can be fine-tuned in future analyses, and we provide the entire landscape of AP predictions for the mutated peptides and their corresponding wildtypes across all VOCs (i.e., irrespective of their AP score) across 156 of the most frequent Class I HLA alleles in the human population in Supplementary File S1, as a resource to the community to further explore the data.

### 2.2. The AP Profile (A Proxy of T-Cell Immunity) Is Robust across All SARS-CoV-2 VOCs

**Figure 1:**
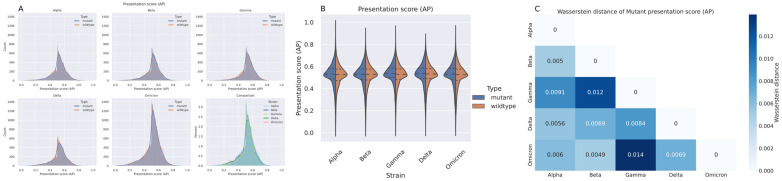
Comparisons of the distribution of antigen presentation (AP) scores for each SARS-COV-2 variant of concern (VOC) for the mutated and corresponding wildtype peptides. The AP scores have been filtered such that only peptides with AP scores >0.5 in either the mutant or corresponding wildtype are shown. (**A**) Histogram with a kernel density estimate of the distribution of AP scores for the mutant and wildtype peptides for each VOC. The subfigure “Comparison” shows the percentage of AP scores in each bin for all VOCs. (**B**) Violin plots of the AP score distribution between mutant and wildtype peptides for each VOC. The dashed lines within each violin plot represent quartiles. (**C**) The pairwise Wasserstein distance between the distribution of AP scores for the mutated peptides in the VOCs.

Non-synonymous mutations in the proteins of SARS-CoV-2 had almost no effect on their potential to be presented by the most frequent HLA alleles in the human population and are thereby recognized by cognate HLA-restricted CD8^+^ T cells from a global perspective (the 156 most frequent Class I HLA alleles across all mutated peptides in all VOCs). We observed highly similar AP score distributions between the mutant and corresponding wildtype peptides. It should be noted that we were not, in this analysis, interested in the difference in AP scores between the mutant and wildtype at the individual peptide level, as the loss in AP score in one peptide might be offset by a gain in AP score in another peptide. Instead, we focus on the distribution of the AP scores that are illustrated in Figure 1A for each VOC. The variation in peptide counts we observed was due to a different number of mutated peptides for each VOC that passed the 0.5 AP threshold. The number of mutated peptides was simply a function of the number of mutations for a given strain (i.e., more mutations translated to more mutated peptides). When investigating these distributions pooled across all VOCs, we observed that the shapes of these distributions were very similar, as depicted in Figure 1A, subfigure titled “Comparison”. Here, the AP score distributions were layered on top of each other, using percentages for each bin instead of the raw count. The reason why no sharp cutoff can be observed around an AP of 0.5 is because a peptide was considered for analysis if it has an AP score >0.5 in either the mutant or wildtype. Figure 1B shows the same AP score distributions with a violin plot representation to further facilitate the interpretation of these pairwise comparisons. Again, we observed that the difference in AP score distributions between the mutant and wildtype was negligible.

The same trend was observed when we analyzed the relatively minor shifts in AP score distributions between VOCs. This is confirmed by Figure 1C where the Wasserstein distance [37], also commonly called “the earth mover” distance, was calculated and plotted for the pairwise distance between each VOC. The intuitive explanation for the Wasserstein distance is how much “work” it takes to transform one distribution into another. In other words, it reflects how much of one distribution we need to move, multiplied by the distance it is moved. Note that the unit of the Wasserstein distance is the same as for the AP score. An advantage of investigating the Wasserstein distance between these distributions is that it takes the specific shape of each distribution into account. A small Wasserstein distance, in our case close to zero, therefore corresponds to the two distributions being very similar, while a large Wasserstein distance means the two distributions are very different. The Wasserstein distance has no upper boundary as the distributions can be arbitrarily far away from each other, but for this work a distance closer to one would be considered large. As Figure 1C shows, only small Wasserstein distances were observed between the different VOCs.

Table 1 summarizes the differences in AP score distributions between mutant and wildtype peptides within each VOC. The average difference in AP scores, column one in Table 1, is an intuitive metric to assess the impact of the collective set of mutations occurring within a VOC. It can quickly show us in a global manner if there was a general gain or drop in the AP scores in a VOC compared to the wildtype strain. However, a drawback of the mean difference, is that it only considers the mean of the distributions and does not capture the shape of the AP score distributions.

The two-sided Kolmogorov–Smirnov (KS) test statistic, the second column in Table 1, shows the largest absolute difference between the two cumulative empirical distribution functions and lies in the interval (0, 1). Like the Wasserstein distance, the two-sided KS test statistic takes the location and shape of the two distributions into account and gives a measure of the distance between the two. However, the maximum distance is reached as soon as the two distributions no longer overlap. This is a crucial difference with respect to the Wasserstein distance, which continues to increase the further the two distributions diverge from each other. It should be noted that while the *p*-value for the KS test is significant (<0.05) (i.e., the AP scores come from statistically different distributions) for all but the Beta variant, the *p*-value does not inform us on how different the distributions are from each other.

Overall, Table 1 shows us that the different metrics are in good agreement when considering the difference in AP score distributions between the mutant and wildtype, for each VOC. All three metrics are very low, considering that the range of AP scores and KS statistic is (0, 1). Moreover, ranking the VOC from the largest to the smallest difference between the mutant and wildtype based on the KS statistic or Wasserstein distance results in the same order.

However, this analysis compared only the subset of peptides containing mutations to their corresponding wildtype counterpart. As Table 2 shows, this subset of mutated peptides represents only a small fraction out of the total number of peptides in the original SARS-CoV-2 Wuhan strain. In this table, Wildtype refers to the number of peptides with an AP score >0.5 in the complete, original Wuhan strain. As column three shows, the number of peptides with an AP score >0.5 in either the mutant or its corresponding wildtype counterpart, for each of the VOCs, is relatively small when compared to the number of wildtype peptides. Even for Omicron, the VOC with the highest number of mutations (59), we observe that only ~5% of the peptides with a high AP score are mutated when compared to the Wuhan wildtype. In other words, Table 2 helps to put the results presented here in perspective, as they only reflect the small fraction of peptides that underwent nonsynonymous mutation events. Therefore, with the comparison of the AP score distributions in mind, one must consider the fact that ~95% or more of the peptides with an AP >0.5 in each VOC are of course identical.

As previously mentioned, the Supplementary File S2 (S2-Figures S2, S4, S6, and S10 and the raw data table) also offers, as a resource, the complete set of predicted AP scores for all mutated peptides and all HLA alleles considered for each of the VOCs without any filtering, allowing investigators to study the distributions of the entire set of AP scores.

We also examined the recently emerged more transmissible Omicron sub-lineage BA.2 as a use case, the plots including this variant can be found in Supplementary File S2 (S2-Figures S1, S3, S5, S7, and S9). In brief, Omicron BA.2 diverged slightly further from the wildtype than Omicron and presents an increase in the average difference of the AP score when compared to Omicron, suggesting that it may be more immunogenic from the perspective of T-cell immunity (predicted epitope density). However, the differences remain small. Overall, the biggest difference in AP scores due to mutations was found in Gamma (Wasserstein: 0.0134, avg. difference in AP score: 0.0069) and Omicron-BA.2 (Wasserstein: 0.0108, avg. difference in AP score: 0.0108).

### 2.3. The Antigen Presentation Potential across Different VOCs Does Not Significantly Differ for Most of the HLA Alleles Considered in the Analysis

We next examined the distribution of AP scores for the mutant and the corresponding wildtype peptides on an HLA allele specific basis, to assess if there are specific HLA alleles in the human population that are more susceptible to immune escape by the emerging VOCs.

Table 3 shows the mean and the 5th percentile (for the average AP score difference only), in addition to the 25th, 75th, and 95th percentiles of the Wasserstein distance, two-sided KS test statistic, and of the average AP score difference between the mutant and wildtype epitopes for each VOC. The mean and percentiles were calculated based on the set of individual values for each metric that were inferred for each of the available HLA alleles per VOC. Since the Wasserstein distance and the two-sided KS test statistic have a minimum value of 0, we are only concerned with the larger percentiles. For the average AP score difference on the other hand, which can range from −1 to 1, we looked at the mean, 25th, and 75th percentiles, in addition to the 5th and 95th percentiles to capture the outliers.

From Table 3, we observed that the mean of the average difference in AP score and the mean of the Wasserstein distance are both very small. The mean of the two-sided KS test statistic is slightly larger, as expected, but can still be considered small in this context. This means that on average we see no difference between the AP score distributions for the mutant and wildtype peptides for the three metrics of interest. The absolute values of the 25th and 75th percentiles of the average difference in AP scores were also small, therefore we examined the 5th and 95th percentiles to see the larger differences. However, no extreme shifts in the average difference in AP scores were observed. For the Wasserstein distance, we observed a similar distribution at the 75th percentile, whereby the differences in distributions between the mutant and wildtype were relatively small, while a marginally larger difference was first observed only at the 95th percentile. As for the mean, this implies that there is little difference between the AP score distributions for mutant and wildtype peptides for the three metrics of interest for ~75% of the examined alleles. It is only for ~5% of the alleles that we see larger differences.

**Figure 2:**
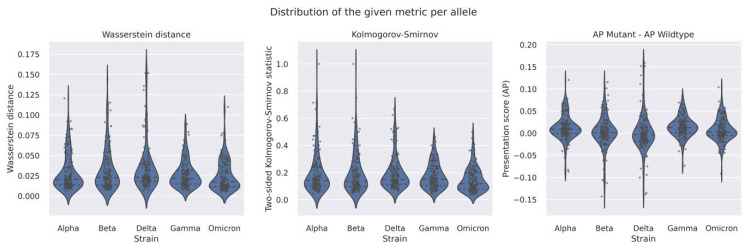
Violin plots of the distributions of the Wasserstein distance, two-sided Kolmogorov–Smirnov statistic, and average difference in AP score between mutant and wildtype epitopes across the HLA alleles, for each VOC. The dashed lines within each violin represent the quartiles and the dots show the score for each metric for each epitope.

In Figure 2 we plotted the distributions of these three metrics across the entire set of HLA alleles for each VOC. We observed that out of the 156 HLA alleles, the vast majority do not show substantial differences between the two AP score distributions, as also shown in Table 3. This suggests that, at a first glance, there is no apparent HLA (sub)population at more risk than other HLA (sub)populations due to lower predicted epitope densities (and presumably corresponding T-cell responses) to a given VOC versus the WT. Plots of the AP score distribution for each allele, can be found in Supplementary File S2 (S2-Figure S5).

**Figure 3:**
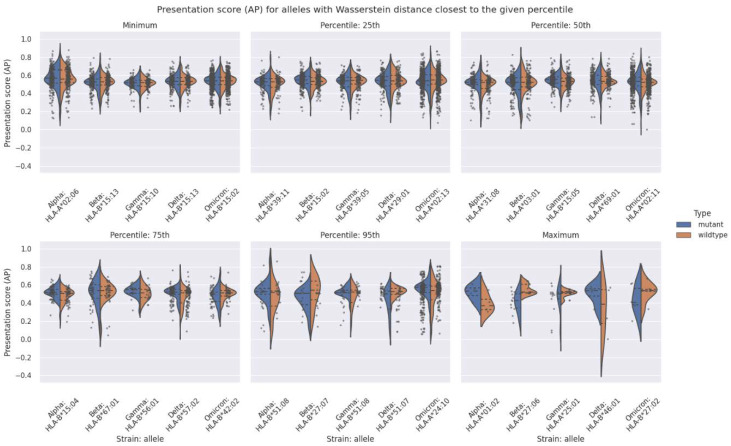
Violin plots of the AP score distribution for the mutant and wildtype peptides for the HLA alleles with a Wasserstein distance closest to the minimum, maximum, or given percentile of all the Wasserstein distances for that VOC. The dashed lines within each violin represent the quartiles and the dots show the AP for each peptide.

Figure 3 shows the distribution of AP scores for mutant and wildtype epitopes for a selected group of HLA alleles. Each plot in the figure corresponds to either the minimum, maximum, or a specific percentile. For each VOC, we selected HLA alleles such that their Wasserstein distance, between mutant and wildtype is the closest to the minimum, maximum, or the given percentile. For instance, for “Percentile: 25th”, the HLA alleles shown are the ones for which the Wasserstein distance is the closest to where 25% of the data lives with respect to the Wasserstein distance metric. This analysis serves as a guide to establish a connection between the AP score distributions and the Wasserstein distance by showing representative samples for the AP score distributions between mutant and wildtype epitopes. A more detailed figure with all HLA alleles can be found in Supplementary File S2 (S2-Figure S3).

From Figure 3 we observed that up to and including the 50th percentile. (i.e., the median) the differences in the AP score distribution between mutant and wildtype are relatively small. Even at the 75th percentile (i.e., the third quartile) the AP score distributions are very similar.

As such, the consensus conclusion from Table 2 and Figure 2 and Figure 3 is that only ~5% of the 156 HLA alleles analyzed demonstrated larger differences in AP score distributions between the mutant and wildtype. Consequently, most HLA alleles in the human population do not demonstrate an altered propensity to present mutated SARS-CoV-2 peptides across the different VOCs.

Even if the vast majority of HLAs present a similar AP score distribution between the mutant and wildtype, as Figure 3 shows, a very small subset of HLA alleles presented a considerable gain or loss in AP score between the mutant and wildtype (see Figure 3, “Percentile: 95th or “Maximum”). These correspond to the HLA alleles with the largest Wasserstein distance between the mutant and wildtype, for each VOC. Based on these findings, we then assessed which haplotypes are potentially the most affected. We took the three HLA alleles per VOC that showed the greatest Wasserstein distance in AP score distributions between the mutant and wildtype and queried them against the Allele Frequency Net Database (AFND) [38]. This analysis did not highlight any specific ethnicity or region of the world to be systematically affected by a given VOC, although the Australian population seems more affected by three of the VOCs as Table 4 shows.

Another interesting observation, summarized in Table 5, was that the HLA-A alleles often had a Wasserstein distance that was an order of magnitude higher than the HLA-B alleles. Additionally, the HLA-A alleles have a higher average AP score difference than the HLA-B alleles, for all VOCs but Beta.

### 2.4. A Mutation-Centric Perspective of the T Cell Epitope Landscape of VOCs

**Figure 4:**
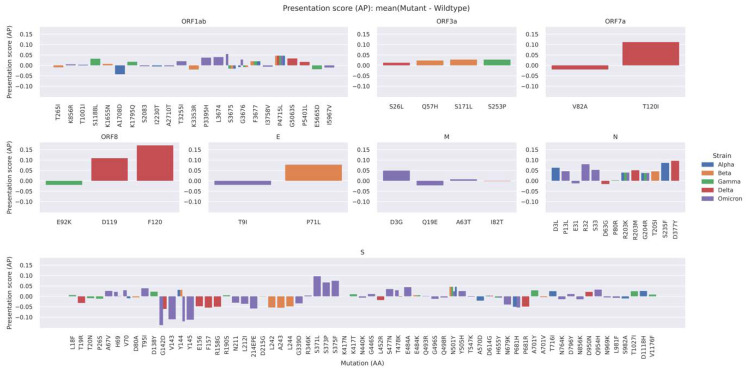
Average difference in AP score (mutant–wildtype) for each mutation in the VOC, denoted by the different colors. Each plot corresponds to a protein of the virus in which mutations occurred.

To assess the effect of a specific non-synonymous mutation on the potential of the subsequent mutated peptides to be presented as an HLA-bound epitope to T-cells, we examined the average difference in AP score between mutant and wildtype epitopes for each non-synonymous mutation in each VOC. The results are illustrated in Figure 4, stratified by each of the different SARS-CoV-2 proteins. We chose the average difference in AP score since we were specifically interested in the direction of the change around each mutated peptide. A positive value means that the AP score on average is increased for a given mutation, while a negative score means that the AP score decreases for that mutation in the variant. From Figure 4, we see that while most mutations do not seem to have a substantial impact with respect to the AP score, there are a few outliers.

**Figure 5:**
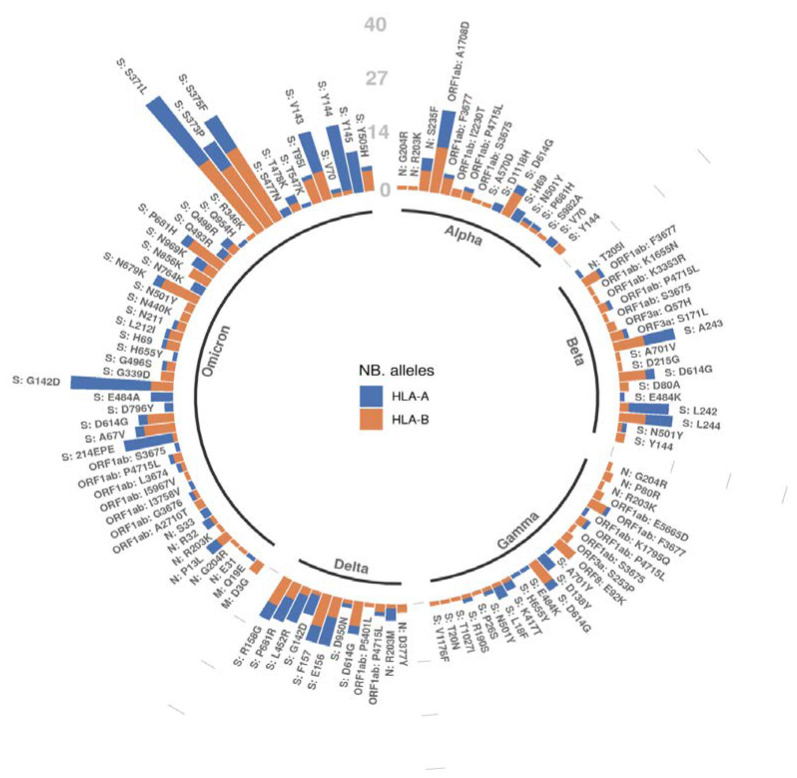
The number of HLA alleles per mutation for which there was a significant difference (*p*-value < 0.05) using Table 2. For each mutation, all overlapping peptides of length 9 and 10 that overlap a given mutation were considered as candidate HLA-restricted epitopes.

Distinct sets of mutations that were found to have the largest impact in AP scores, were generally co-occurring on the same candidate epitope. As depicted in Figure 5, co-occurring mutations, for example S: S371P, S: S373F, and S: K375N in the Omicron VOC, were more likely to increase the difference in AP potential of the candidate epitopes where they lie. In turn, this makes these specific amino acid variations more impactful in terms of resulting T cell immunity. In this specific case, there was a gain in the AP scores for all three of the co-occurring mutations.

Figure 5 summarizes the number of HLA alleles per mutation that present a significantly different AP score (*p*-value <0.05 for the two-sided KS test) as compared to the corresponding wildtype peptides, we also provide in Supplementary File S2 similar plots with a summary of the number of peptides with an AP score >0.5 in either the mutant or wildtype, which served as an input for the AP score difference significance test (Supplementary File S2, S2-Figure S9). Notably, as also observed in Table 5, there seems to be a general trend for HLA-B alleles to be more affected than HLA-A alleles with respect to the AP score.

## 3. Methods

### 3.1. Shared Mutation Profile of VOCs

The list of mutations for each VOCs, as labeled by the World Health Organization (WHO), was compiled from https://www.who.int/en/activities/tracking-SARS-CoV-2-variants (accessed on 22 March 2022). For each mutation, all overlapping peptides of length 9 and 10 that overlap a given mutation were considered as candidate HLA restricted epitopes.

### 3.2. Predicted Probability of SARS-CoV-2 Mutated Peptides Being HLA-Presented on the Surface of Host Infected Cells

The important determinants of antigen presentation (AP) were assessed for each mutated peptide and their wildtype counterpart in the VOCs for their potential to be efficiently presented. These determinants consisted of: (1) the predicted binding affinity between the candidate peptide and 156 of the most frequent HLA molecules in the human population, (2) the predicted potential of the candidate peptide to be efficiently processed by the antigen processing machinery of the host infected cell and (3) the predicted probability of the candidate mutated peptide to be presented on the host infected cell surface, which, among other factors, takes binding and processing into account. The AI prediction platform used was the NEC Immune Profiler (NIP), which provided the AI predictions for these key determinants, such as the AP scores [39]. The AP score is in the range between 0 and 1, with 1 being the maximum of the likelihood that a specific candidate mutated peptide was presented on the host infected cell surface.

The AP scores were calculated based on a set of 156 most frequent Class I HLA alleles (A and B) for all the possible combinations between an HLA allele and each epitope overlapping a given mutation, for each VOC. The complete set of raw AP score predictions is provided in the raw data table in the Supplementary File S2.

### 3.3. Statistical Analysis

The statistical analyses were performed in the Python programming language (version 3.6.12) using the *SciPy* (version 1.3.1) package, which implements functions for the Kolmogorov–Smirnov test and Wasserstein distance. For validation and complementarity purposes, the R programming language (version 4.1.1) was also used to generate a separate set of statistics on the same input data. Package *stats* in base R was used for the Kolmogorov–Smirnov test and package *transport* (version 0.12-2) to calculate the Wasserstein distance.

## 4. Discussion

The health and socio-economic burden of current and future potential emerging SARS-CoV-2 VOCs, remains entirely unknown. The most recent Omicron VOC that emerged, fortunately produced a milder COVID-19 disease, especially in highly vaccinated or infected populations [7]. The milder manifestation of the disease was in the backdrop of far higher transmissibility of Omicron compared to Delta, and indeed all other VOCs. However, Omicron and the recently emerged Omircron-BA2 subvariant, continues to circulate with hyper-transmissible rates [27] accompanied by high immune escape, as measured through antibody serological responses [40].

The continuous rapid circulation of Omicron emphasizes the importance of being able to predict the various clinical, epidemiological, and immune-escape parameters of VOCs in the human population, as Omicron and its subvariants transition toward an endemic threat [41]. However, as SARS-CoV-2 progresses on its path toward becoming endemic [42], this transition may not necessarily equate to a less virulent or milder disease [4]. There is strong reasoning that Omicron’s manifestation as a milder COVID-19 disease, in the backdrop of its rapid antigenic evolution, may be a coincidence, and more virulent and dangerous immune escape variants may emerge in the future [43]. The distinct antibody-based immune escape observed during the COVID-19 pandemic was not only limited to Omicron [3], as several other VOCs also demonstrated a reduced susceptibility to being neutralized by vaccine-induced antibodies [44]. Accordingly, as the human population acquires increasing levels of immunity through vaccination and/or infection, the evolutionary trajectory of SARS-CoV-2 toward increasing infectivity (through optimized host receptor binding), alone, will not satisfy its natural evolutionary drive toward increasing transmission rates. To combat the increasing immunity in the human population, SARS-CoV-2 is predicted to also evolve by escaping natural- or vaccine-induced immunity and gain the ability to infect previously protected individuals [43]. Thus, as SARS-CoV-2’s future evolution drives ever increasing levels of antigenic drift, it is important that we characterize the correlates of protection and potential immune escape for both cellular and humoral immunity, to guide future vaccine and diagnostic design.

As discussed in the introduction to this study, the T cell immunity that correlates with protection against SARS-CoV-2 has been shown to be more durable and cross protective [45] compared to the relatively transient and narrow (strain specific) protection afforded by antibody responses, as witnessed by the recent Omicron VOC [21]. Although there have been some efforts to profile T cell responses against SARS-CoV-2 VOCs in vitro with a limited number of HLA alleles [11,46], an all-encompassing screen that covers the global human population would be far too time-consuming, laborious, and expensive to generate using wet lab approaches, necessitating the need for AI-based in silico profiling approaches.

Here we provide an AI-generated resource of mutated epitopes from all current VOCs, as well as the Omicron BA2 subvariant, from the perspective of their antigen presentation potential that can be used as a proxy for immunogenicity. The insights gained from analyzing this resource correlate with the early empirical findings from wet-lab-based studies that demonstrated that the T cell responses induced by vaccination and natural infection (from other VOCs) remain cross reactive against the Omicron VOC. The in silico evidence we present here highlights this trend more comprehensively across all possible mutated epitopes in all VOCs, and across the most frequent HLA alleles in the human population. 

This finding not only informs how we can track the correlates of immune protection across different vaccines and vaccine modalities, but also advocates the advancement of T cell-centric vaccine approaches to combat emerging VOCs [47]. Relying on the current spike protein vaccines for broad protection against VOCs is arguably not viable long-term; not only because the T cell epitope cargo is limited to a single viral protein that is subject to a high mutational rate, but also because it has been clearly demonstrated that T cell responses to the spike protein were reduced by approximately 50%, in 20% of naturally infected or vaccinated individuals [43].

This resource and analysis provided us the opportunity to automatically assess the difference in immunogenic potential in candidate epitopes between a VOC lineage, a mutant strain, and the originating SARS-CoV-2 lineage identified in Wuhan by examining the potential T cell antigen drift of the current and emerging VOCs. We demonstrated the robustness of the majority of identified T-cell epitopes across all VOCs using several distance metrics; however, the Wasserstein distance and the average difference in AP score were the most informative.

Using both the Wasserstein distance and the average difference in AP score, we were able to examine different properties of the VOC AP score distributions. The average difference in AP score was easy to interpret and gave us a direction for the potential antigenic drift; however, it did not consider the shape of the two AP score distributions. The Wasserstein distance did not give a direction for the antigenic drift; however, it considered the shape of the two distributions aiding their comparison. Based on the observations presented, one can argue that the Wasserstein distance and the average difference in AP scores are very similar, so it might be sufficient to just examine the average difference in AP score. However, we still recommend examining both to cover a wider array of patterns in the distributions. Moreover, it seemed like no specific HLA populations are more affected than others, and generally the differences between the mutant and wildtype per allele were very small.

The results in this study, demonstrate comprehensively, that significant antigenic drift resulting in escape against a pre-existing (natural- or vaccine-induced) T-cell response is unlikely to emerge in the existing VOCs. The analysis we outlined here, such as delta calculations of AP potential between a VOC-mutated epitope and its corresponding wildtype, serves as an index of T cell antigenic drift or T-cell immunogenicity for emerging lineages of SARS-CoV-2. This AI-generated index may guide the development of T cell vaccines and diagnostics that are even more robust against current and future SARS-CoV-2 VOCs, and their emerging subvariants.

## Figures and Tables

**Table 1:** Different metrics summarizing the difference in AP scores between mutant and wildtype peptides within a VOC. Three different methods were used to compare the distributions: (i) the average difference in AP scores, (ii) the two-sample Kolmogorov–Smirnov (KS) test, (iii) and the Wasserstein distance.

VOC	Avg. AP Score Diff. (Mutant–Wildtype)	Two-Sample Kolmogorov–Smirnov Test		Wasserstein Distance
		Statistic	*p*-Value	
Alpha	0.0092	0.0381	1.4631 × 10^−9^	0.0092
Beta	0.0034	0.0147	0.0798	0.0037
Gamma	0.0134	0.06	3.3352 × 10^−27^	0.0134
Delta	−0.0008	0.0305	4.7707 × 10^−6^	0.0089
Omicron	0.0069	0.0292	4.0882 × 10^−16^	0.0071

**Table 2:** Number of peptides with AP score >0.5 in either the mutant or the corresponding wildtype for each of the VOCs and the fraction they represent out of the Wuhan Wildtype. “Wuhan Wildtype” is the number of peptides with antigen presentation score >0.5 in the complete original Wuhan strain.

VOC	Nb. of Non-Synonymous Mutations	Nb. of Mutated Peptides	Fraction of All Wildtype Peptides in the Original Wuhan Strain
Alpha	21	14,485	0.0173
Beta	22	14,890	0.0178
Gamma	25	17,106	0.0204
Delta	22	13,952	0.0167
Omicron	59	42,356	0.0506
Wildtype (Wuhan)	NA	837,211	1

**Table 3:** Mean, 5th (for the average AP score difference only), 25th 75th, and 95th percentiles of the Wasserstein distance, of the two-sided Kolmogorov–Smirnov statistic, and of the average difference in AP score between mutant and wildtype epitopes across the HLA alleles for each VOC.

VOC	Avg. AP Score Diff. (Mutant–Wildtype)					Two-Sample Kolmogorov–Smirnov Test Statistic				Wasserstein Distance			
	Mean	Percentile				Mean	Percentile			Mean	Percentile		
		5th	25th	75th	95th		25th	75th	95th		25th	75th	95th
Alpha	0.0127	−0.0224	−0.0006	0.0217	0.065	0.1898	0.1011	0.2391	0.4779	0.0285	0.0138	0.0355	0.0729
Beta	0.0028	−0.0478	−0.0094	0.0152	0.0596	0.1935	0.0956	0.2362	0.475	0.0316	0.0136	0.0412	0.0863
Gamma	0.0151	−0.0522	0.0012	0.0266	0.0622	0.1738	0.1042	0.2301	0.475	0.0268	0.0148	0.0318	0.0959
Delta	0.0006	−0.017	−0.018	0.0129	0.0564	0.1919	0.114	0.2287	0.3333	0.0343	0.0181	0.0393	0.0595
Omicron	0.0074	−0.0302	−0.0054	0.0204	0.05	0.1473	0.0781	0.2	0.3333	0.0251	0.0115	0.0377	0.0556

**Table 4:** Top three HLA alleles that present the biggest Wasserstein distance for each of the VOC and their corresponding haplotype according to The Allele Frequency Net Database (AFND). The last column shows in which populations the set of HLA alleles is presented with the highest frequency. For the cells denoted by an * it means that the AFND database only provided results for one of the top three most affected HLA alleles.

VOC	HLA Allele	Wasserstein Distance	Populations Highest Allele Frequency (AFND)
Alpha	HLA-A*01:02	0.121	Brazil, Australia, Azores
	HLA-A*25:01	0.092	
	HLA-B*27:05	0.092	
Beta	HLA-B*27:06	0.143	Australia, Brazil
	HLA-B*48:01	0.115	
	HLA-A*25:01	0.113	
Gamma	HLA-A*25:01	0.089	Australia, Austria, Azores (results based only on HLA-A*25:01) *
	HLA-B*18:02	0.088	
	HLA-A*33:03	0.079	
Delta	HLA-B*46:01	0.159	China, Hong Kong, Malaysia (results based only on HLA-B*46:01) *
	HLA-B*27:03	0.152	
	HLA-B*27:05	0.152	
Omicron	HLA-B*27:02	0.110	Bulgaria, Germany, Israel (results based only on HLA-B*27:02)
	HLA-B*18:02	0.104	
	HLA-A*24:02	0.077	

**Table 5:** Wasserstein distance and average difference between the distribution of AP scores for the mutant and corresponding wildtype peptides for all HLA-A and HLA-B alleles.

VOC	HLA Type	Avg. AP Score Difference (Mutant–Wildtype)	Wasserstein Distance
Alpha	HLA-A	0.0126	0.0126
	HLA-B	0.0064	0.0065
Beta	HLA-A	0.0038	0.0039
	HLA-B	0.0031	0.0045
Gamma	HLA-A	0.0185	0.0185
	HLA-B	0.0093	0.0093
Delta	HLA-A	−0.0004	0.0118
	HLA-B	−0.0011	0.0071
Omicron	HLA-A	0.0132	0.0133
	HLA-B	0.0017	0.0025

## Supplementary Materials

The following supporting information can be downloaded at: https://www.mdpi.com/article/10.3390/vaccines10071123/s1.
