# Supplementary material for: The T Cell Epitope Landscape of SARS-CoV-2 Variants of Concern
Source: Vaccines (Basel). 2022 Jul 14;10(7):1123. doi: 10.3390/vaccines10071123 (PMC9315645; doi:10.3390/vaccines10071123)
Supplement: Supplementary file 1 [file vaccines-10-01123-s001.zip › Tennoe_et_al_Supplementary_File2.pdf]

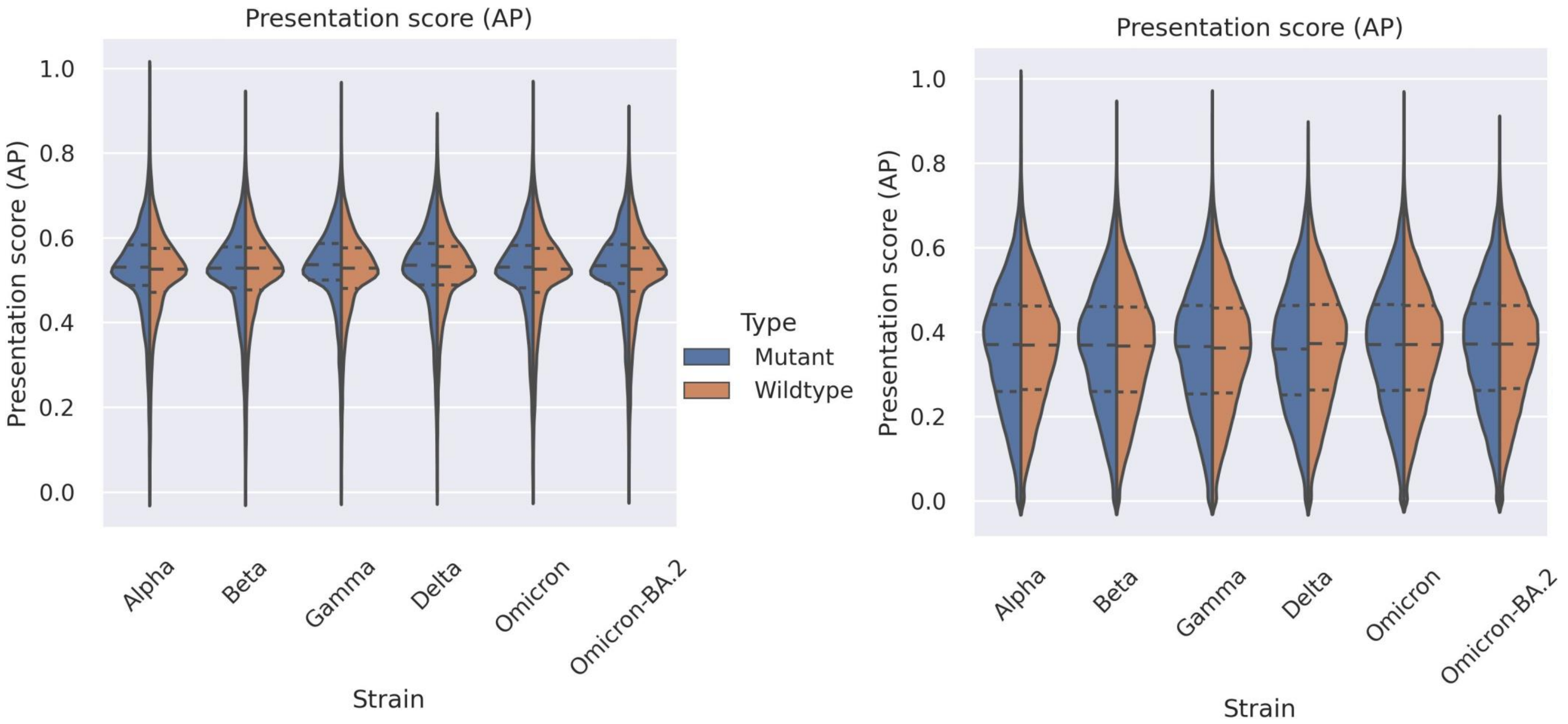

Supplementary Figures S1-S2: Violin plots of the distribution of antigen presentation (AP) scores for the SARS-CoV2 variants (mutant) and the original Wuhan lineage (wildtype) epitopes. The Omicron BA.2 subvariant was also included along with the variants of concern. The dashed lines within each violin represent the quartiles. For the left plots, only entries with an AP score above 0.5 in either the mutant or wildtype were considered, while for the right plots, all AP score entries (i.e., no filtering) were considered.

Supplementary Figure S3: Violin plots of the distribution of presentation scores (AP) for mutant and wildtype epitopes per allele for each VOC and Omicron-BA.2. The dashed lines within each violin represent the quartiles. Only entries with an AP score above 0.5 in either the mutant or wildtype were considered for this plot.

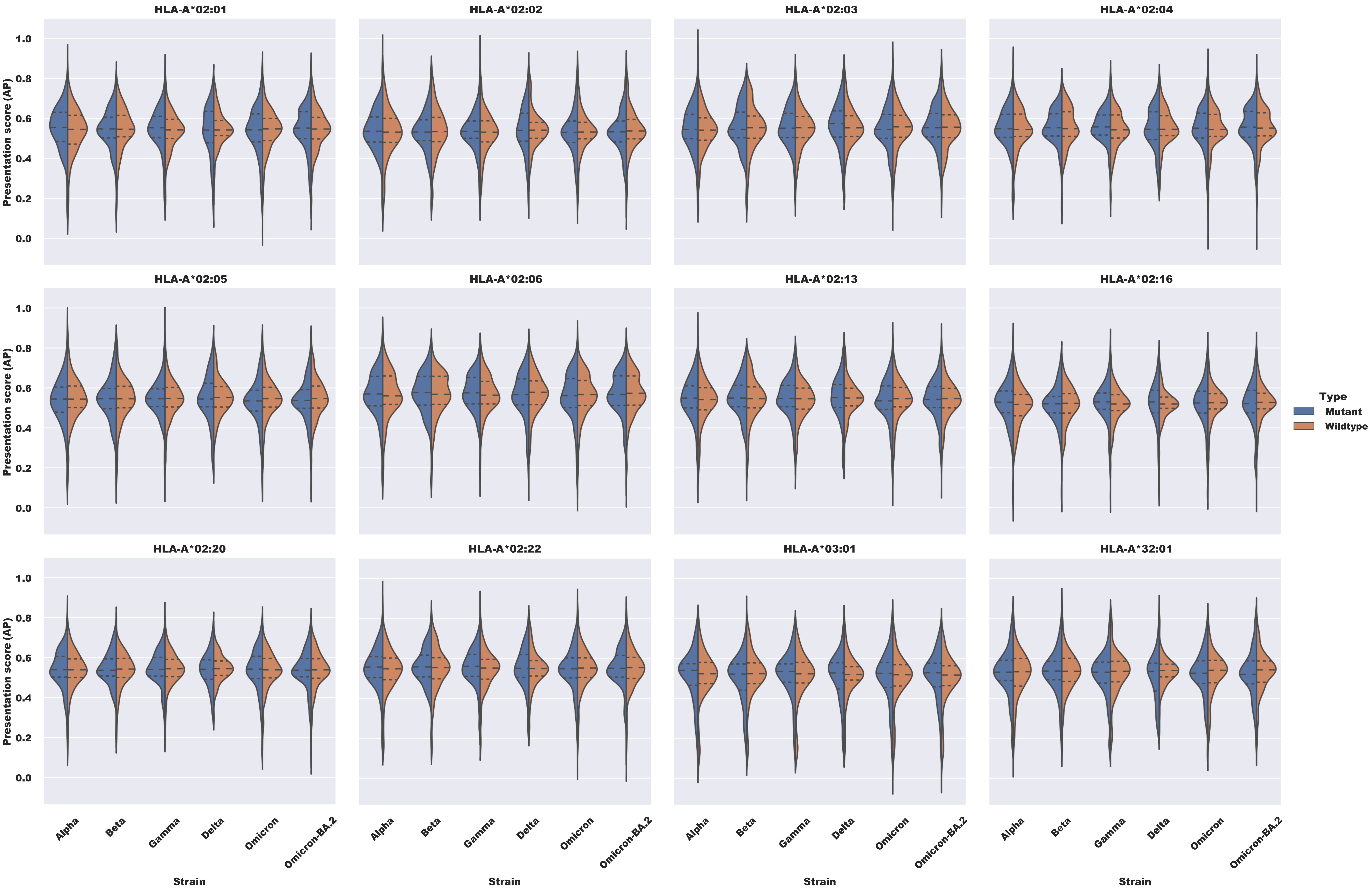

Supplementary Figure S3: Violin plots of the distribution of presentation scores (AP) for mutant and wildtype epitopes per allele for each VOC and Omicron-BA.2. The dashed lines within each violin represent the quartiles. Only entries with an AP score above 0.5 in either the mutant or wildtype were considered for this plot.

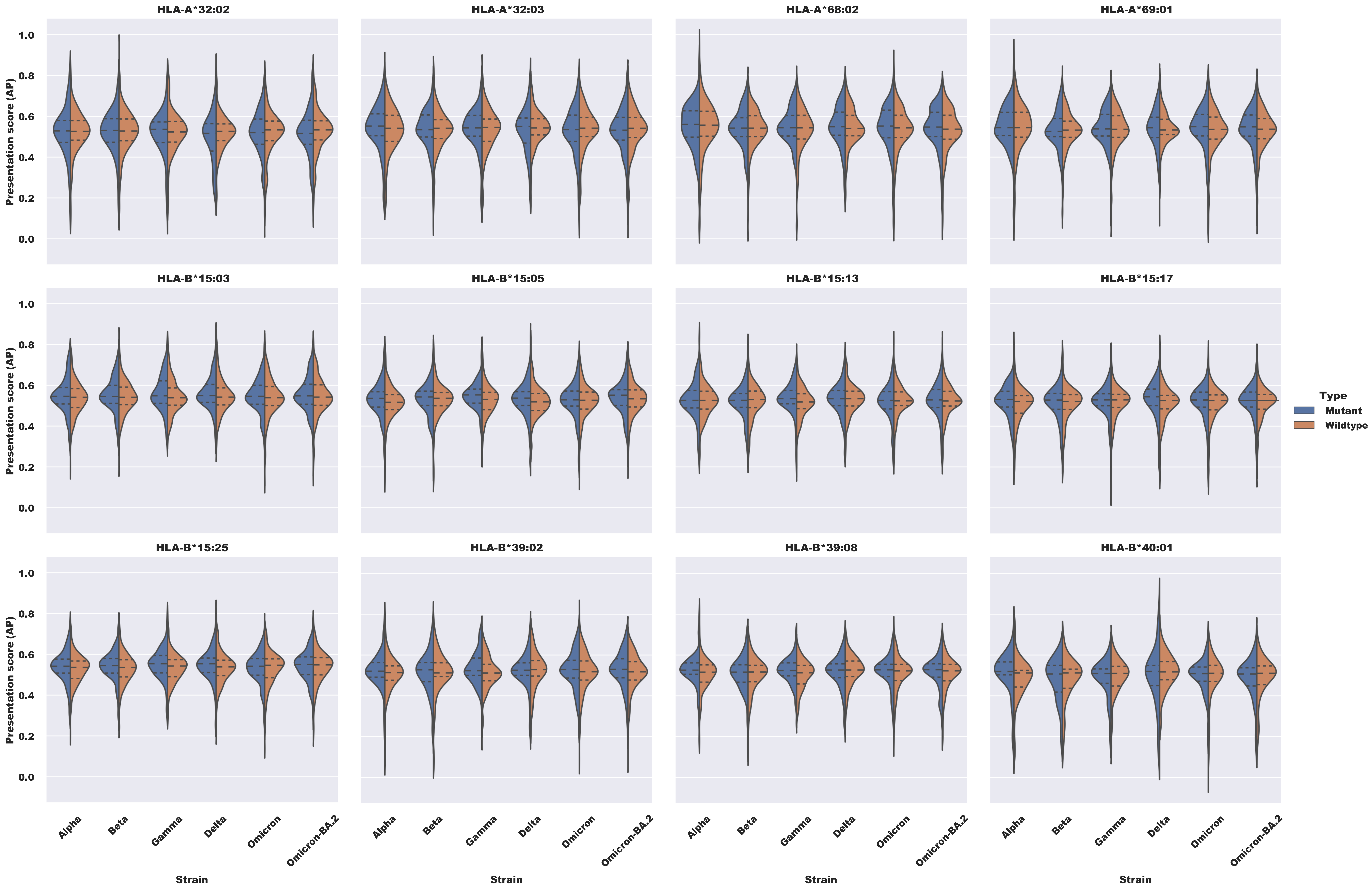

Supplementary Figure S3: Violin plots of the distribution of presentation scores (AP) for mutant and wildtype epitopes per allele for each VOC and Omicron-BA.2. The dashed lines within each violin represent the quartiles. Only entries with an AP score above 0.5 in either the mutant or wildtype were considered for this plot.

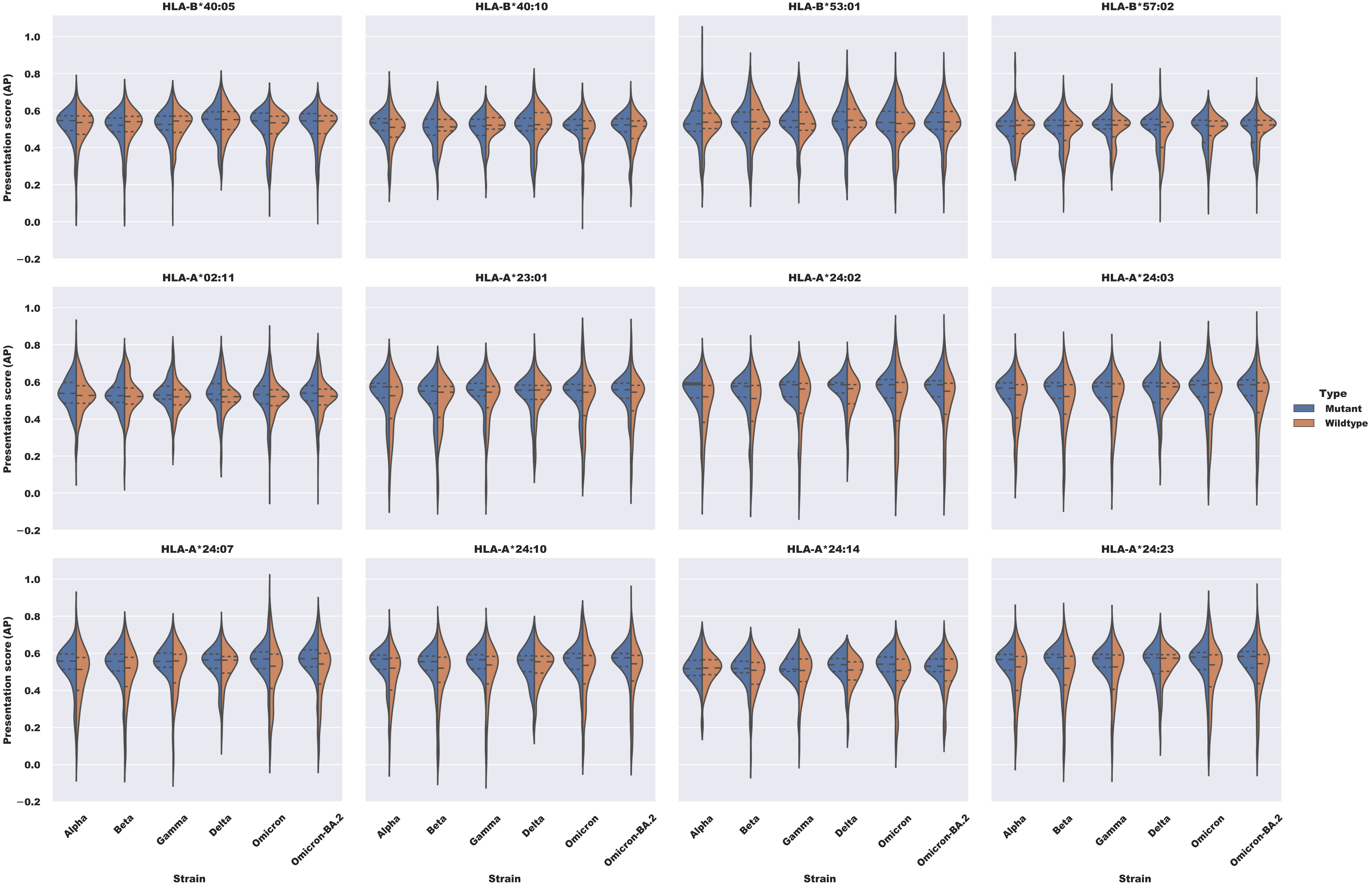

Supplementary Figure S3: Violin plots of the distribution of presentation scores (AP) for mutant and wildtype epitopes per allele for each VOC and Omicron-BA.2. The dashed lines within each violin represent the quartiles. Only entries with an AP score above 0.5 in either the mutant or wildtype were considered for this plot.

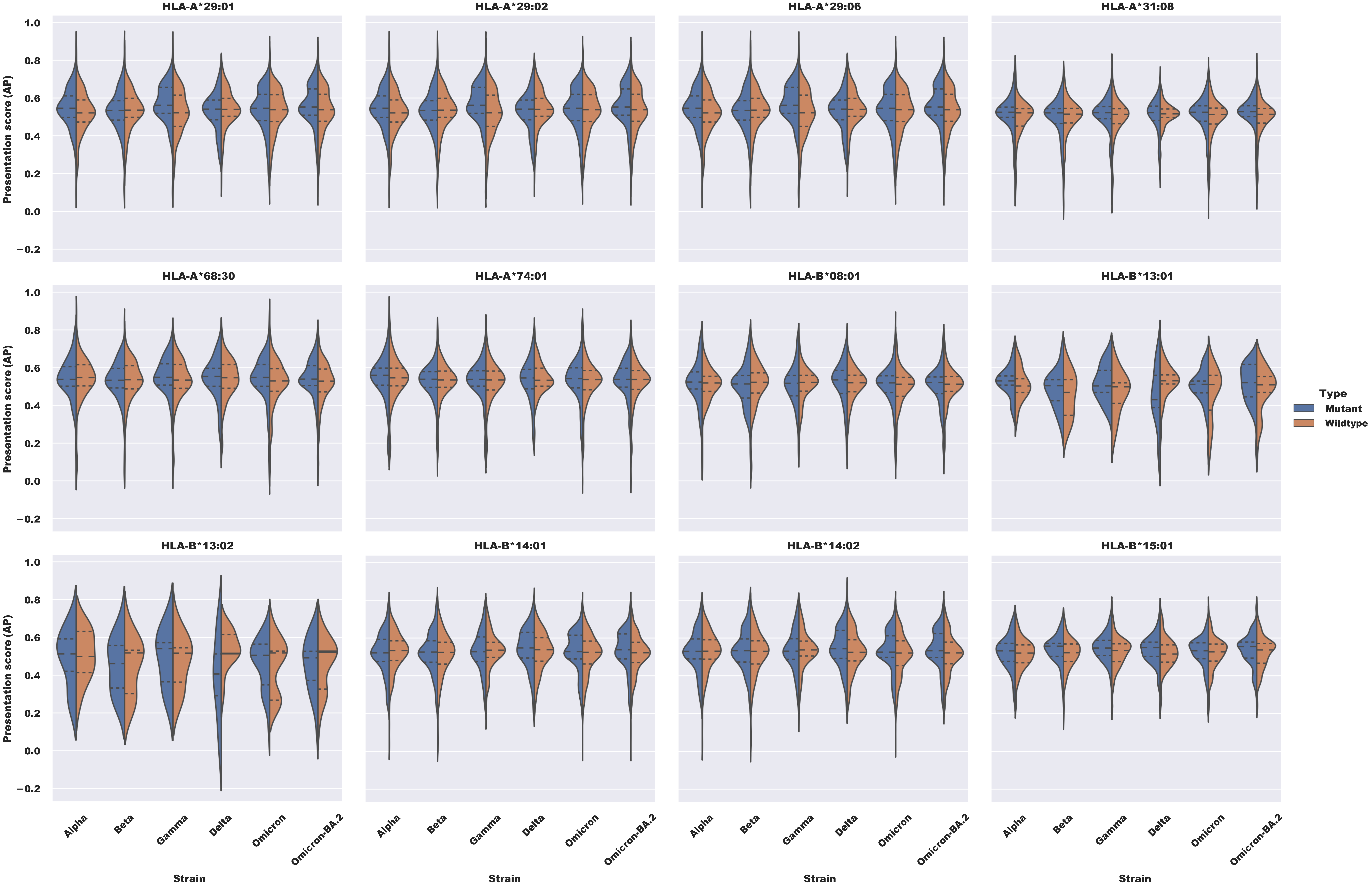

Supplementary Figure S3: Violin plots of the distribution of presentation scores (AP) for mutant and wildtype epitopes per allele for each VOC and Omicron-BA.2. The dashed lines within each violin represent the quartiles. Only entries with an AP score above 0.5 in either the mutant or wildtype were considered for this plot.

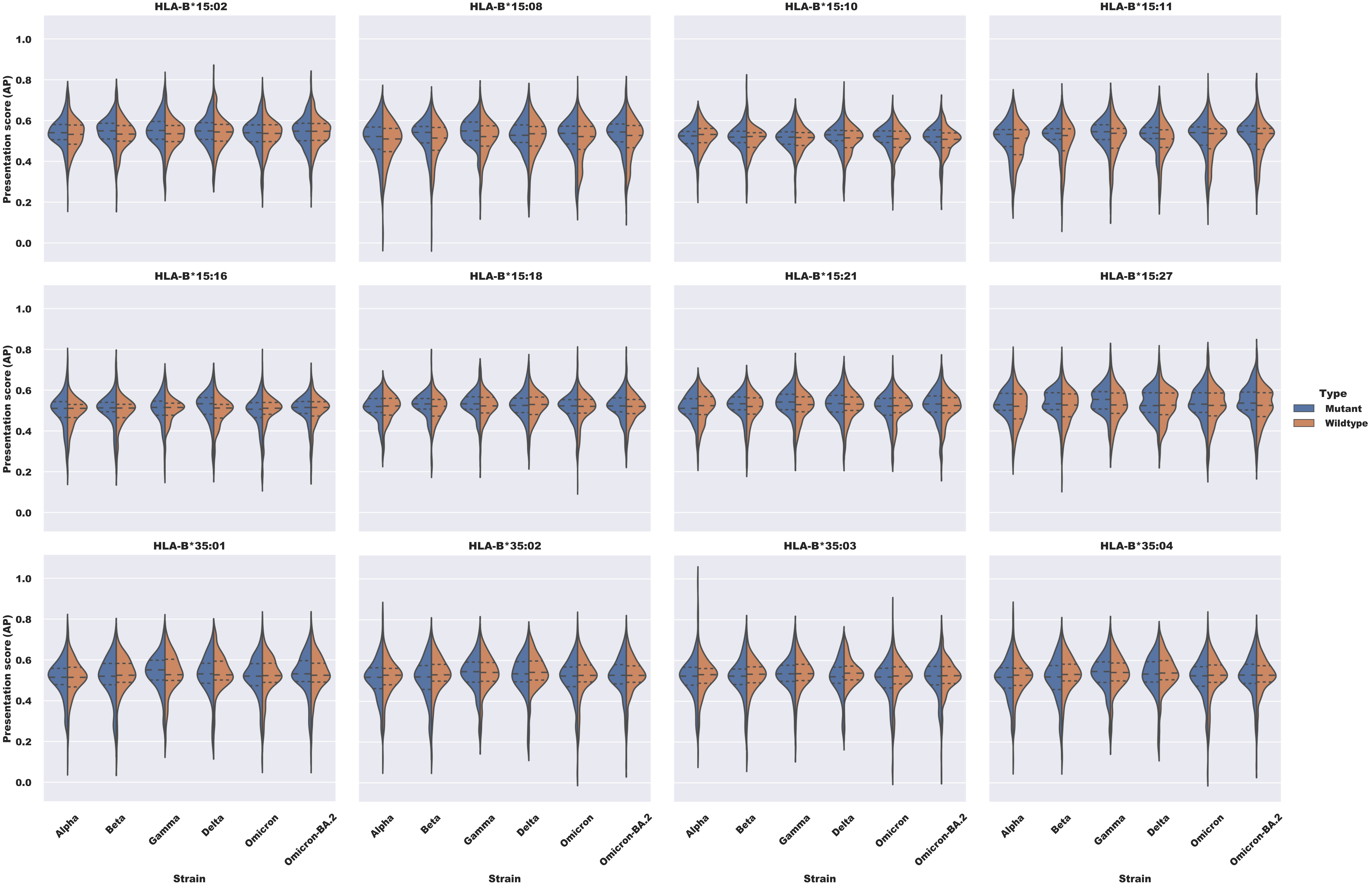

Supplementary Figure S3: Violin plots of the distribution of presentation scores (AP) for mutant and wildtype epitopes per allele for each VOC and Omicron-BA.2. The dashed lines within each violin represent the quartiles. Only entries with an AP score above 0.5 in either the mutant or wildtype were considered for this plot.

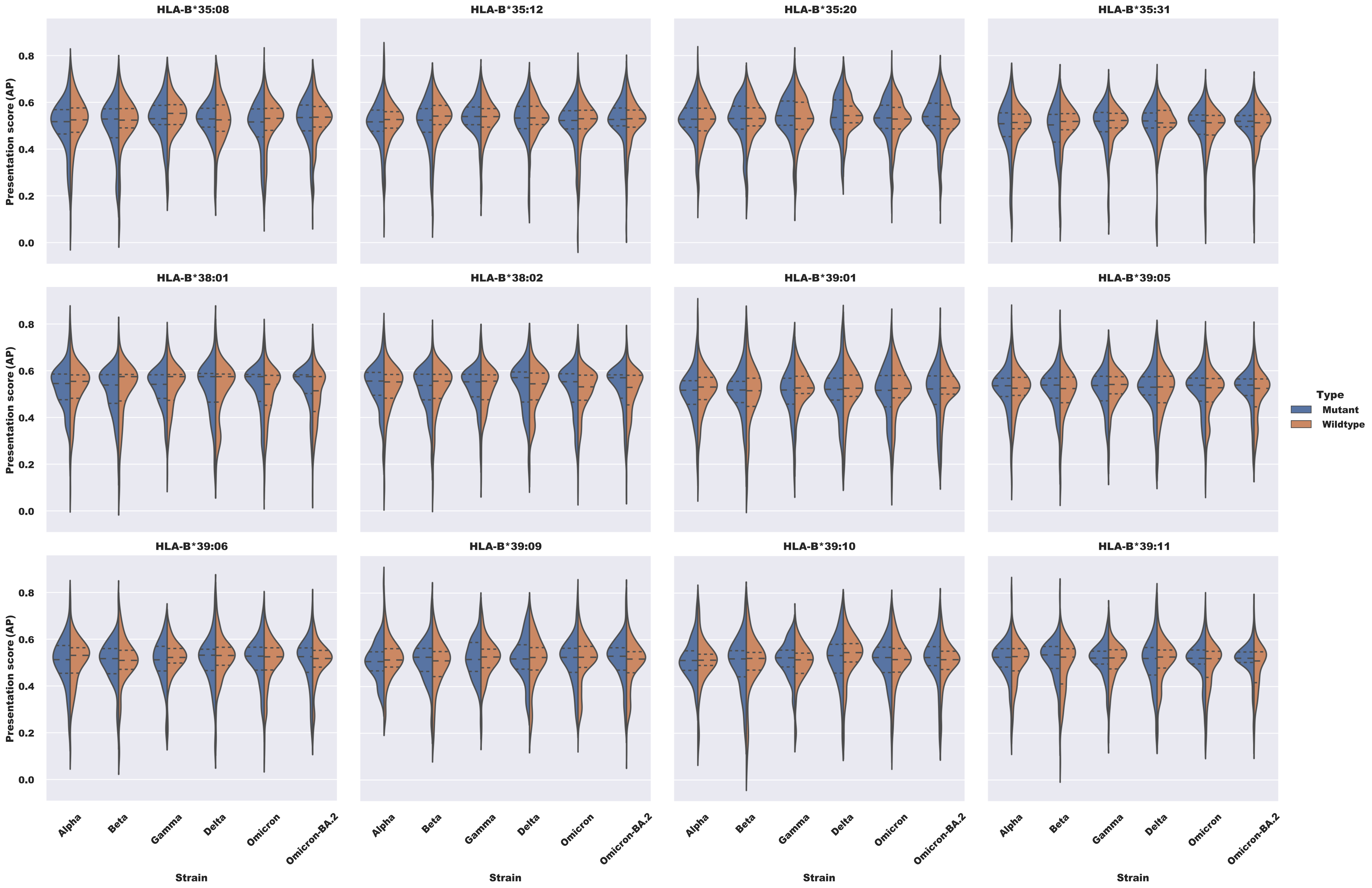

Supplementary Figure S3: Violin plots of the distribution of presentation scores (AP) for mutant and wildtype epitopes per allele for each VOC and Omicron-BA.2. The dashed lines within each violin represent the quartiles. Only entries with an AP score above 0.5 in either the mutant or wildtype were considered for this plot.

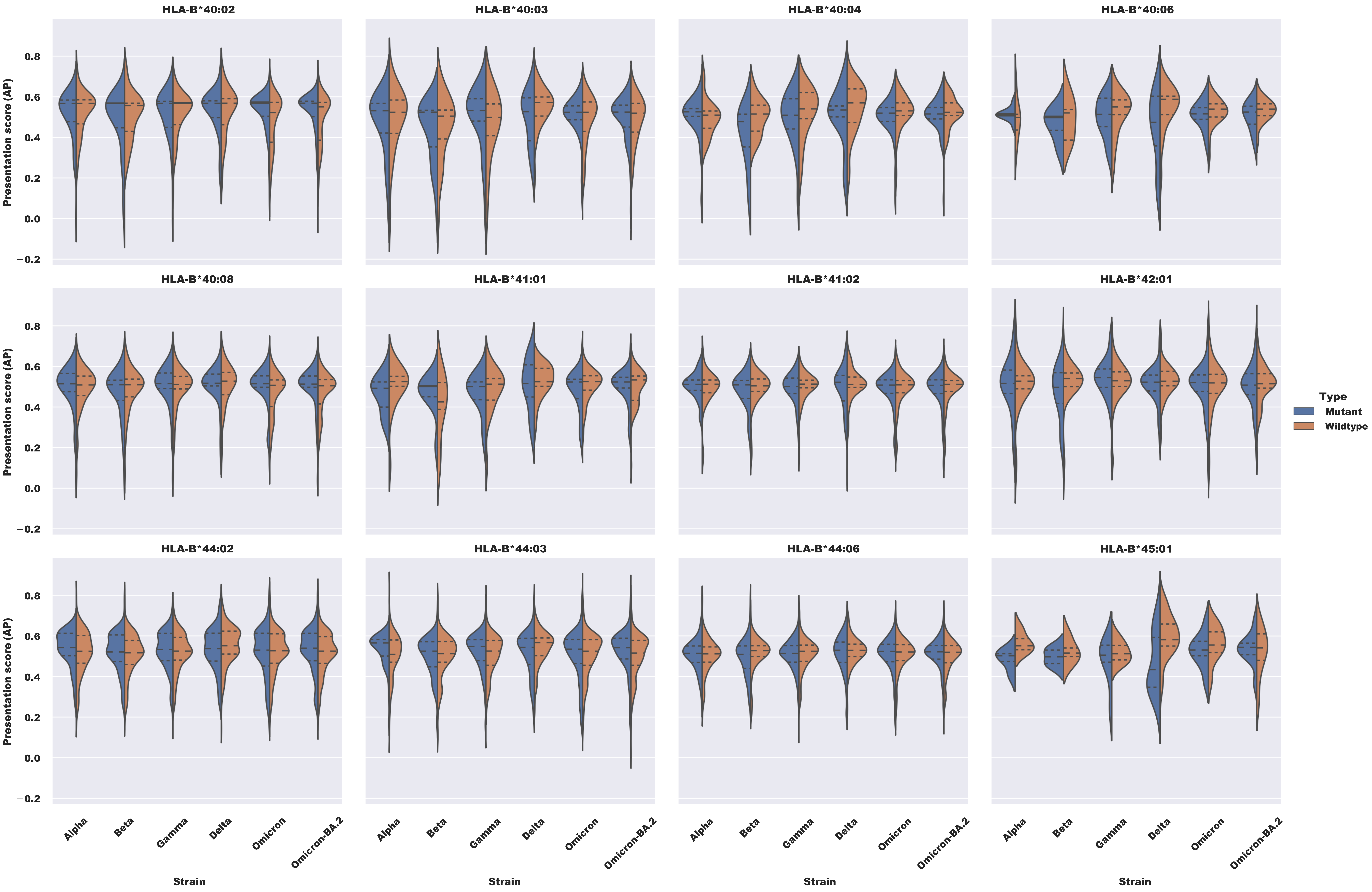

Supplementary Figure S3: Violin plots of the distribution of presentation scores (AP) for mutant and wildtype epitopes per allele for each VOC and Omicron-BA.2. The dashed lines within each violin represent the quartiles. Only entries with an AP score above 0.5 in either the mutant or wildtype were considered for this plot.

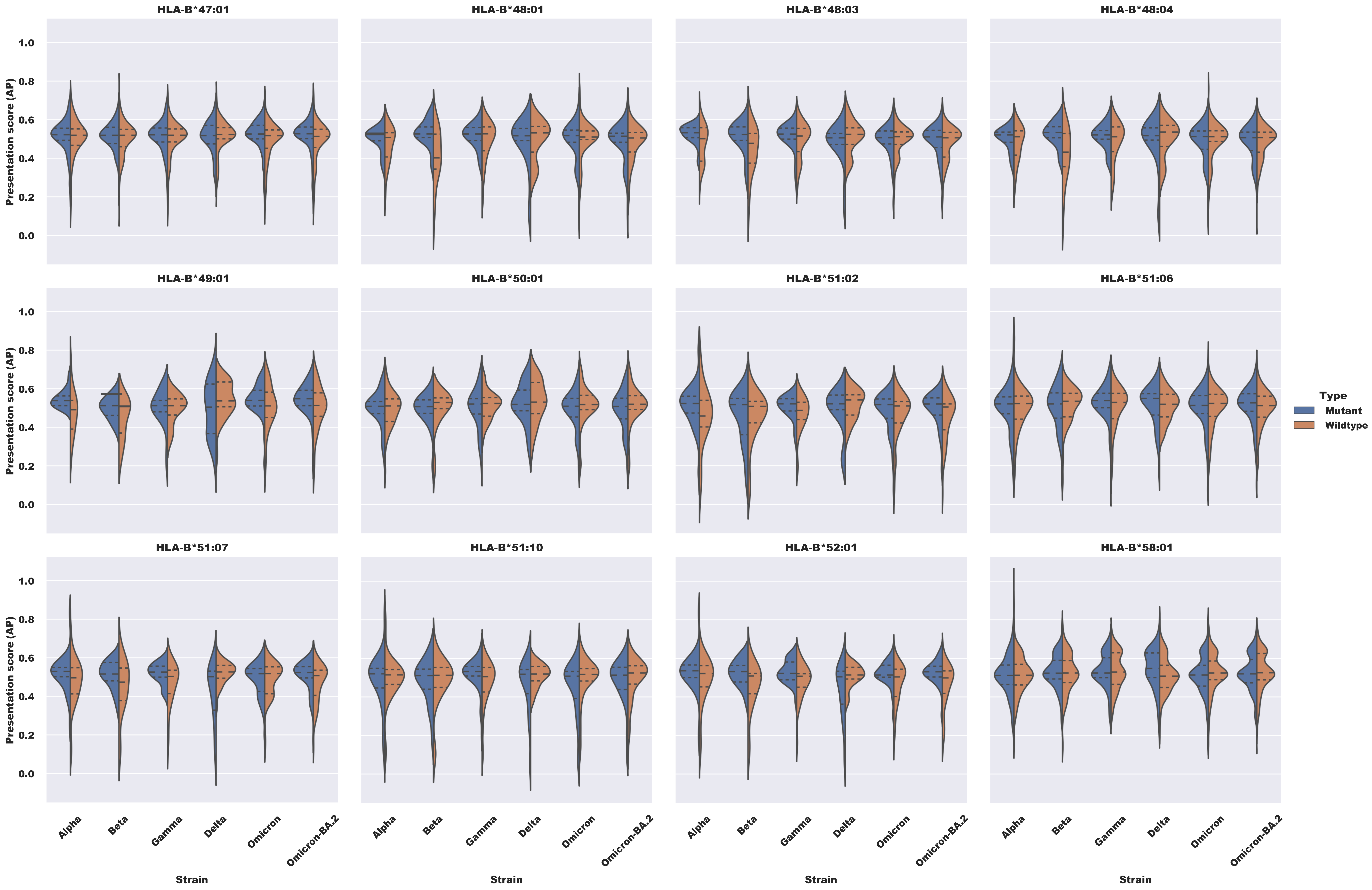

Supplementary Figure S3: Violin plots of the distribution of presentation scores (AP) for mutant and wildtype epitopes per allele for each VOC and Omicron-BA.2. The dashed lines within each violin represent the quartiles. Only entries with an AP score above 0.5 in either the mutant or wildtype were considered for this plot.

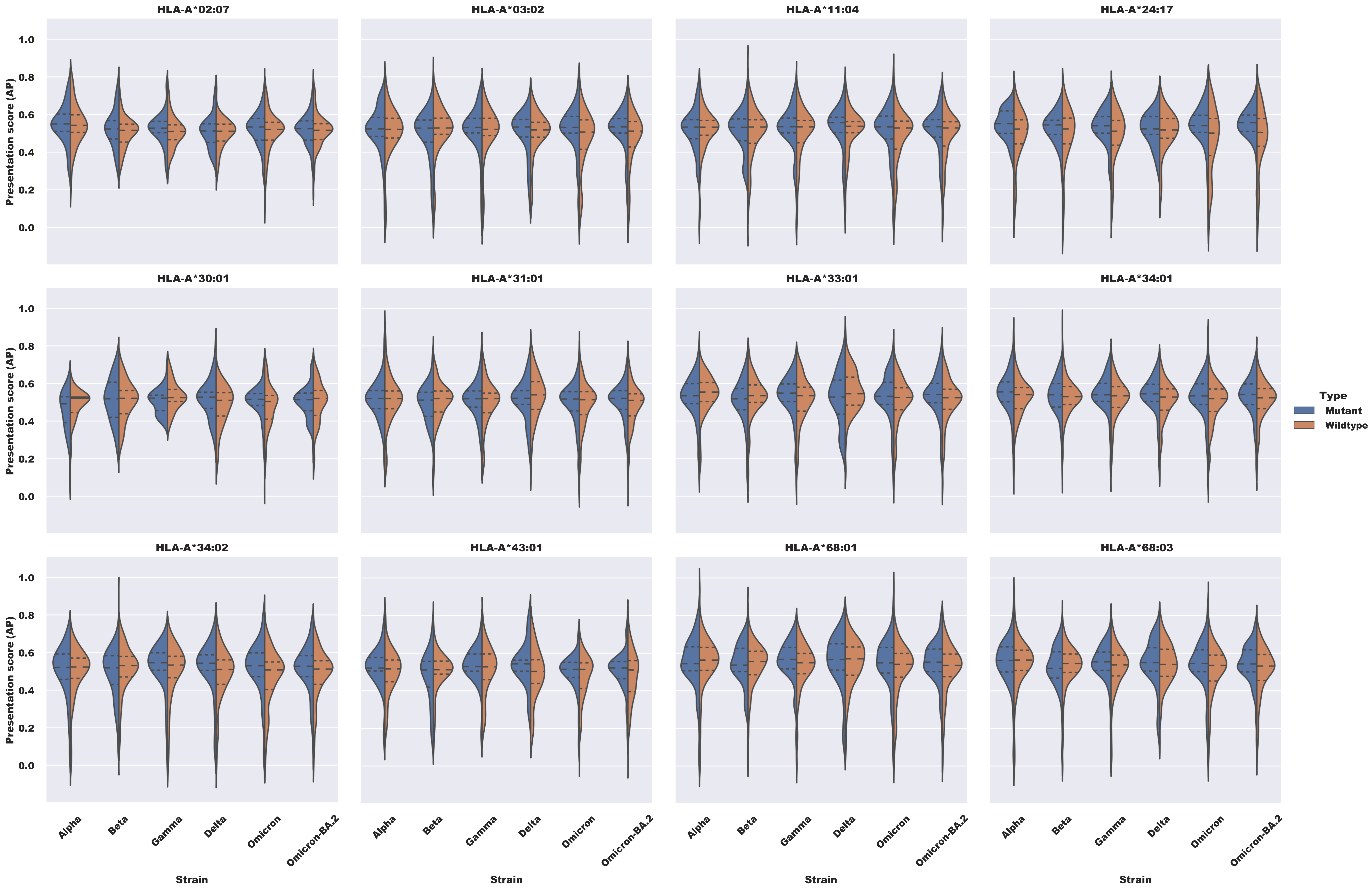

Supplementary Figure S3: Violin plots of the distribution of presentation scores (AP) for mutant and wildtype epitopes per allele for each VOC and Omicron-BA.2. The dashed lines within each violin represent the quartiles. Only entries with an AP score above 0.5 in either the mutant or wildtype were considered for this plot.

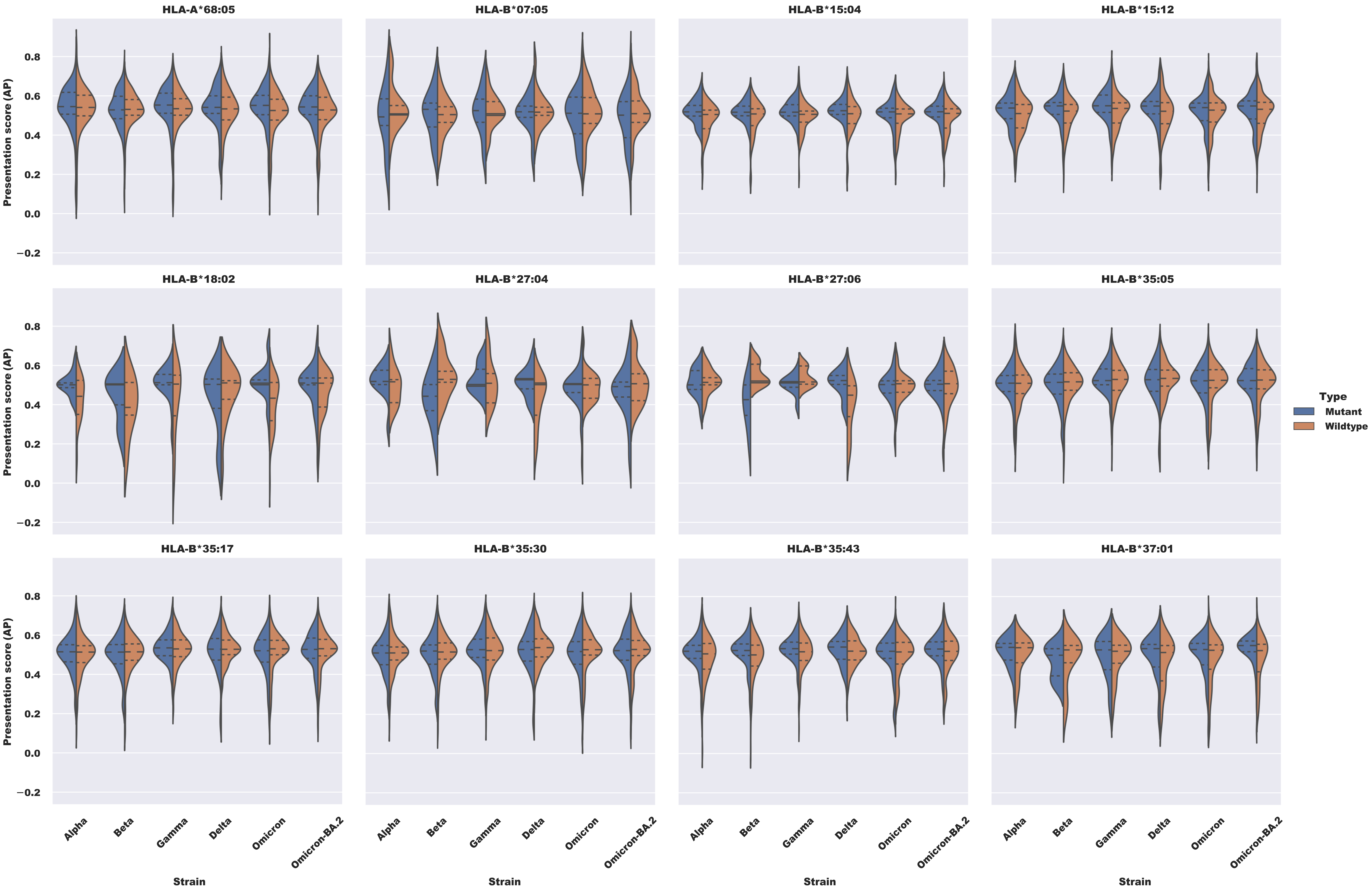

Supplementary Figure S3: Violin plots of the distribution of presentation scores (AP) for mutant and wildtype epitopes per allele for each VOC and Omicron-BA.2. The dashed lines within each violin represent the quartiles. Only entries with an AP score above 0.5 in either the mutant or wildtype were considered for this plot.

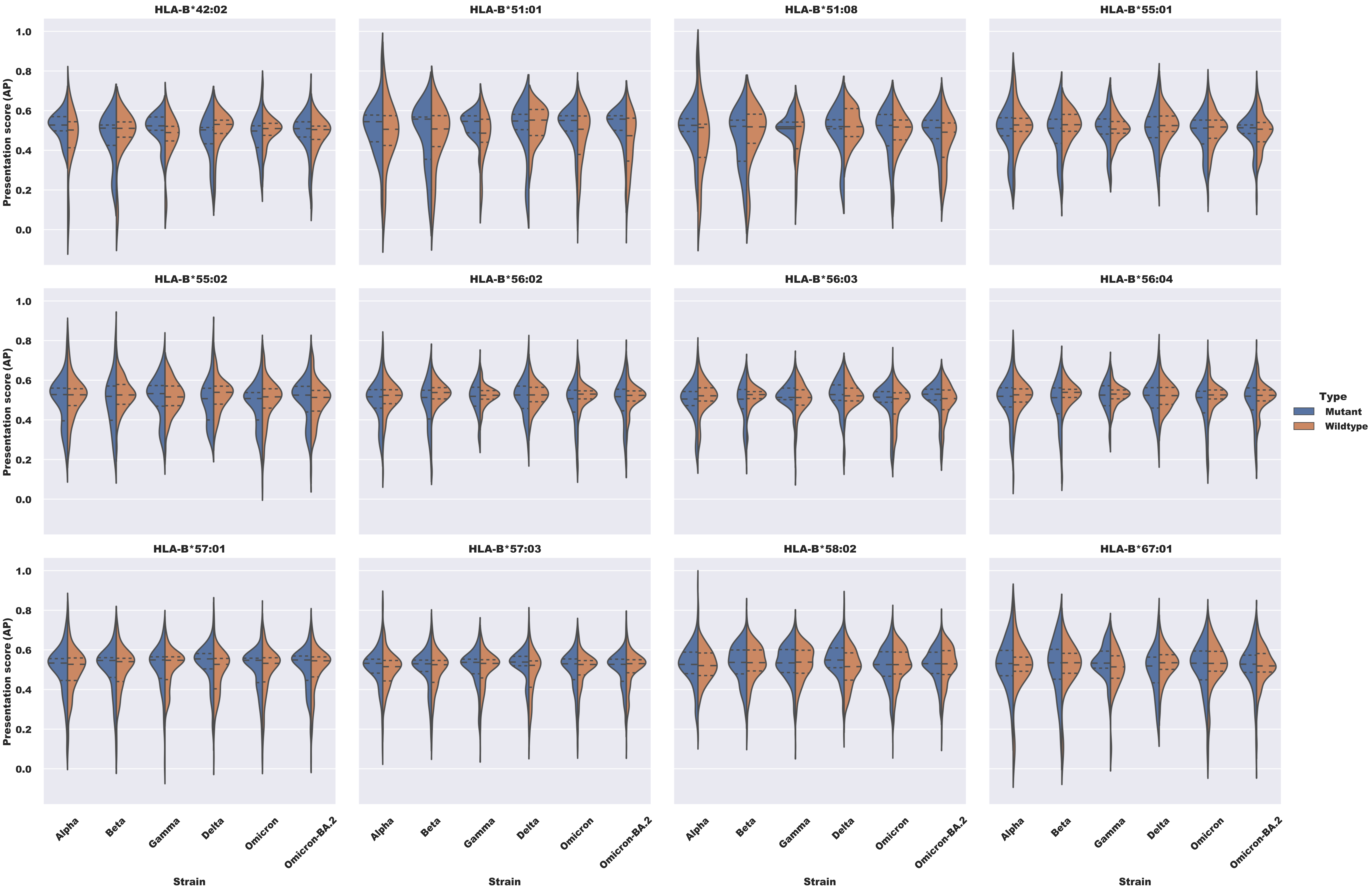

Supplementary Figure S3: Violin plots of the distribution of presentation scores (AP) for mutant and wildtype epitopes per allele for each VOC and Omicron-BA.2. The dashed lines within each violin represent the quartiles. Only entries with an AP score above 0.5 in either the mutant or wildtype were considered for this plot.

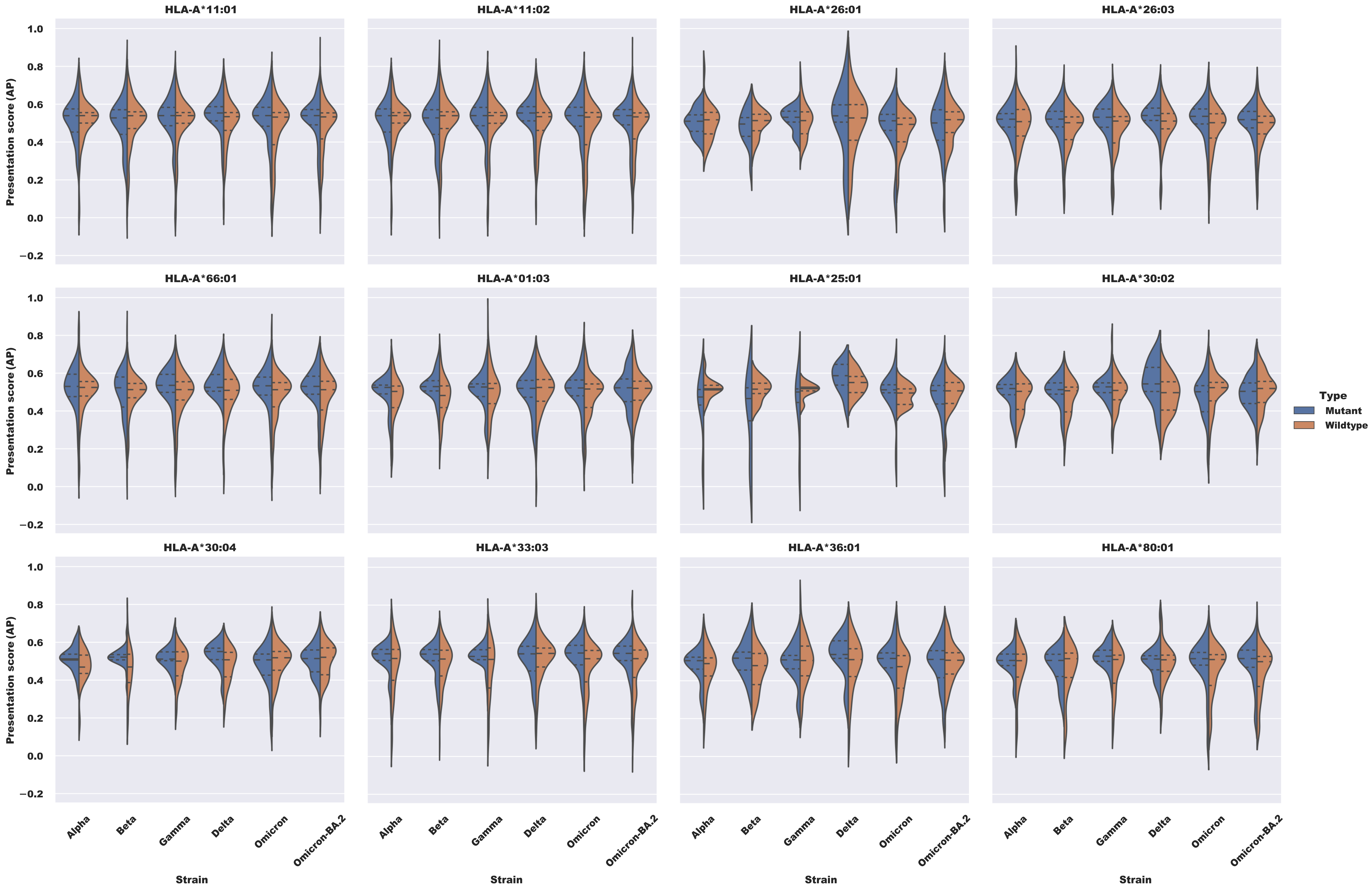

Supplementary Figure S3: Violin plots of the distribution of presentation scores (AP) for mutant and wildtype epitopes per allele for each VOC and Omicron-BA.2. The dashed lines within each violin represent the quartiles. Only entries with an AP score above 0.5 in either the mutant or wildtype were considered for this plot.

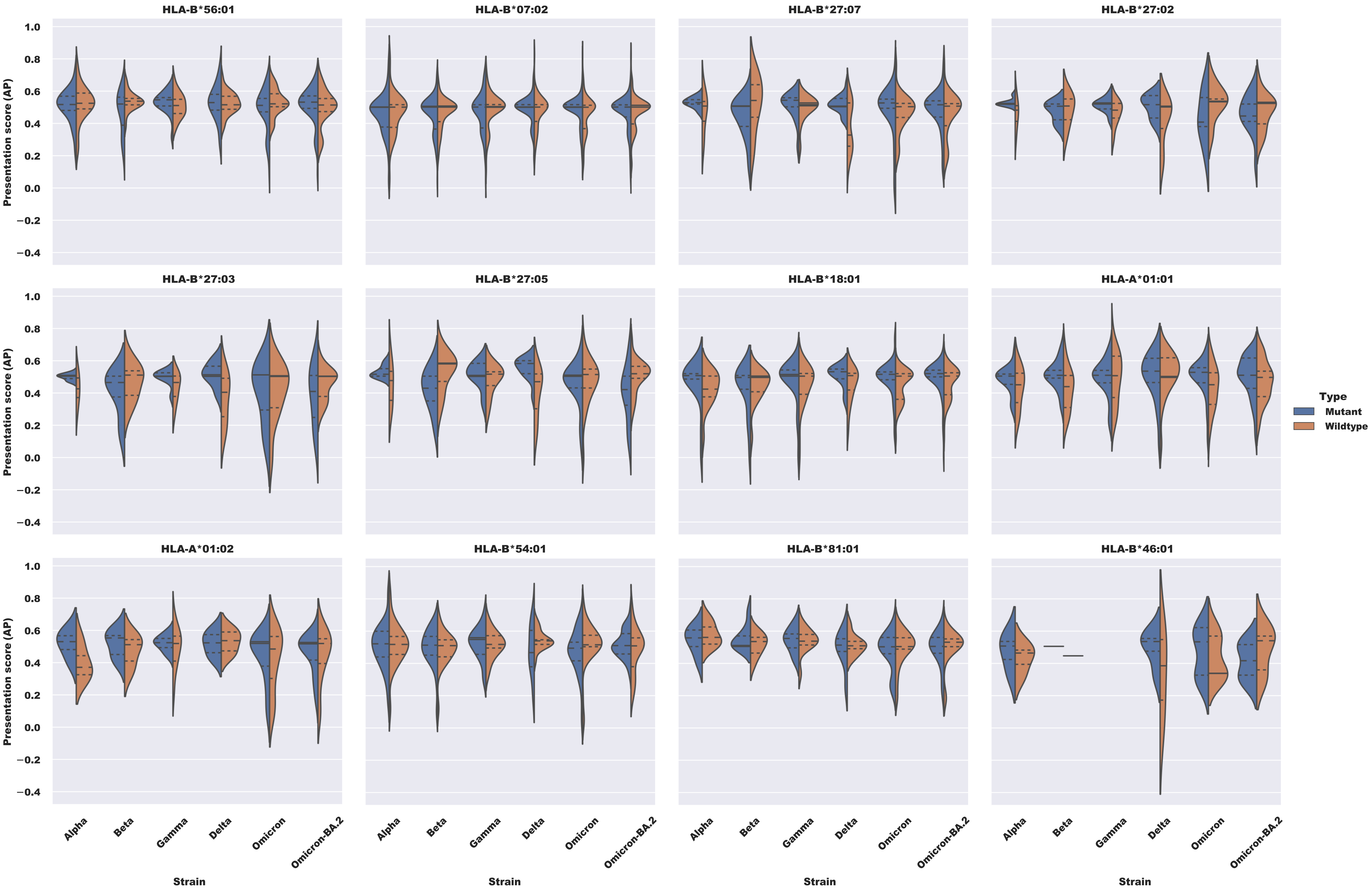

Supplementary Figure S4: Violin plots of the distribution of presentation scores (AP) for mutant and wildtype epitopes per allele for each VOC and Omicron-BA.2. The dashed lines within each violin represent the quartiles.

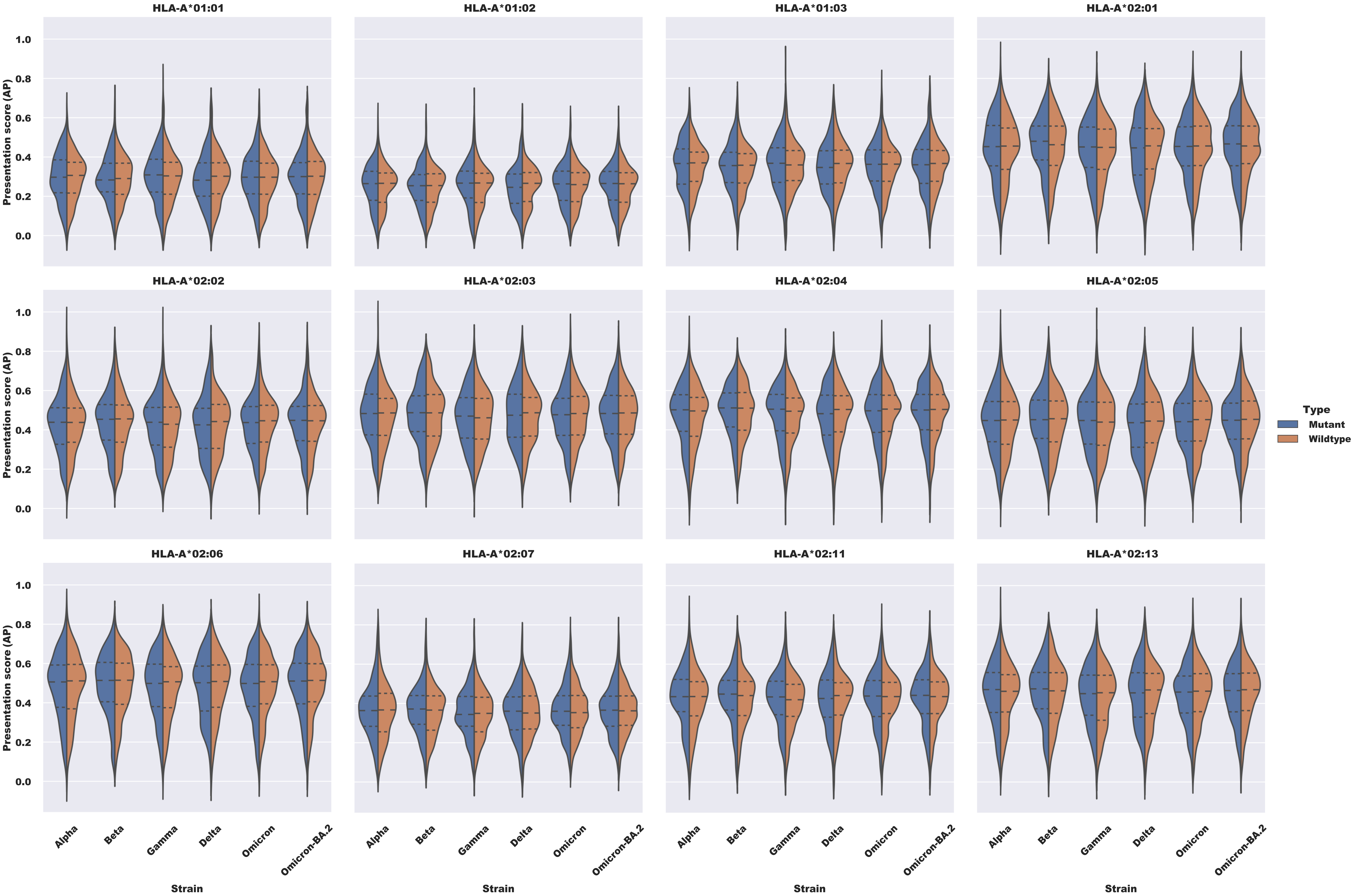

Supplementary Figure S4: Violin plots of the distribution of presentation scores (AP) for mutant and wildtype epitopes per allele for each VOC and Omicron-BA.2. The dashed lines within each violin represent the quartiles.

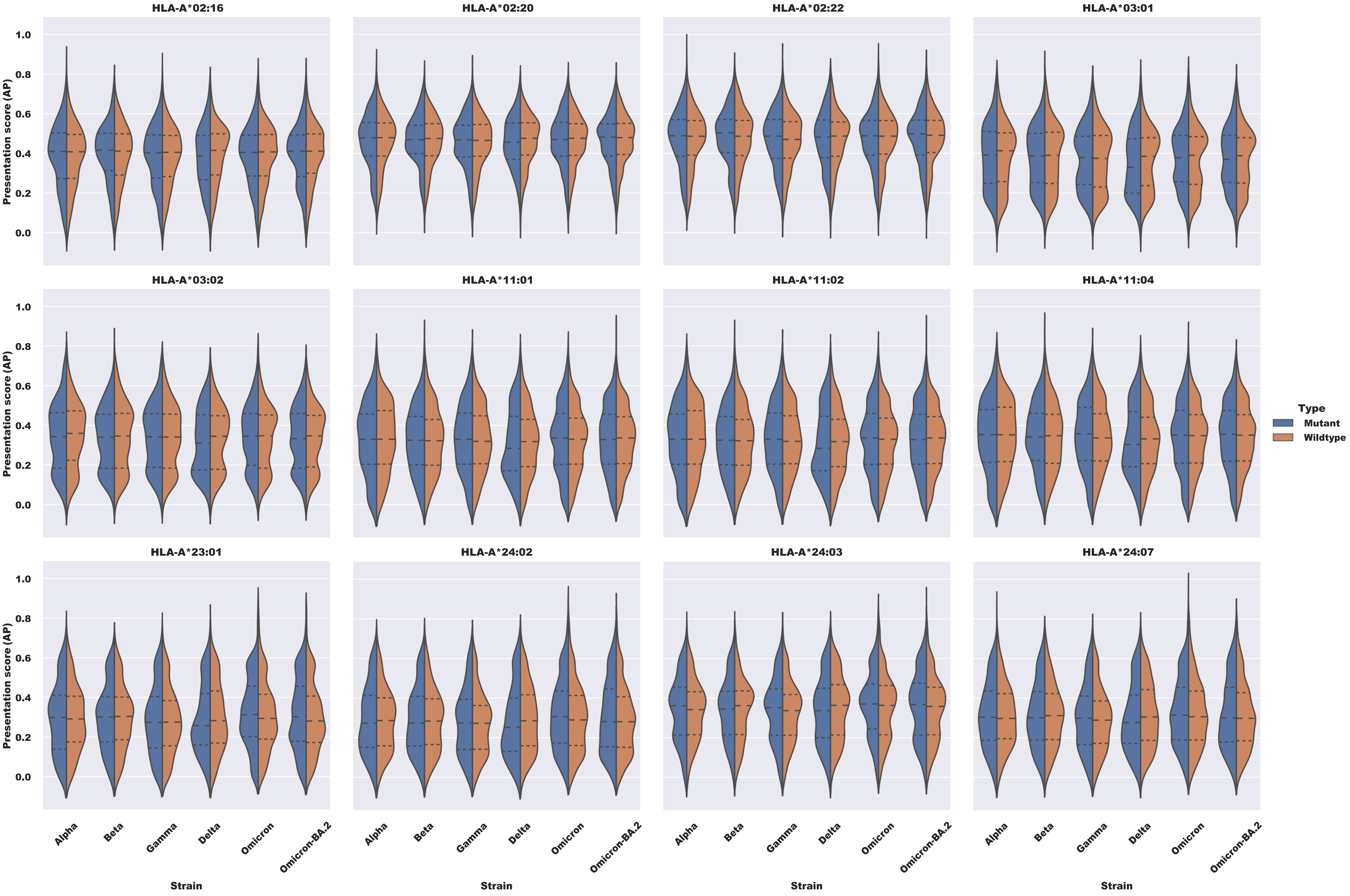

Supplementary Figure S4: Violin plots of the distribution of presentation scores (AP) for mutant and wildtype epitopes per allele for each VOC and Omicron-BA.2. The dashed lines within each violin represent the quartiles.

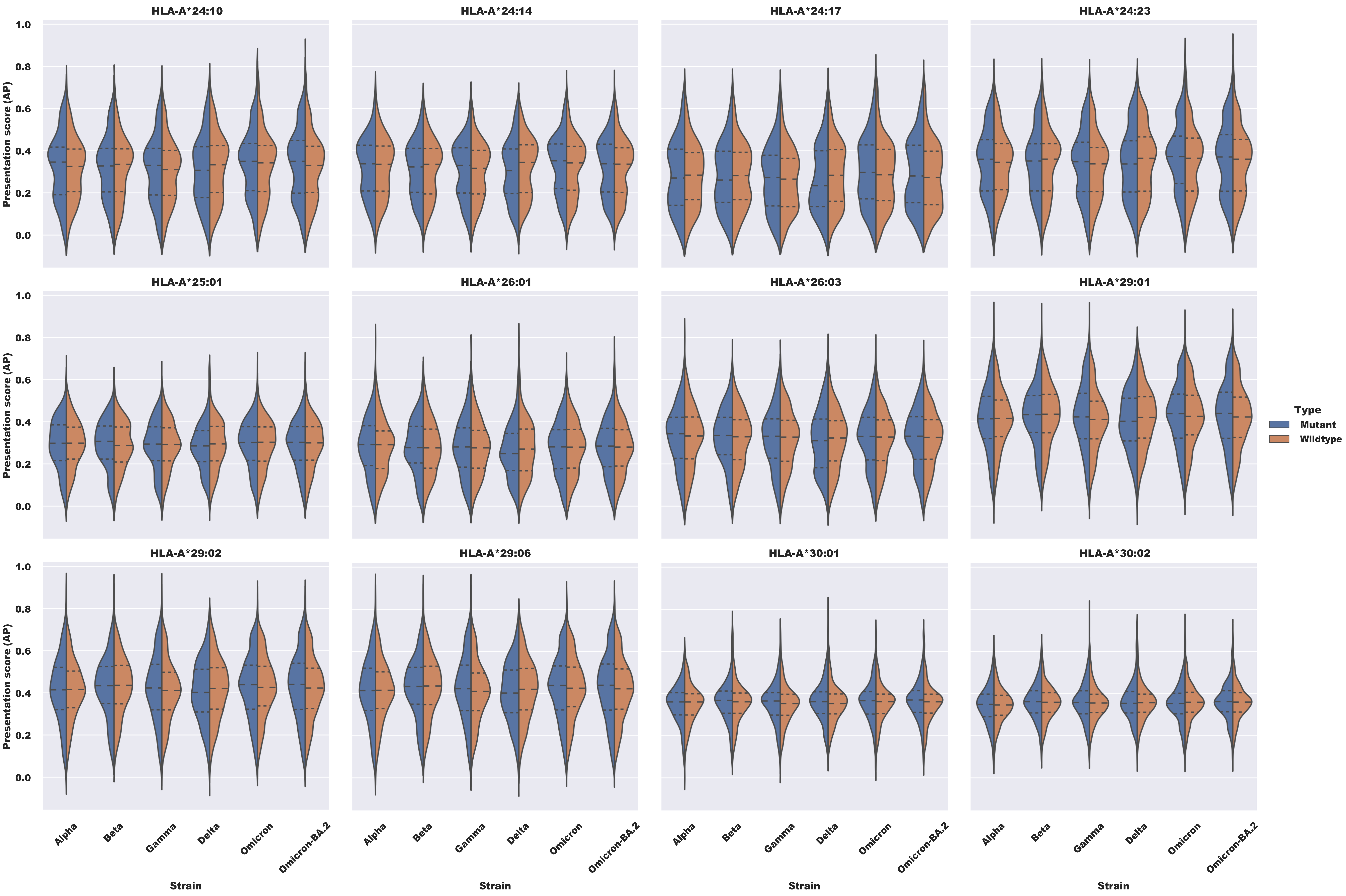

Supplementary Figure S4: Violin plots of the distribution of presentation scores (AP) for mutant and wildtype epitopes per allele for each VOC and Omicron-BA.2. The dashed lines within each violin represent the quartiles.

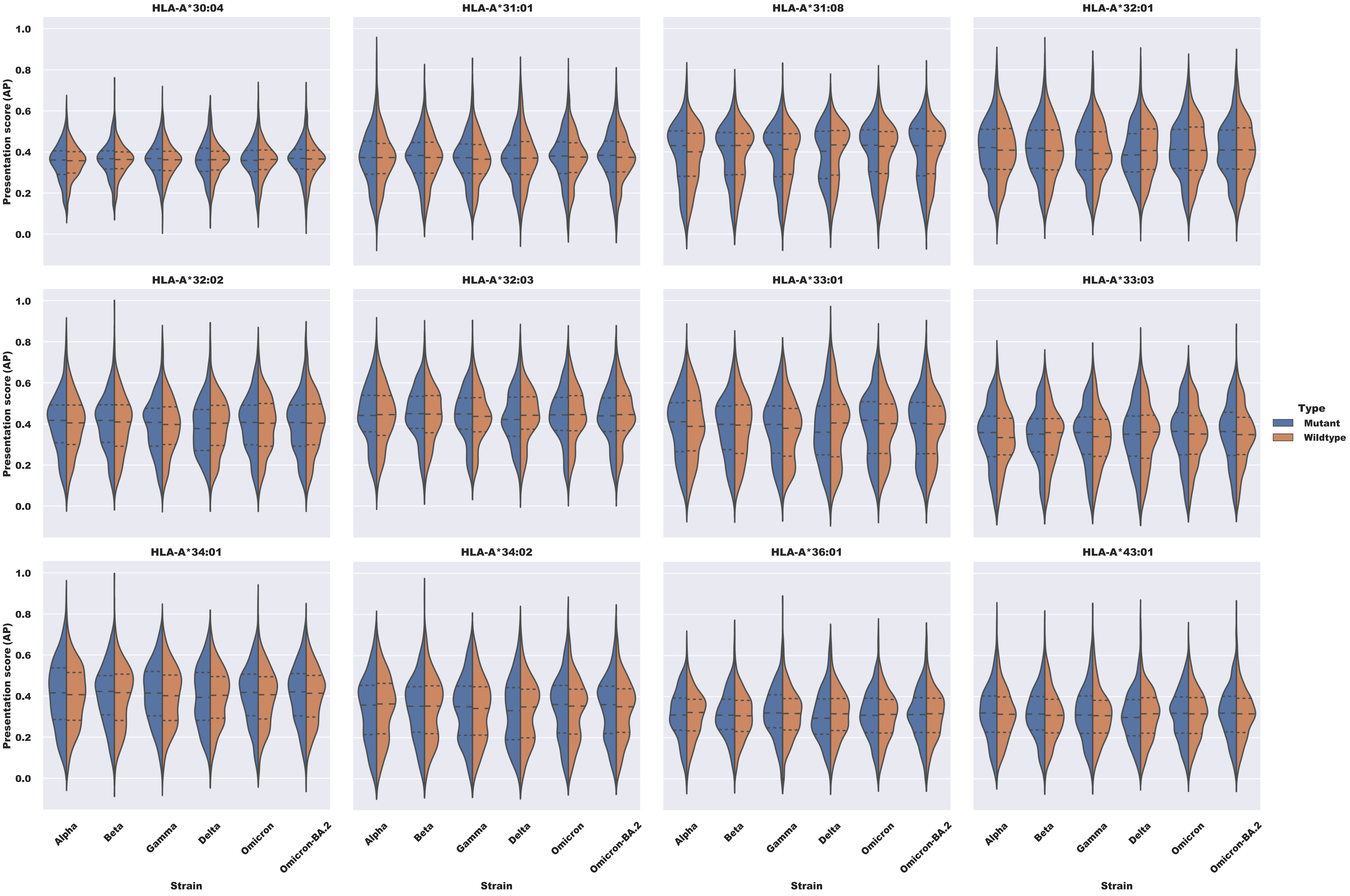

Supplementary Figure S4: Violin plots of the distribution of presentation scores (AP) for mutant and wildtype epitopes per allele for each VOC and Omicron-BA.2. The dashed lines within each violin represent the quartiles.

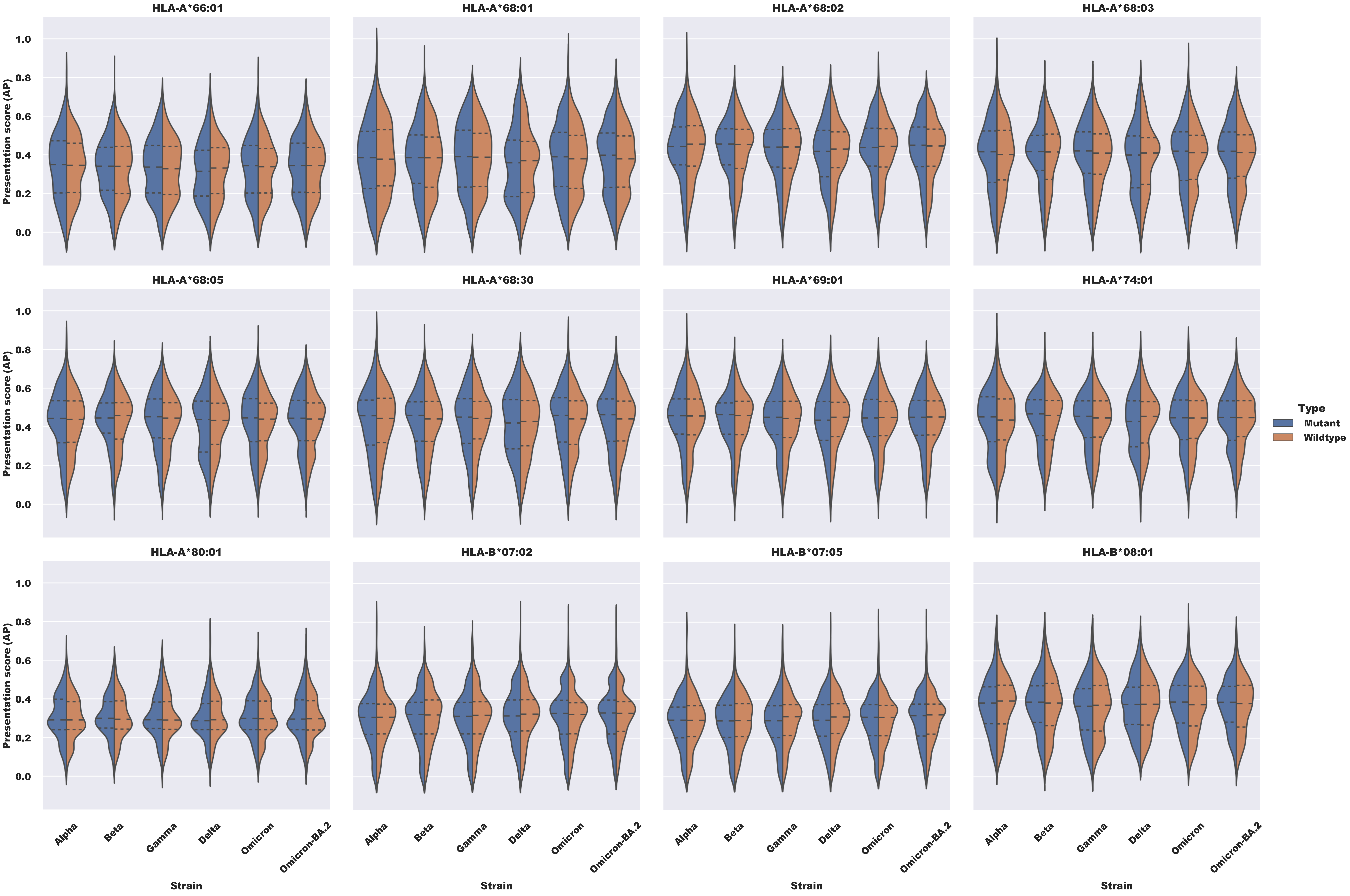

Supplementary Figure S4: Violin plots of the distribution of presentation scores (AP) for mutant and wildtype epitopes per allele for each VOC and Omicron-BA.2. The dashed lines within each violin represent the quartiles.

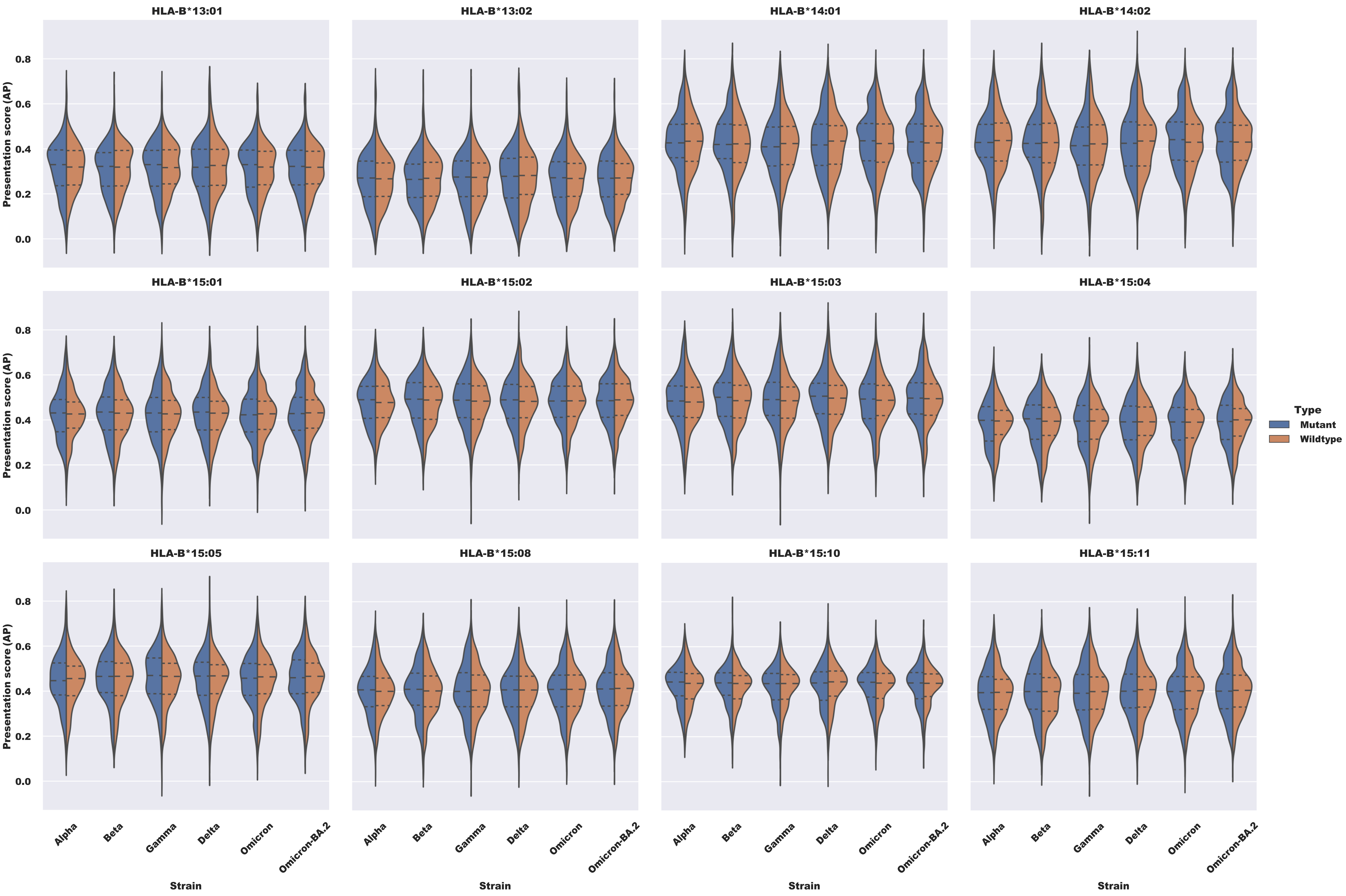

Supplementary Figure S4: Violin plots of the distribution of presentation scores (AP) for mutant and wildtype epitopes per allele for each VOC and Omicron-BA.2. The dashed lines within each violin represent the quartiles.

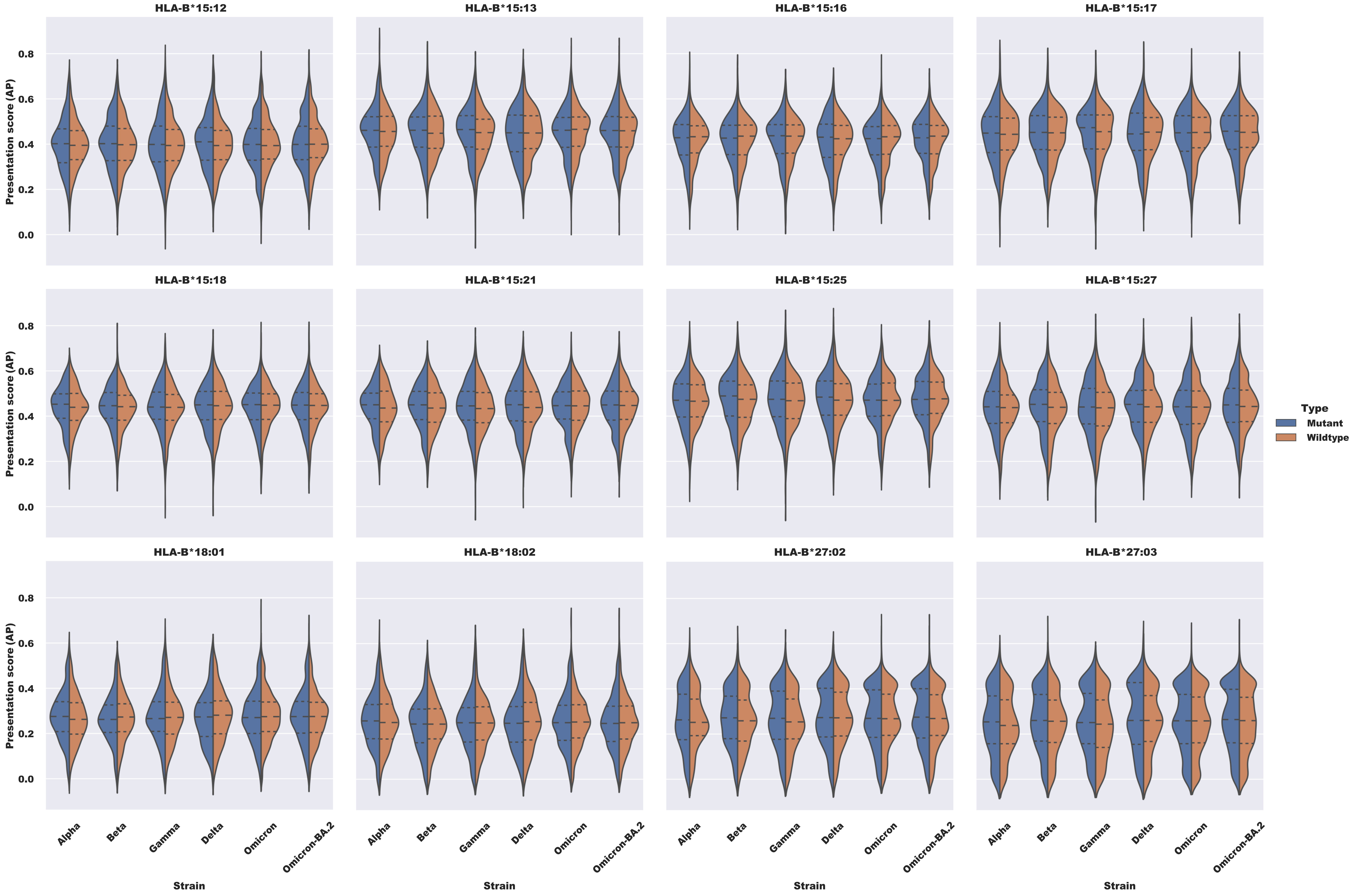

Supplementary Figure S4: Violin plots of the distribution of presentation scores (AP) for mutant and wildtype epitopes per allele for each VOC and Omicron-BA.2. The dashed lines within each violin represent the quartiles.

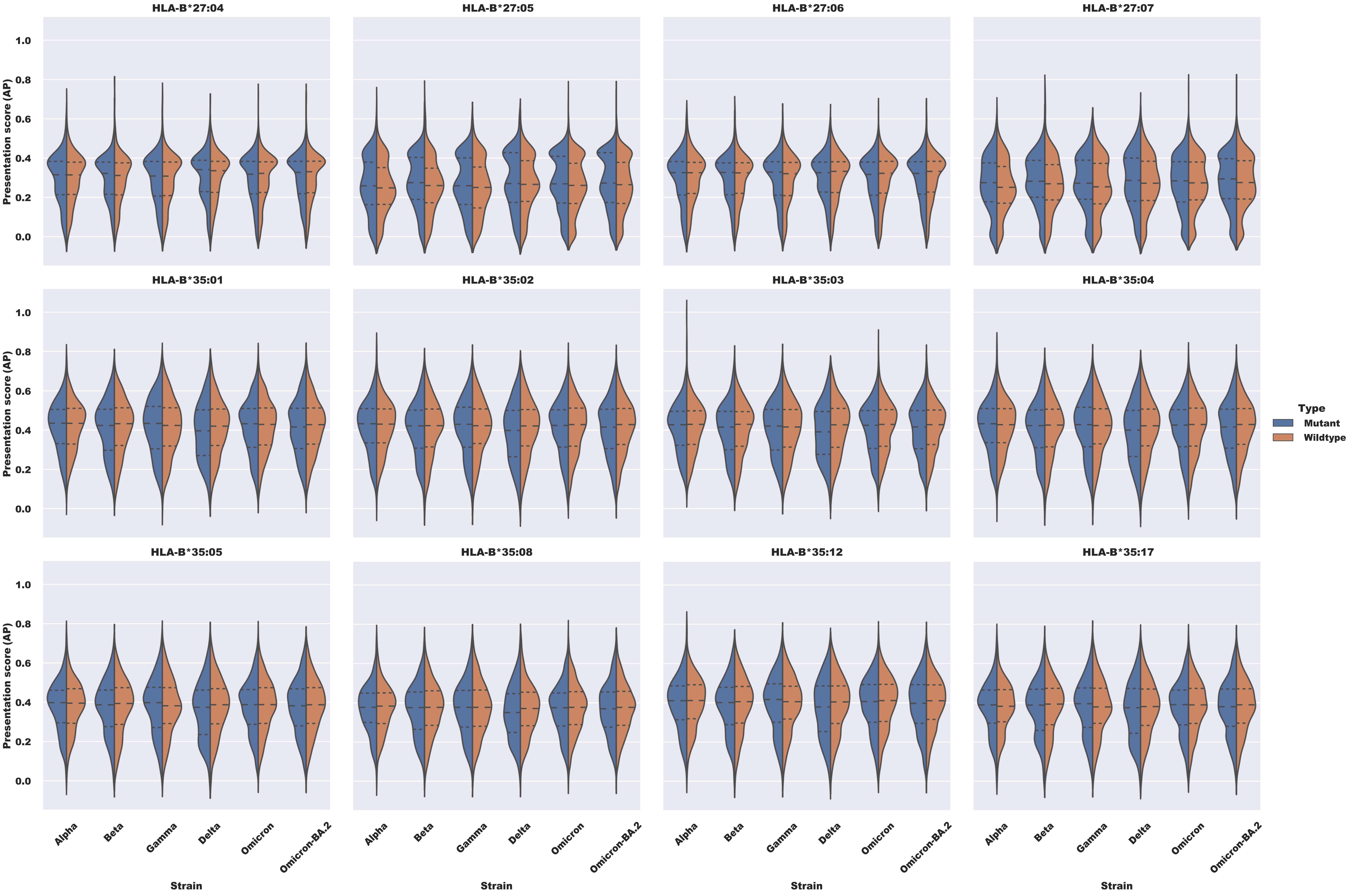

Supplementary Figure S4: Violin plots of the distribution of presentation scores (AP) for mutant and wildtype epitopes per allele for each VOC and Omicron-BA.2. The dashed lines within each violin represent the quartiles.

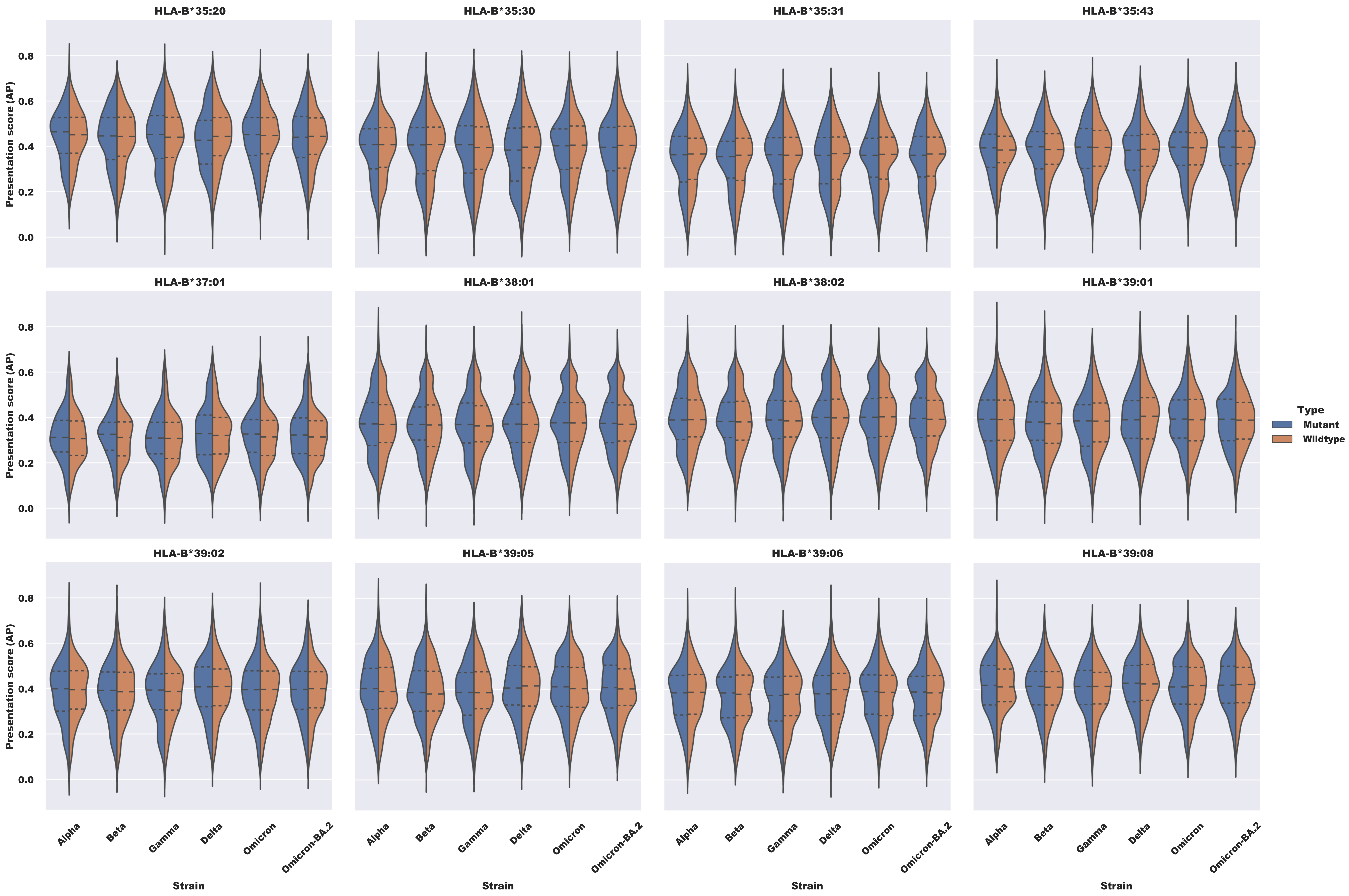

Supplementary Figure S4: Violin plots of the distribution of presentation scores (AP) for mutant and wildtype epitopes per allele for each VOC and Omicron-BA.2. The dashed lines within each violin represent the quartiles.

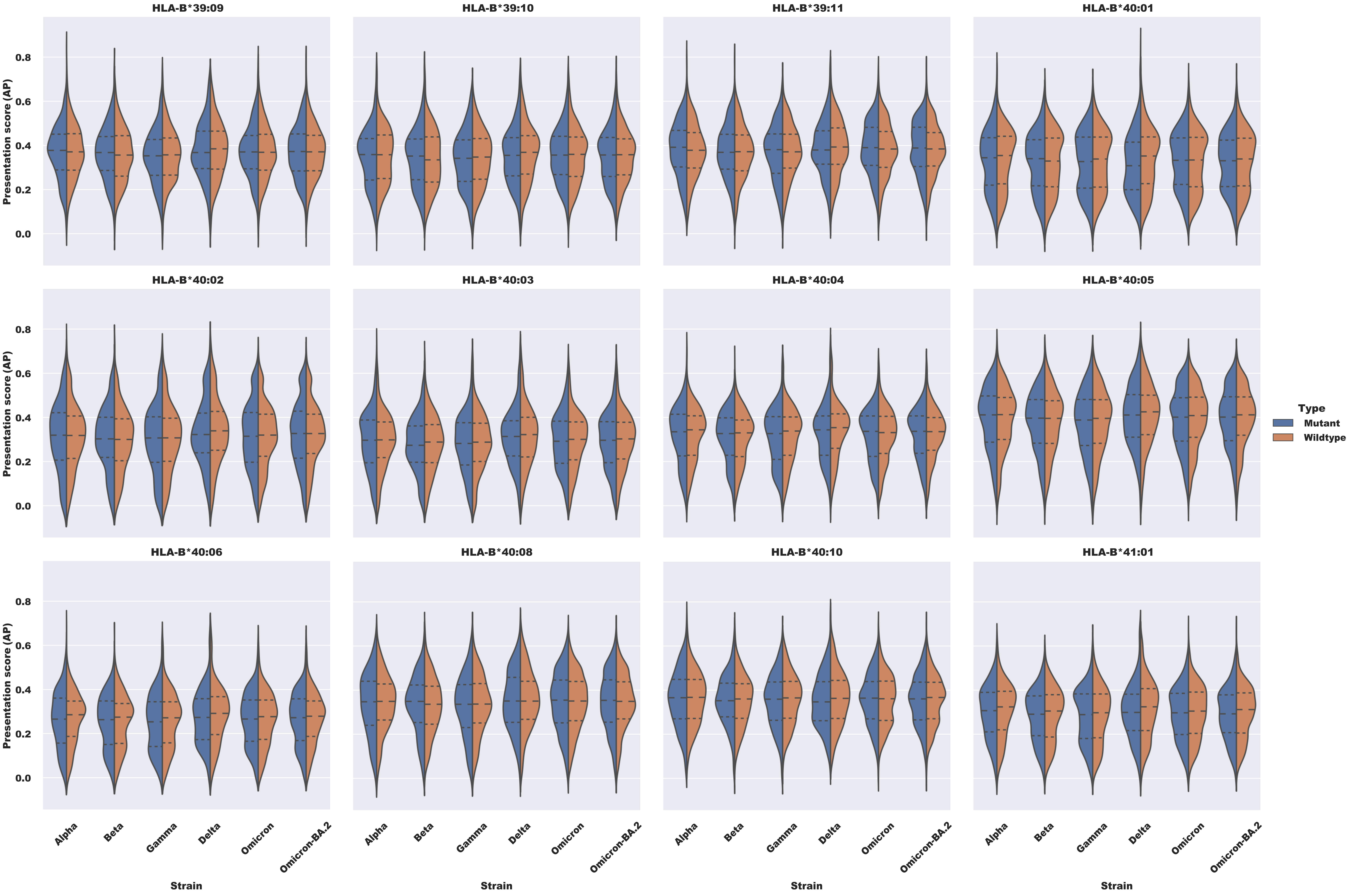

Supplementary Figure S4: Violin plots of the distribution of presentation scores (AP) for mutant and wildtype epitopes per allele for each VOC and Omicron-BA.2. The dashed lines within each violin represent the quartiles.

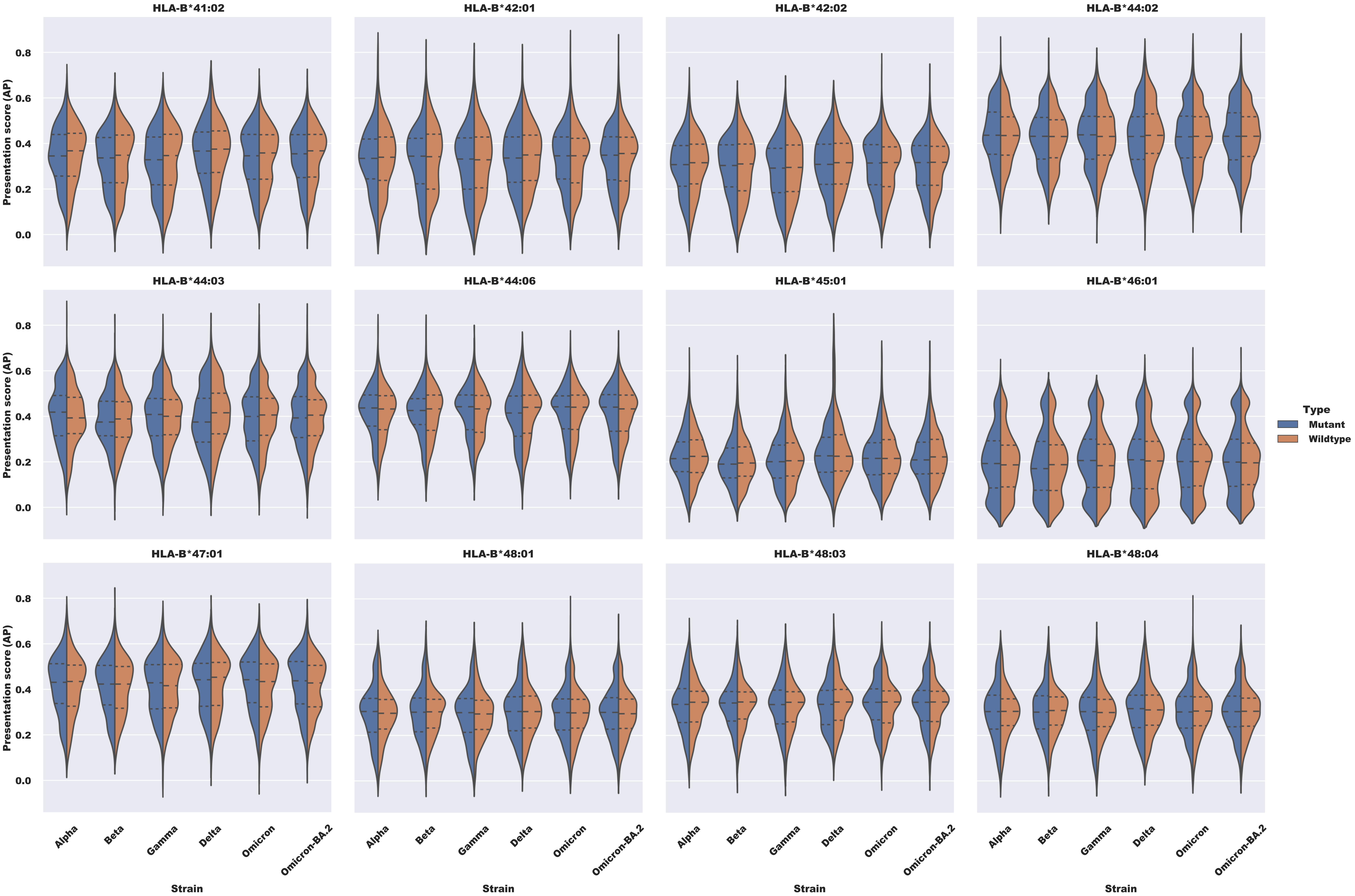

Supplementary Figure S4: Violin plots of the distribution of presentation scores (AP) for mutant and wildtype epitopes per allele for each VOC and Omicron-BA.2. The dashed lines within each violin represent the quartiles.

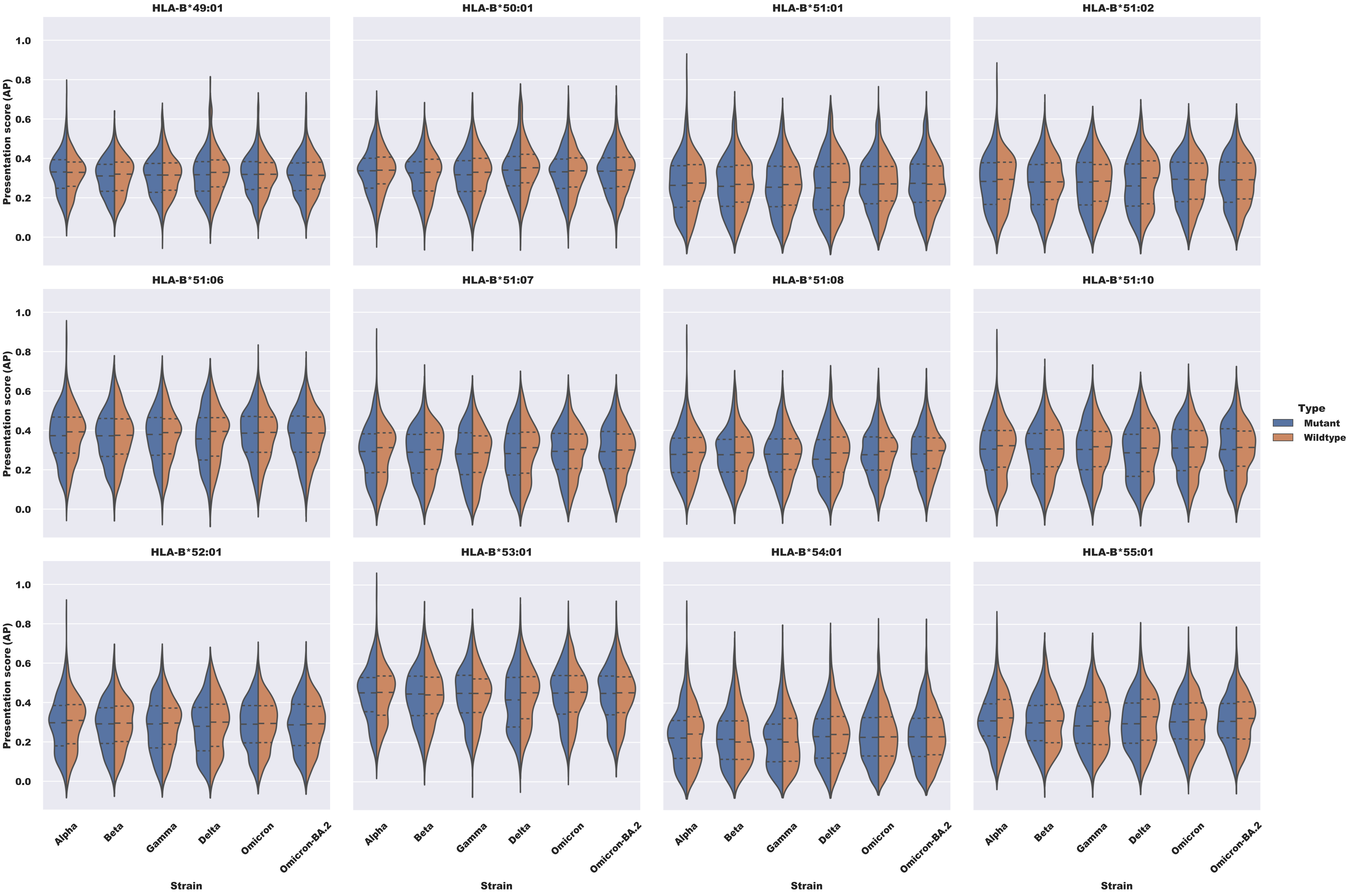

Supplementary Figure S4: Violin plots of the distribution of presentation scores (AP) for mutant and wildtype epitopes per allele for each VOC and Omicron-BA.2. The dashed lines within each violin represent the quartiles.

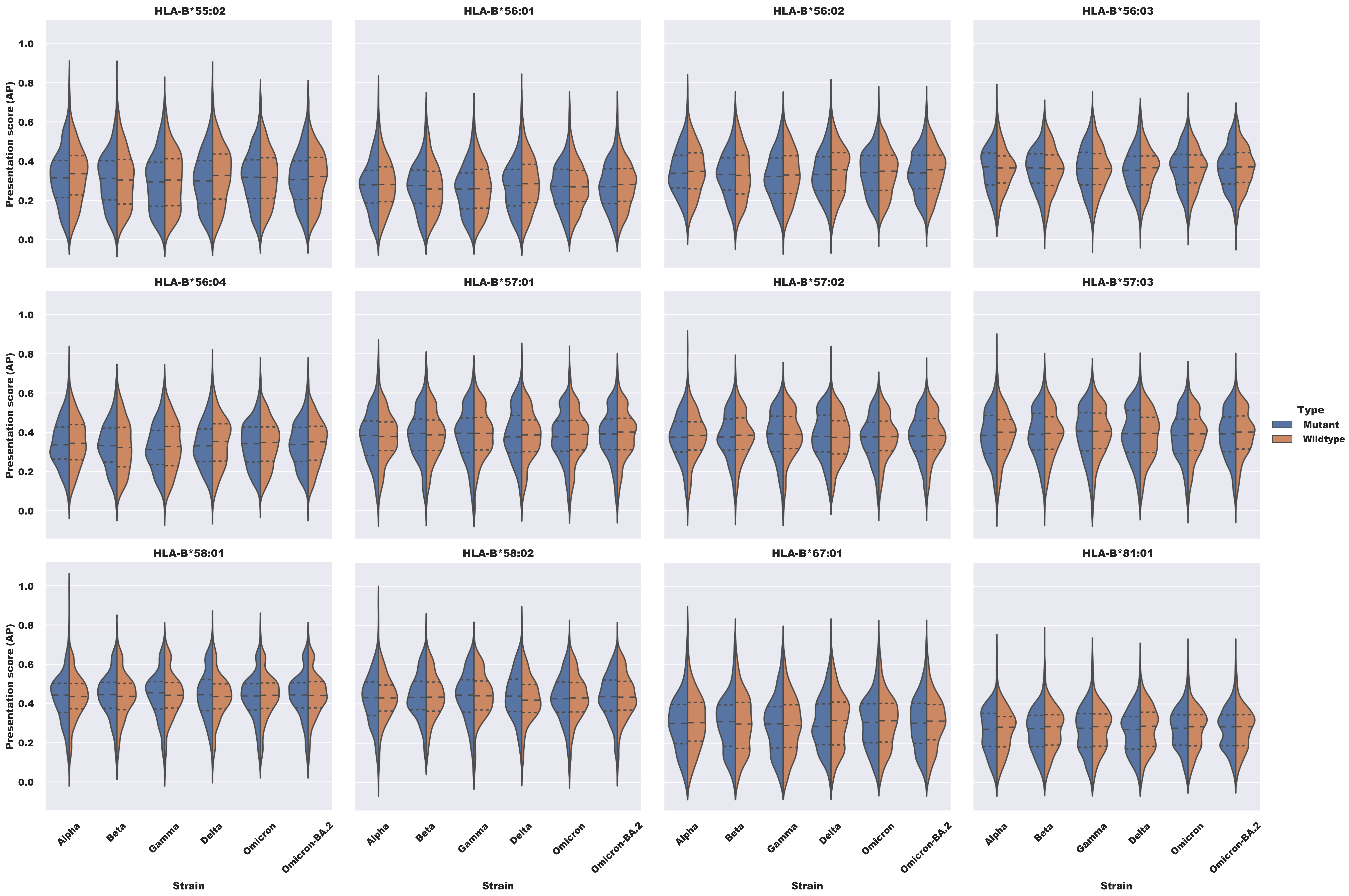

Supplementary Figure S5: Density of average AP scores > 0.5 at each mutation for a mutant (foreground) and wildtype (background) − protein E

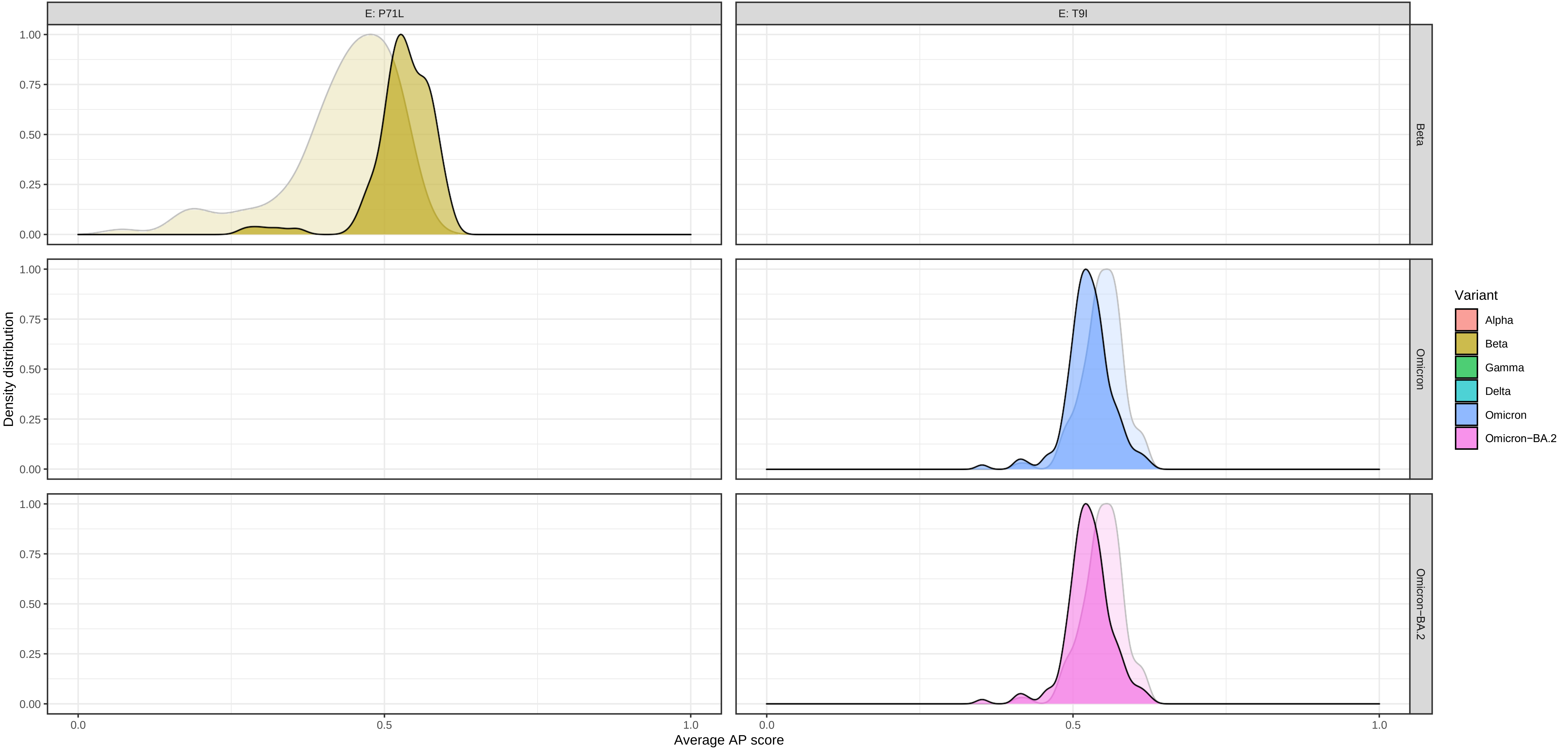

Supplementary Figure S5: Density of average AP scores > 0.5 at each mutation for a mutant (foreground) and wildtype (background) − protein M

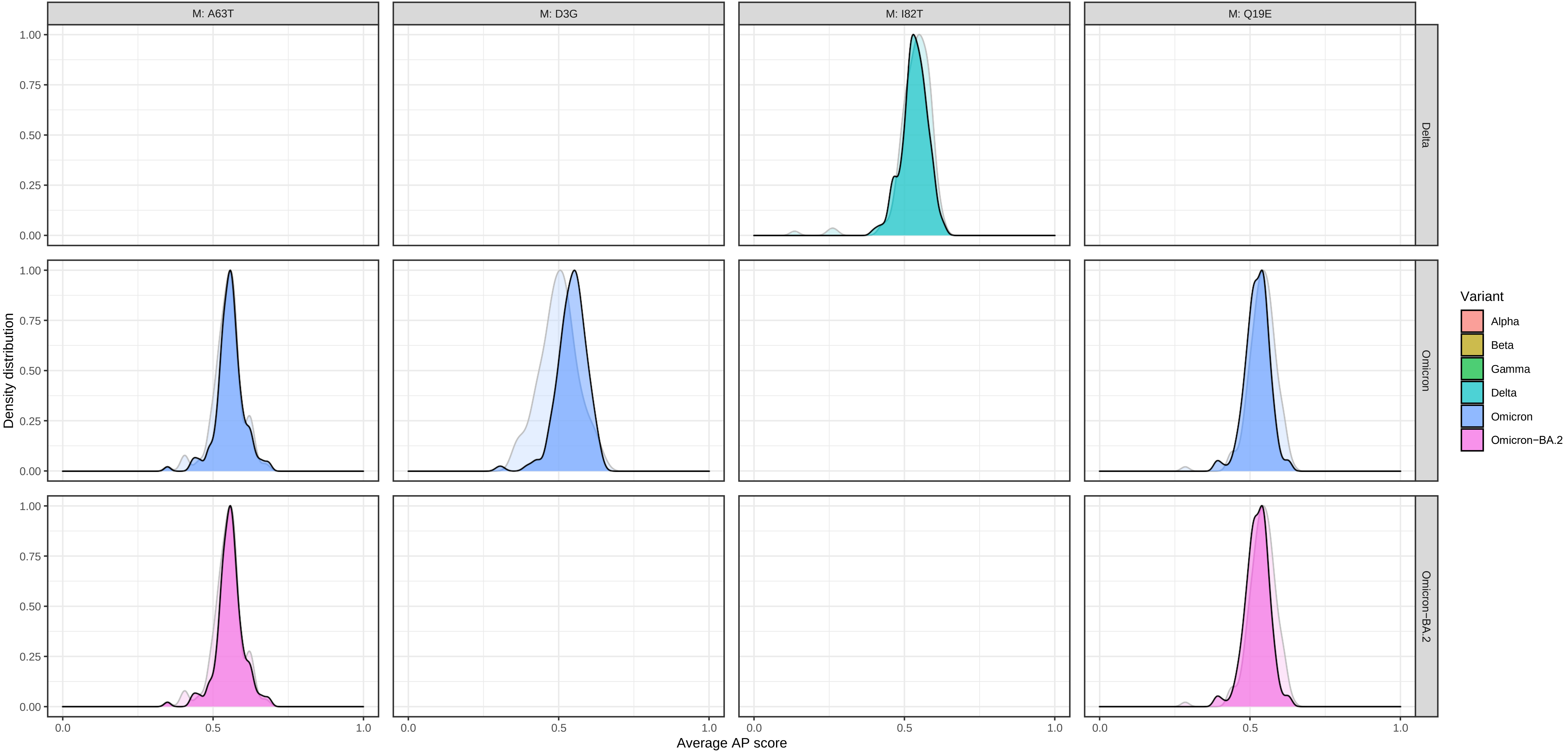

Supplementary Figure S5: Density of average AP scores > 0.5 at each mutation for a mutant (foreground) and wildtype (background) − protein

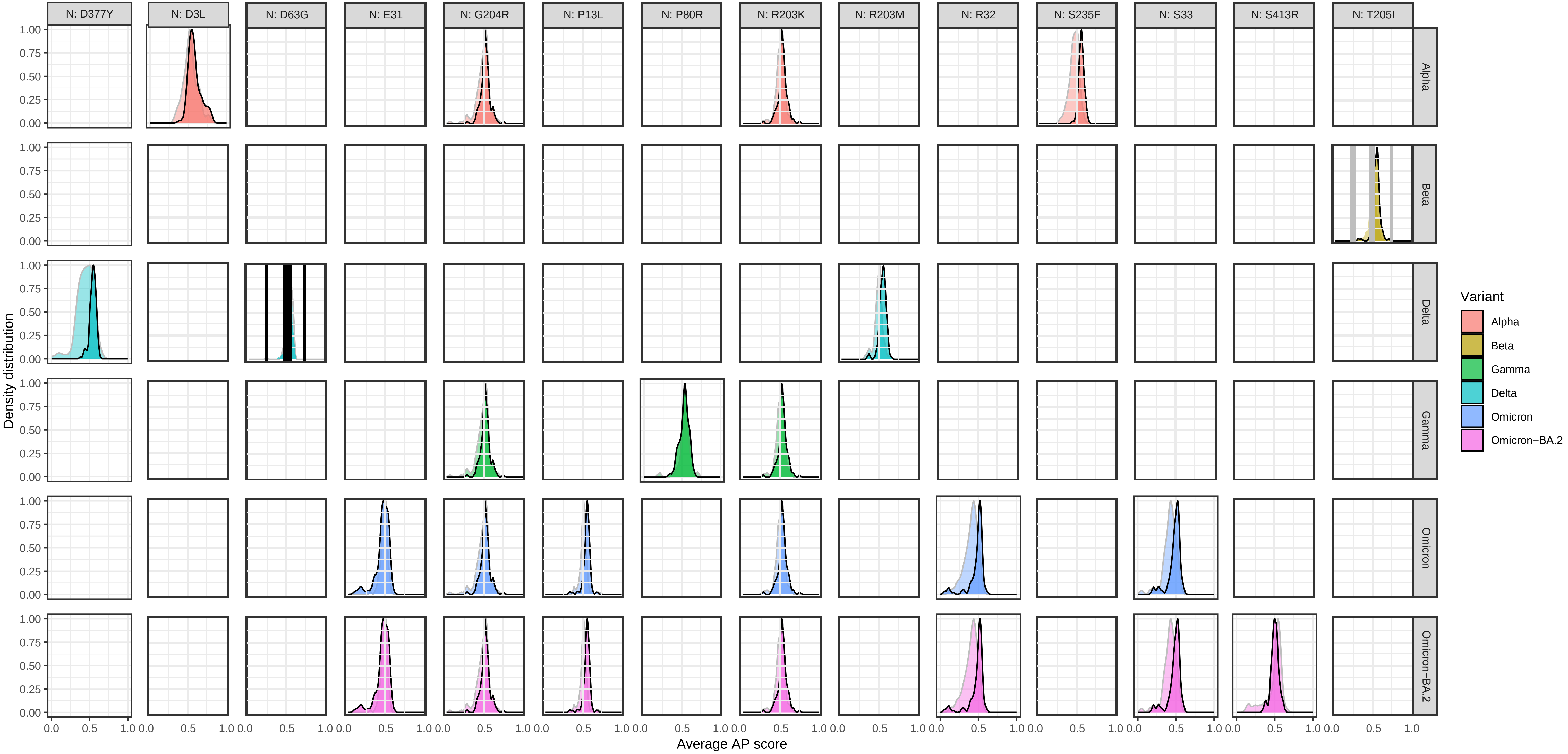

Supplementary Figure S5: Density of average AP scores > 0.5 at each mutation for a mutant (foreground) and wildtype (background) − protein

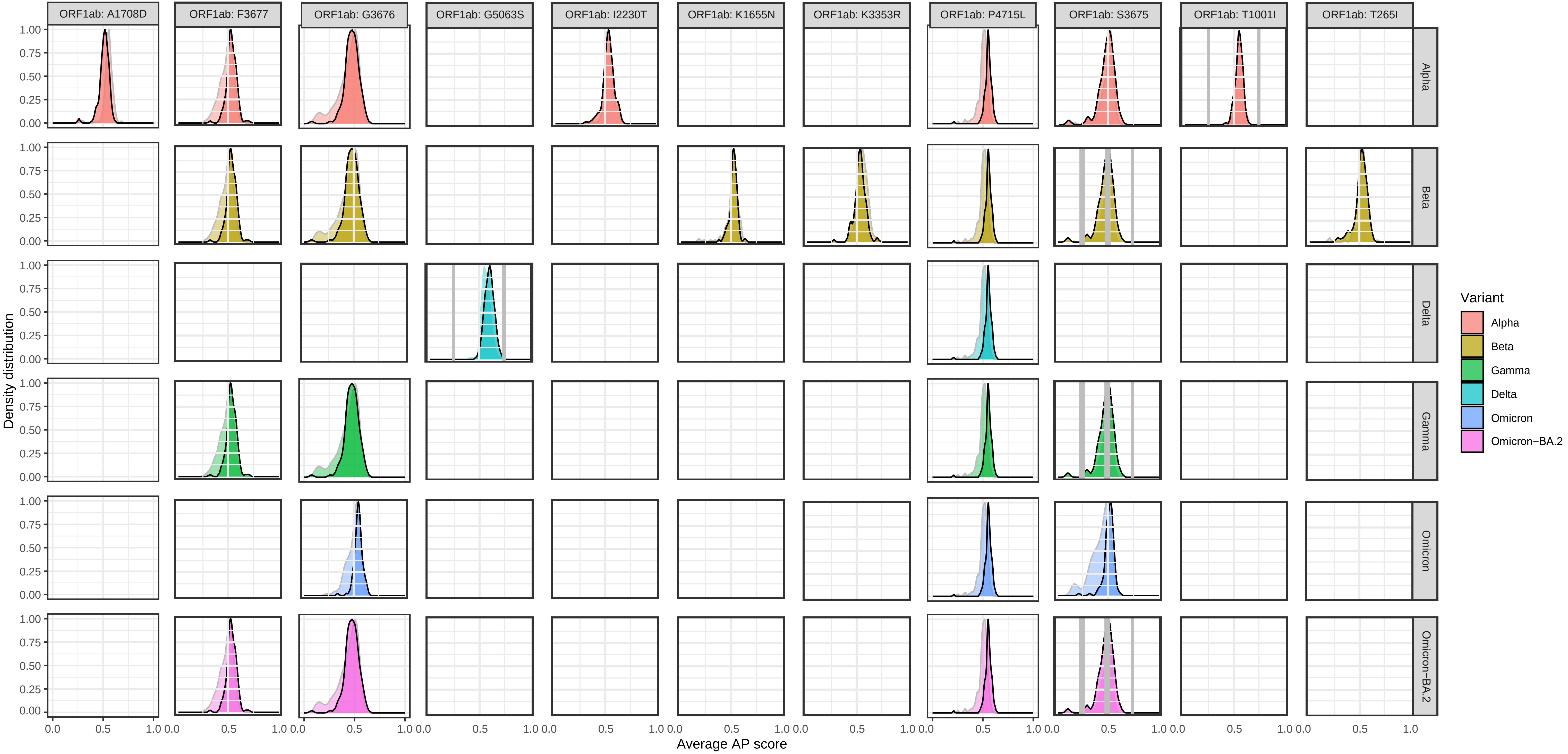

Supplementary Figure S5: Density of average AP scores > 0.5 at each mutation for a mutant (foreground) and wildtype (background) − protein

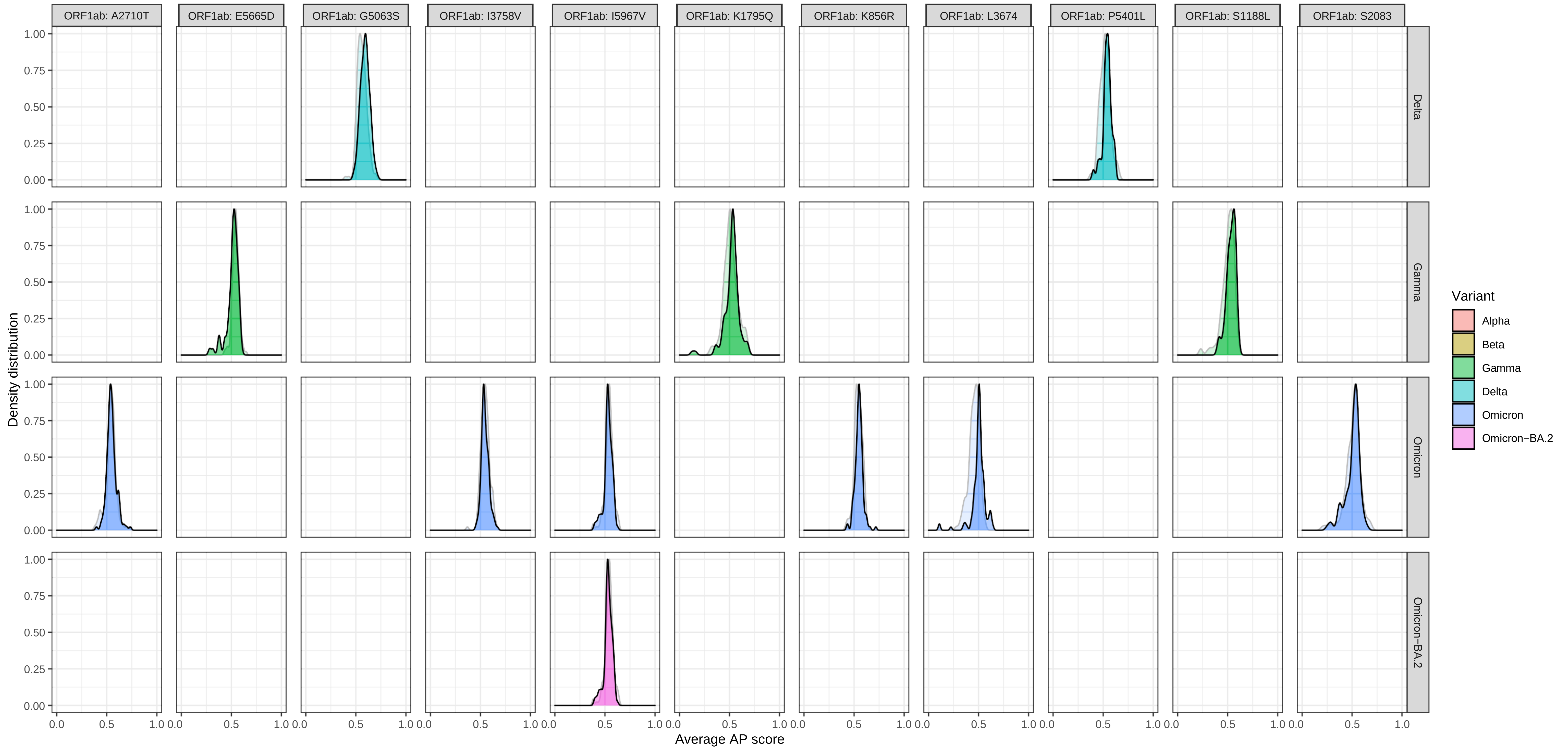

Supplementary Figure S5: Density of average AP scores > 0.5 at each mutation for a mutant (foreground) and wildtype (background) − protein

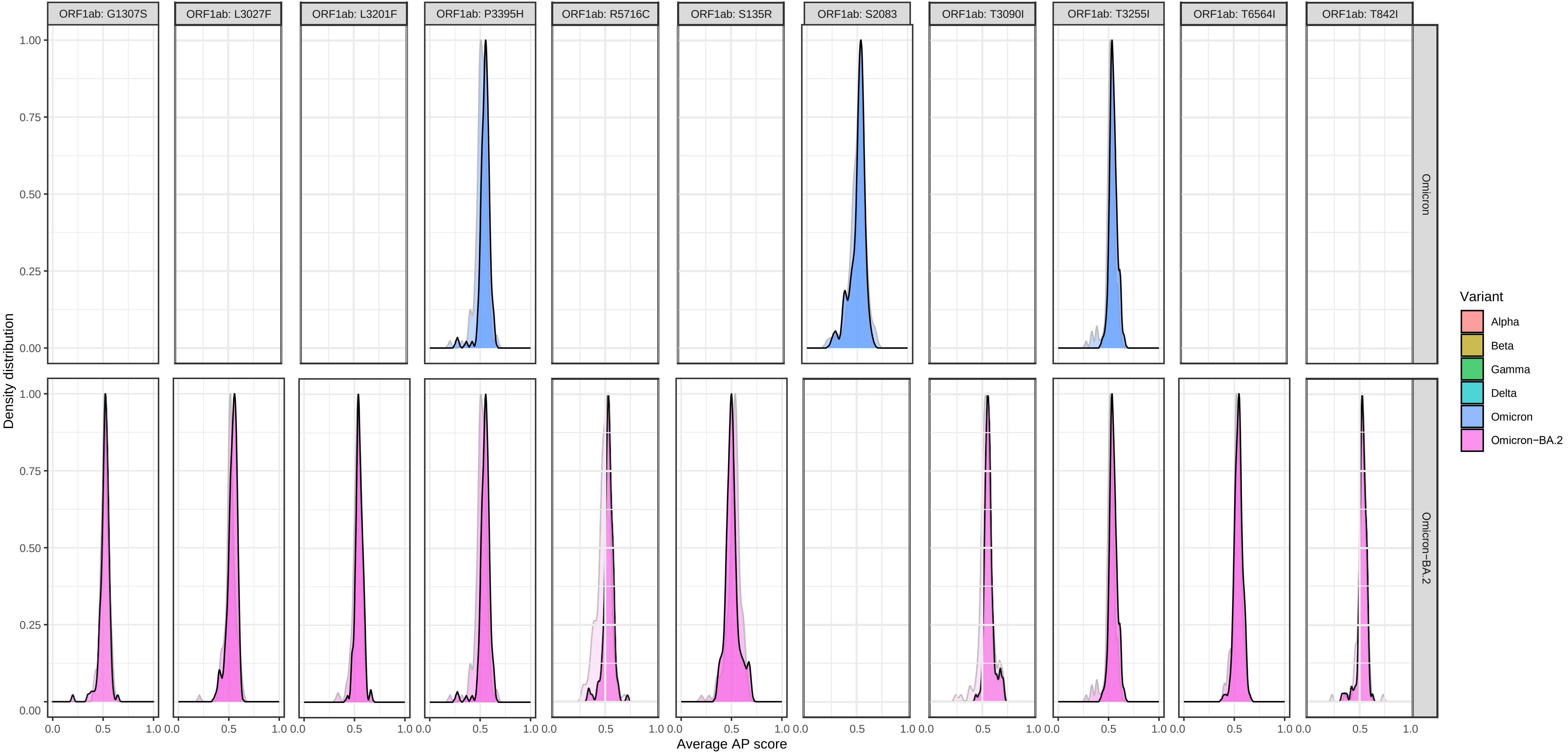

Supplementary Figure S5: Density of average AP scores > 0.5 at each mutation for a mutant (foreground) and wildtype (background) − protein

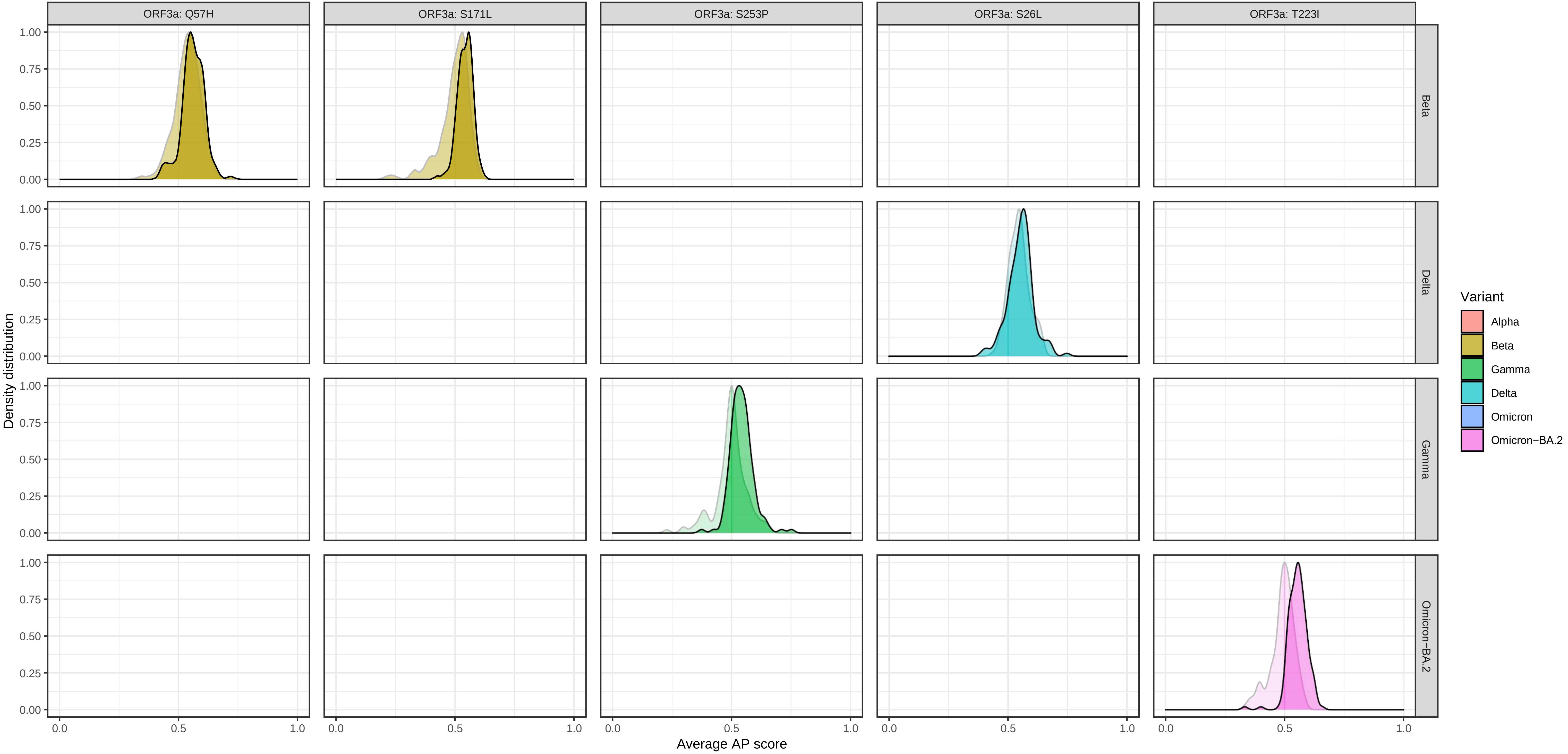

Supplementary Figure S5: Density of average AP scores > 0.5 at each mutation for a mutant (foreground) and wildtype (background) − protein

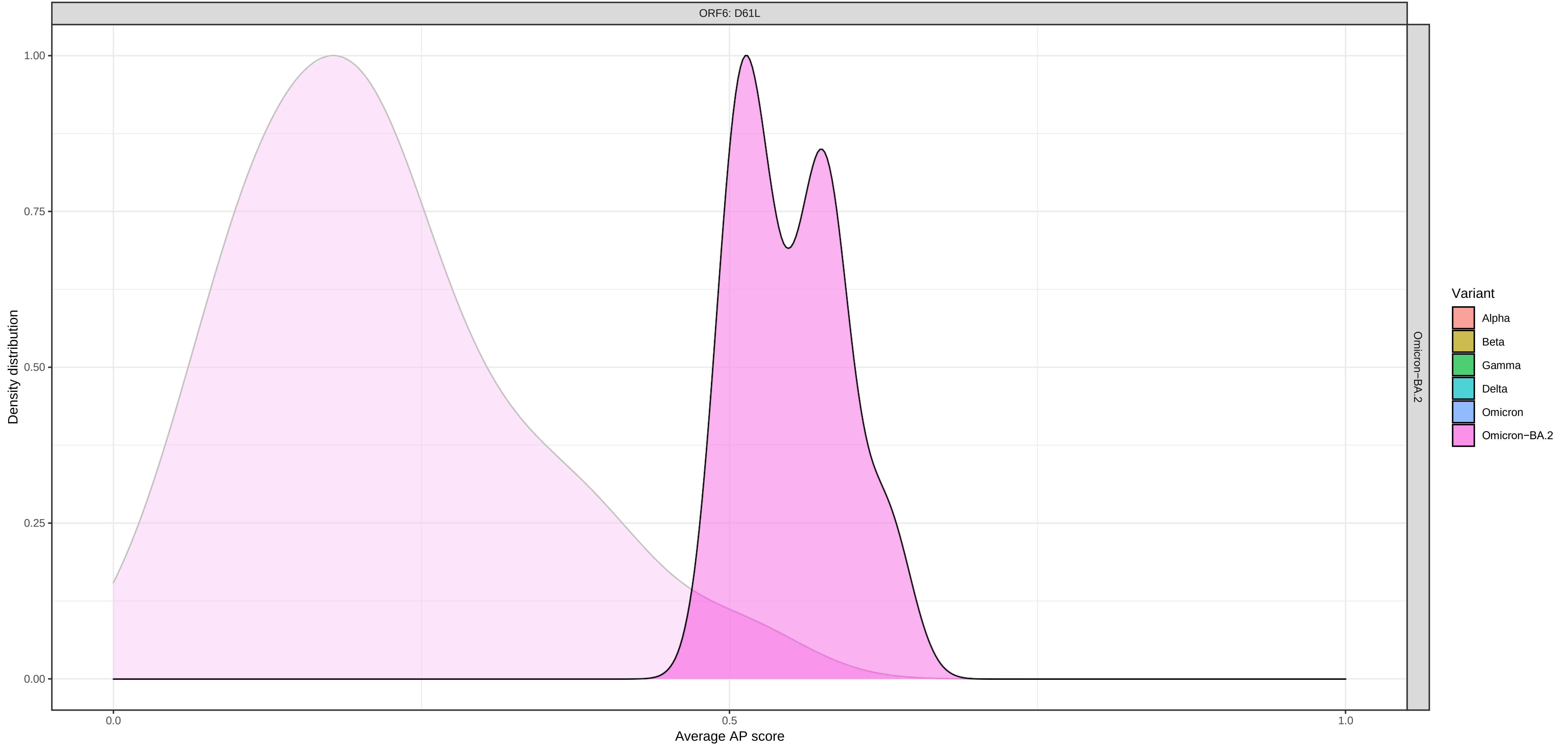

Supplementary Figure S5: Density of average AP scores > 0.5 at each mutation for a mutant (foreground) and wildtype (background) − protein

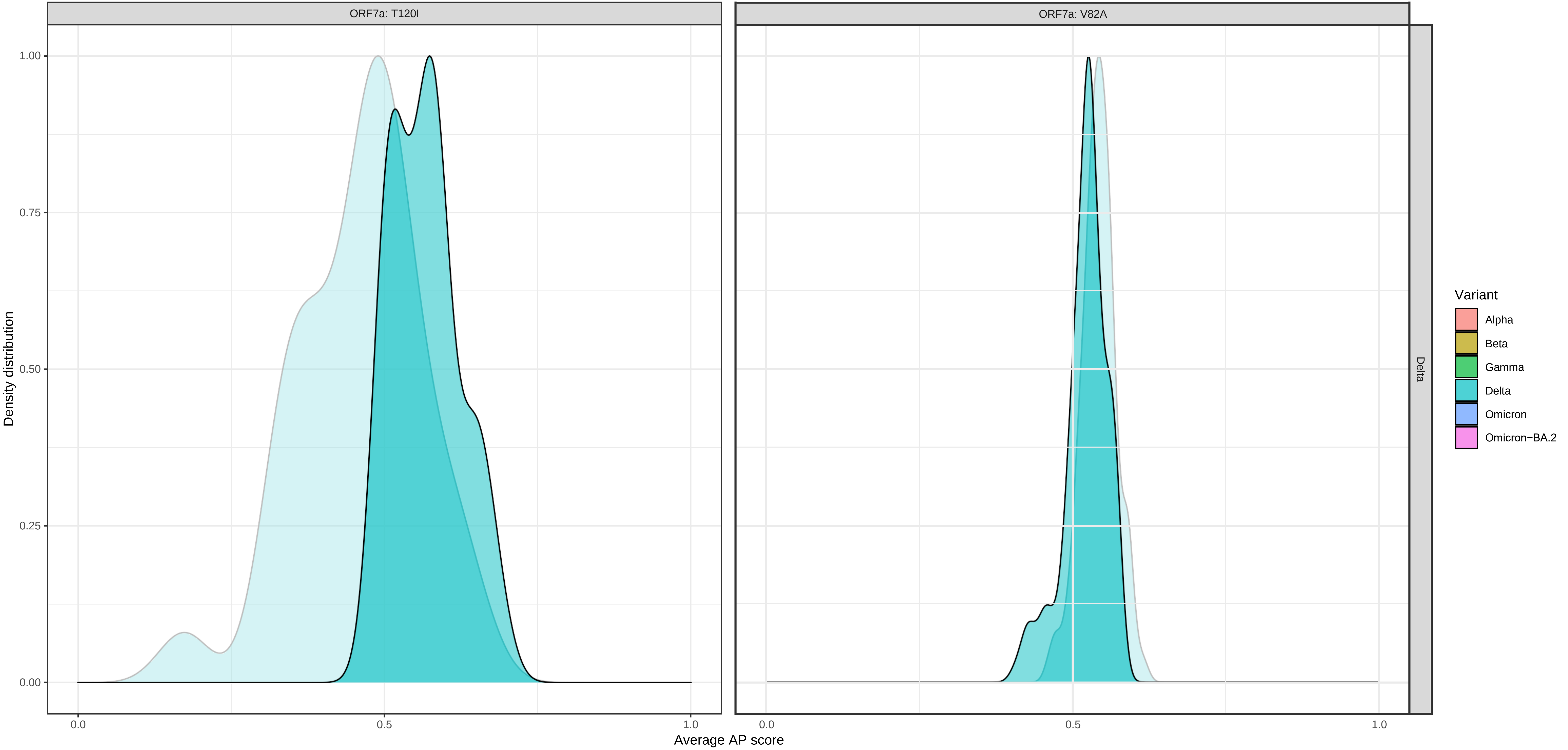

Supplementary Figure S5: Density of average AP scores > 0.5 at each mutation for a mutant (foreground) and wildtype (background) − protein

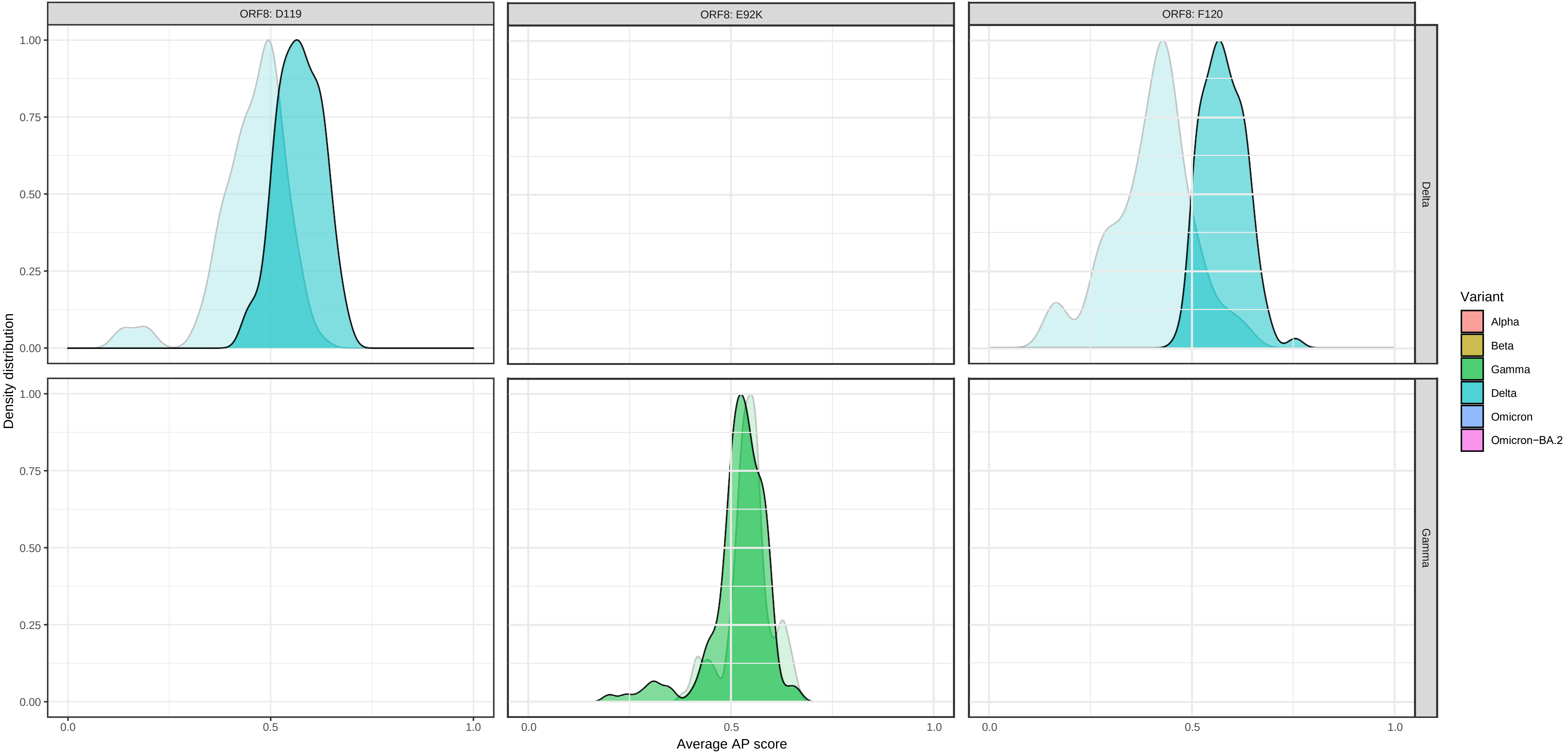

Supplementary Figure S5: Density of average AP scores > 0.5 at each mutation for a mutant (foreground) and wildtype (background) − protein

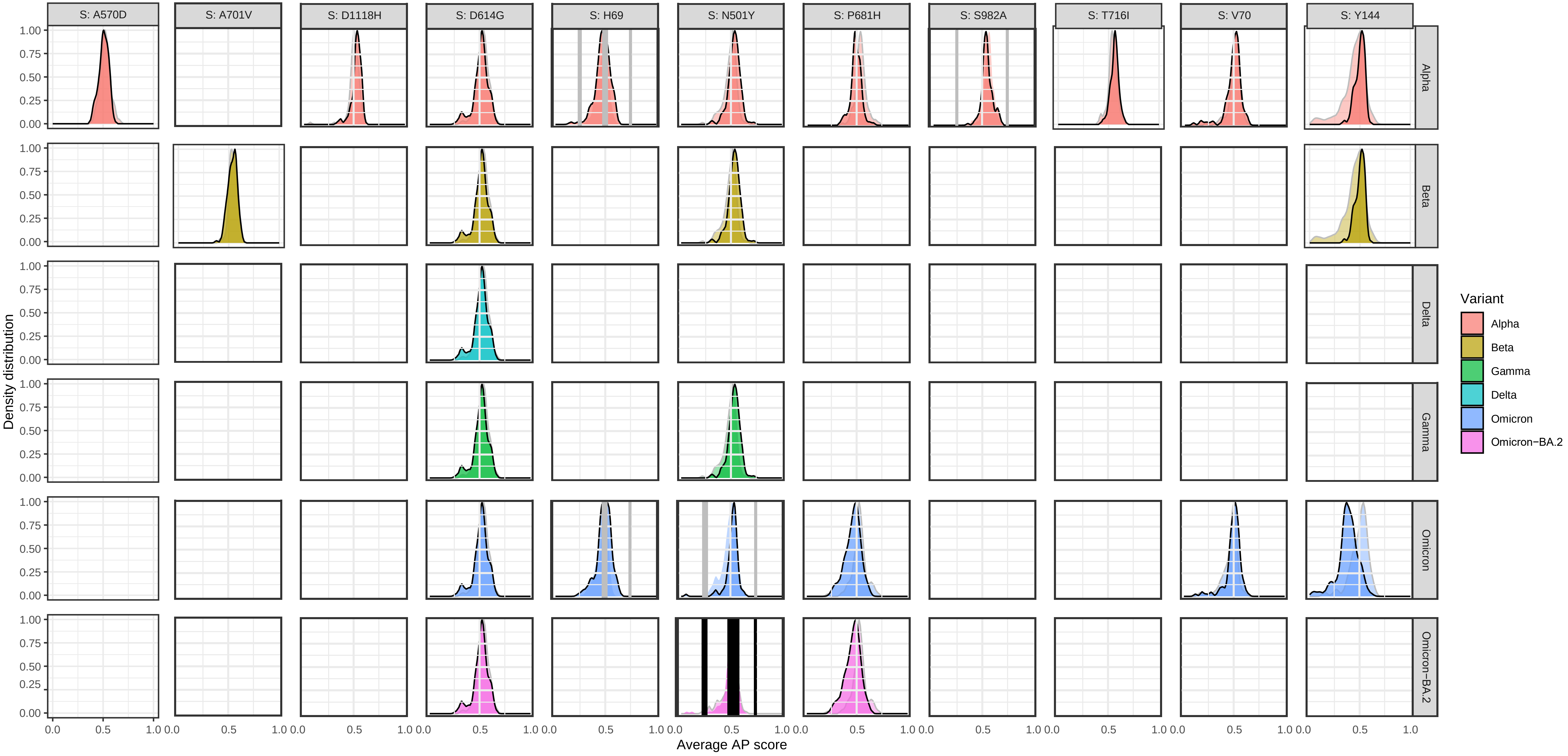

Supplementary Figure S5: Density of average AP scores > 0.5 at each mutation for a mutant (foreground) and wildtype (background) − protein

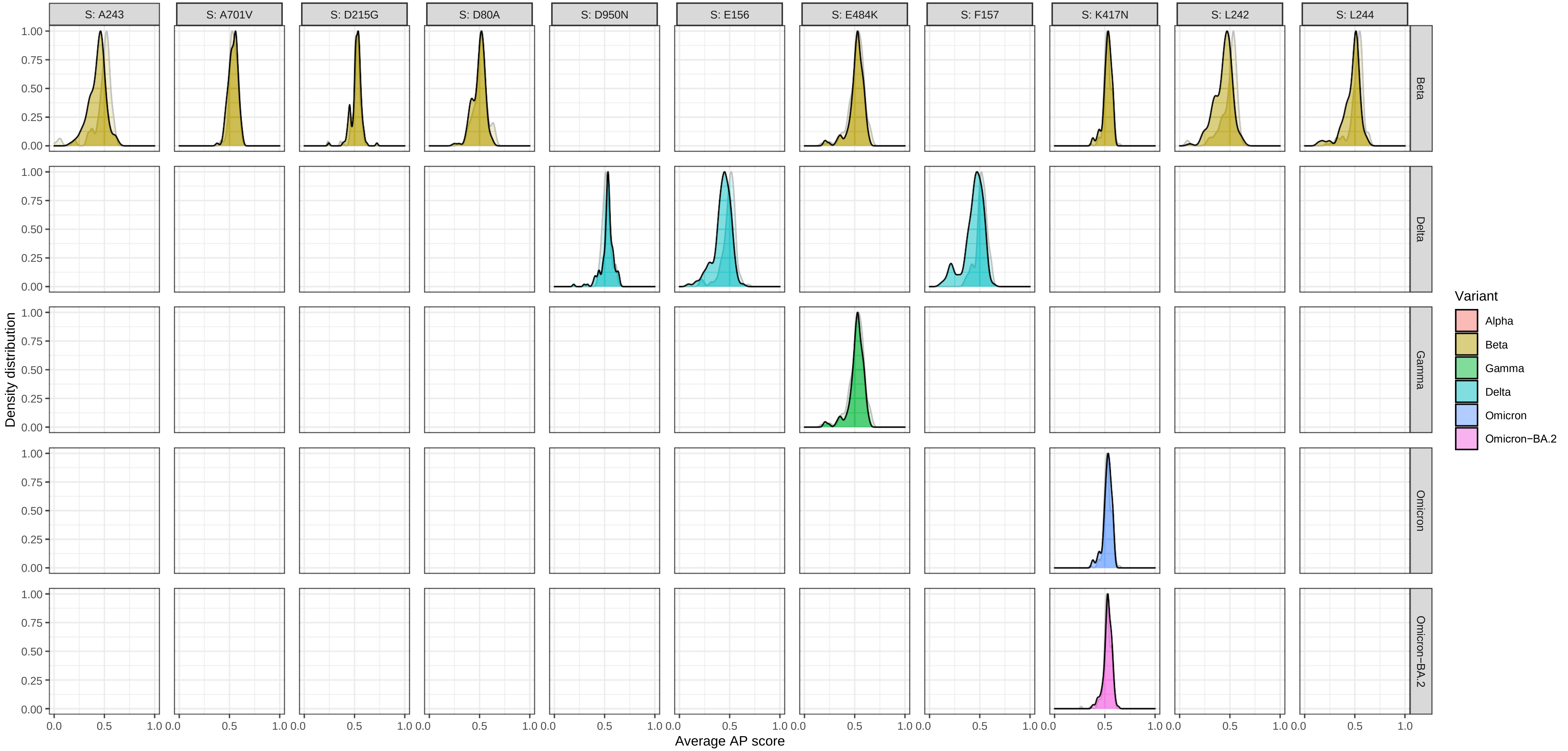

Supplementary Figure S5: Density of average AP scores > 0.5 at each mutation for a mutant (foreground) and wildtype (background) − protein

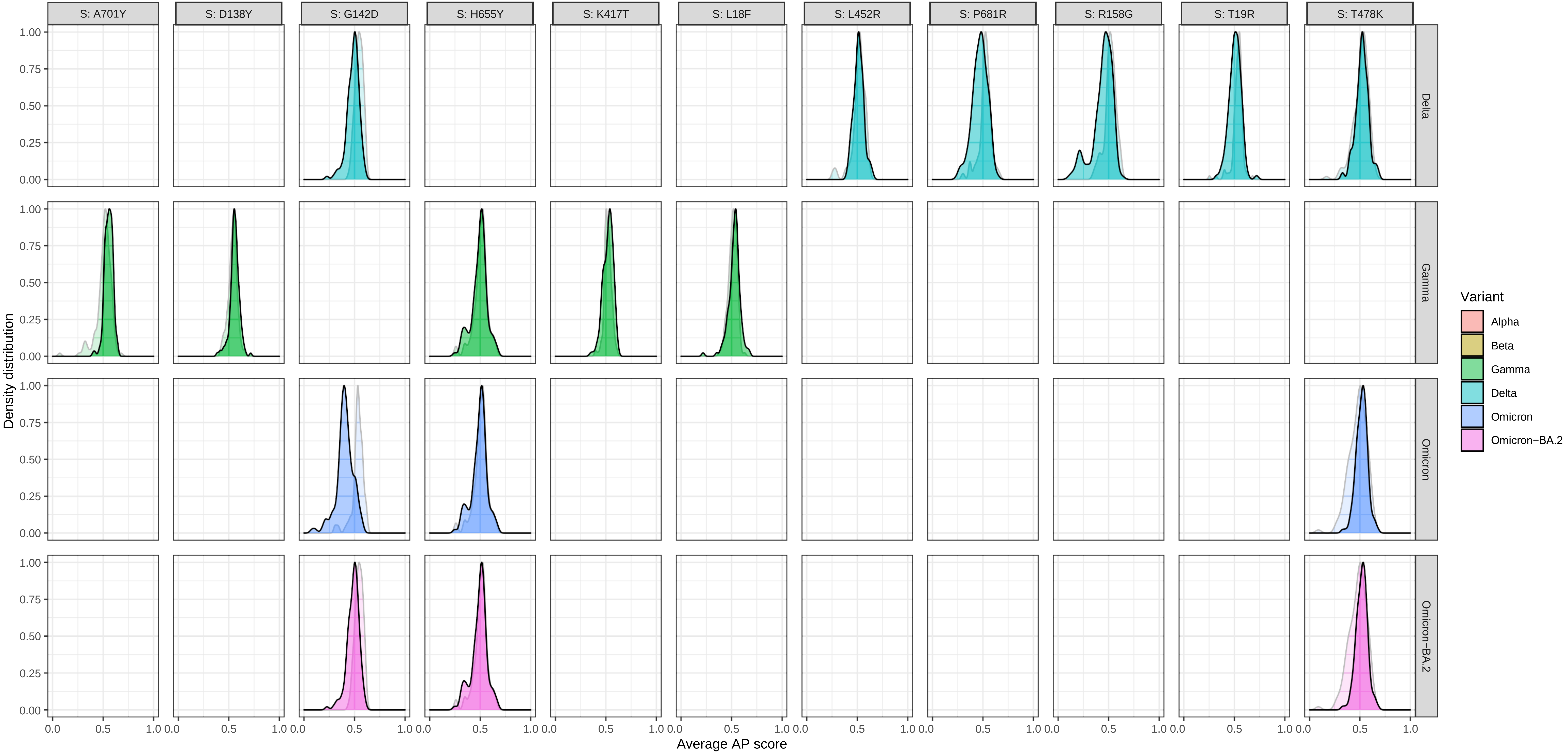

Supplementary Figure S5: Density of average AP scores > 0.5 at each mutation for a mutant (foreground) and wildtype (background) − protein

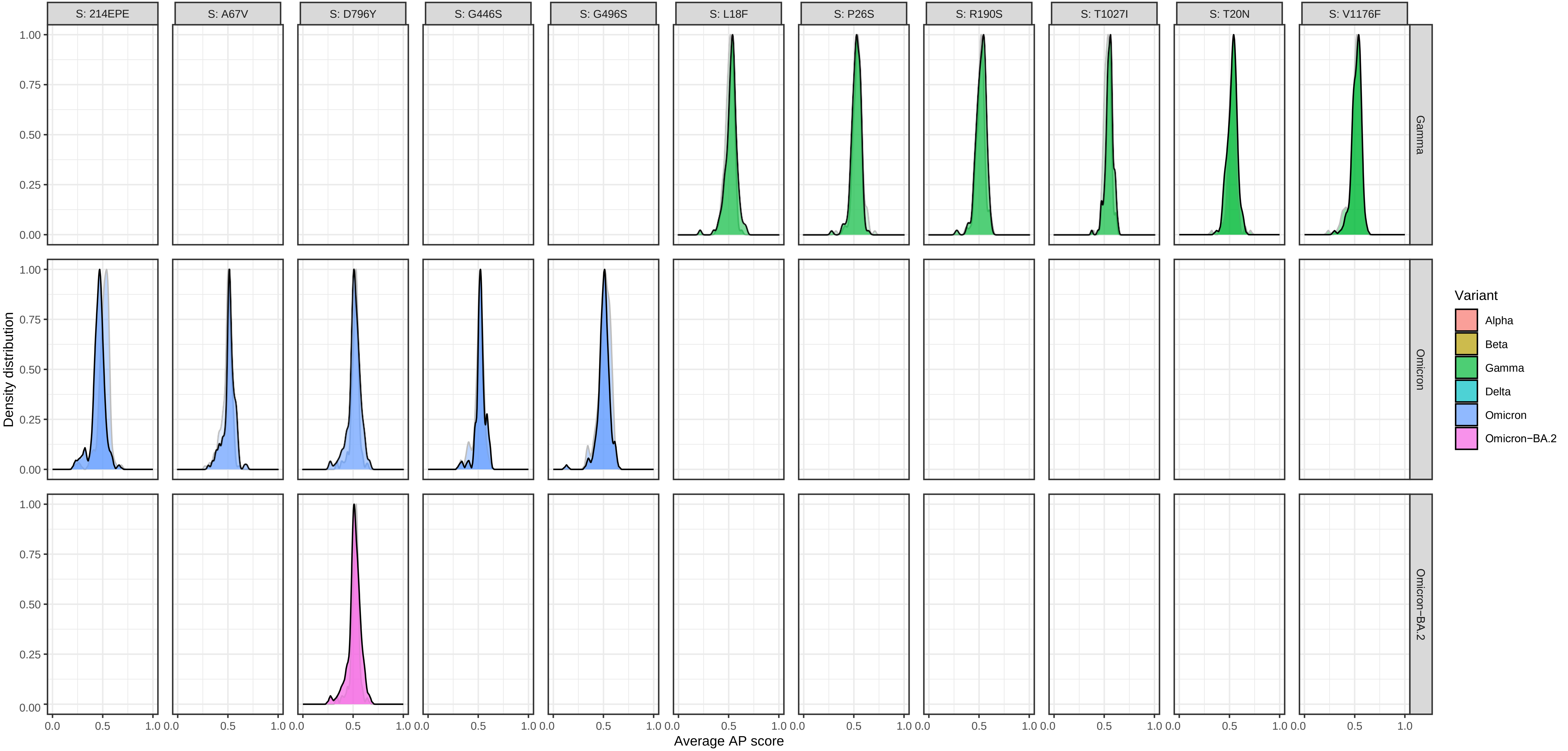

Supplementary Figure S5: Density of average AP scores > 0.5 at each mutation for a mutant (foreground) and wildtype (background) − protein

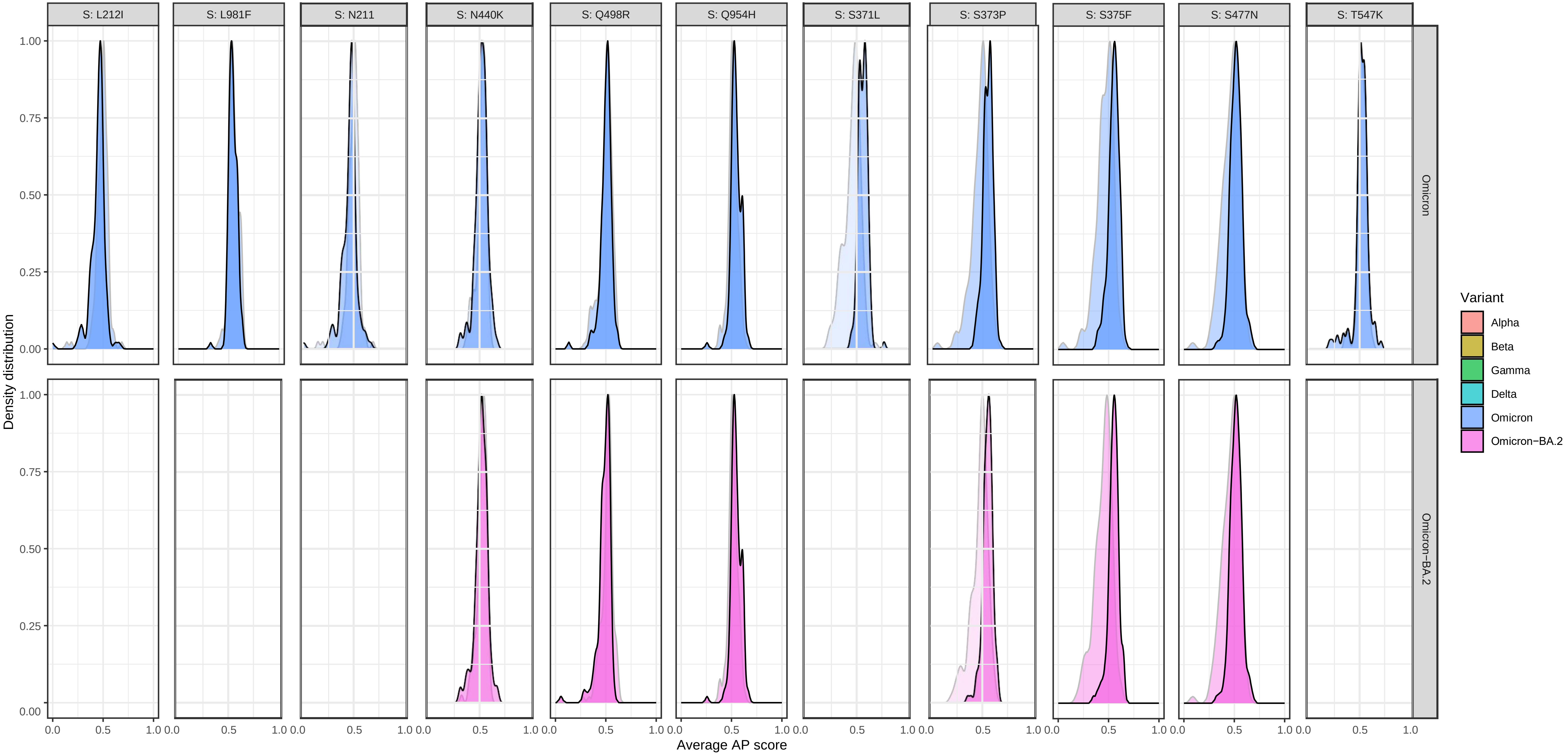

Supplementary Figure S5: Density of average AP scores > 0.5 at each mutation for a mutant (foreground) and wildtype (background) − protein

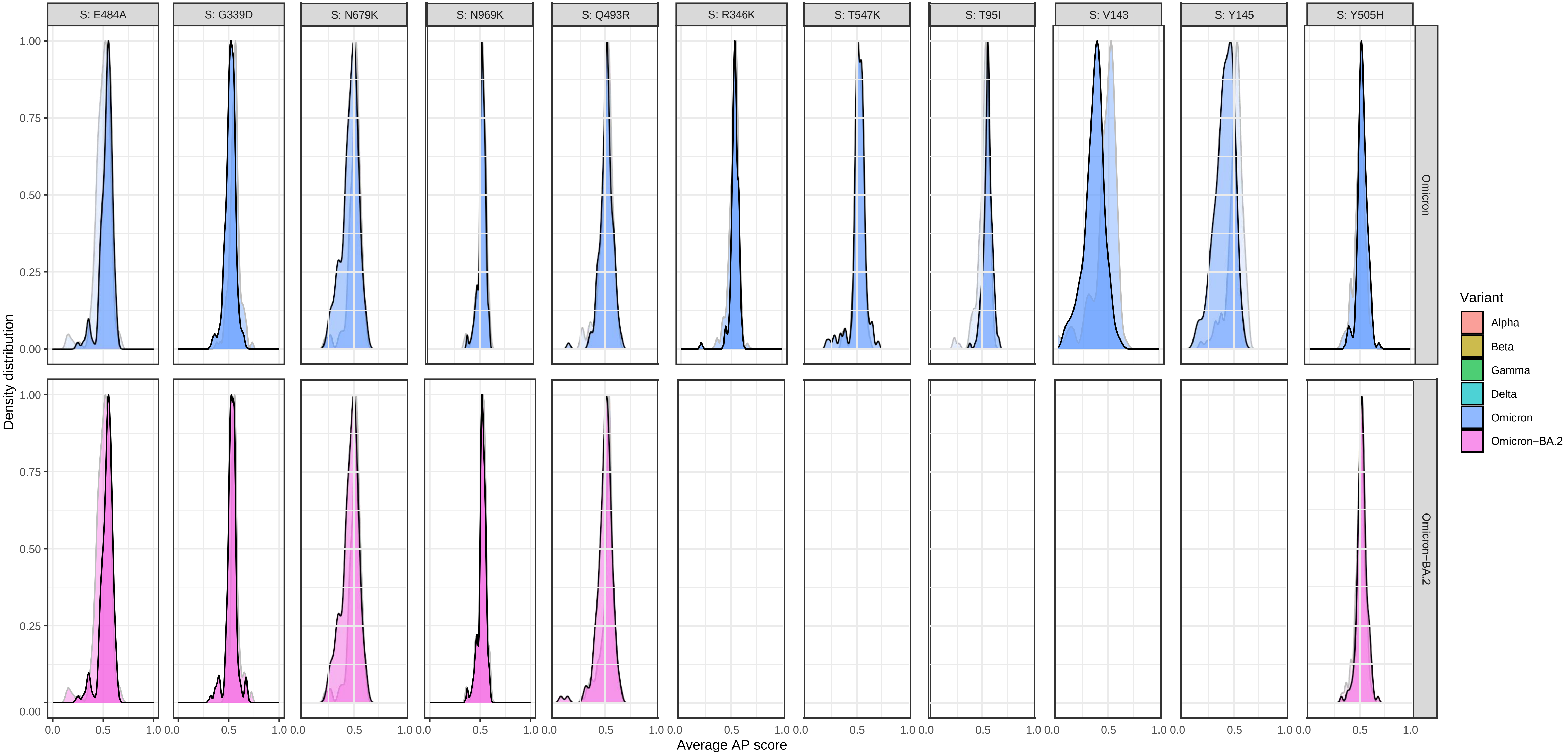

Supplementary Figure S5: Density of average AP scores > 0.5 at each mutation for a mutant (foreground) and wildtype (background) − protein

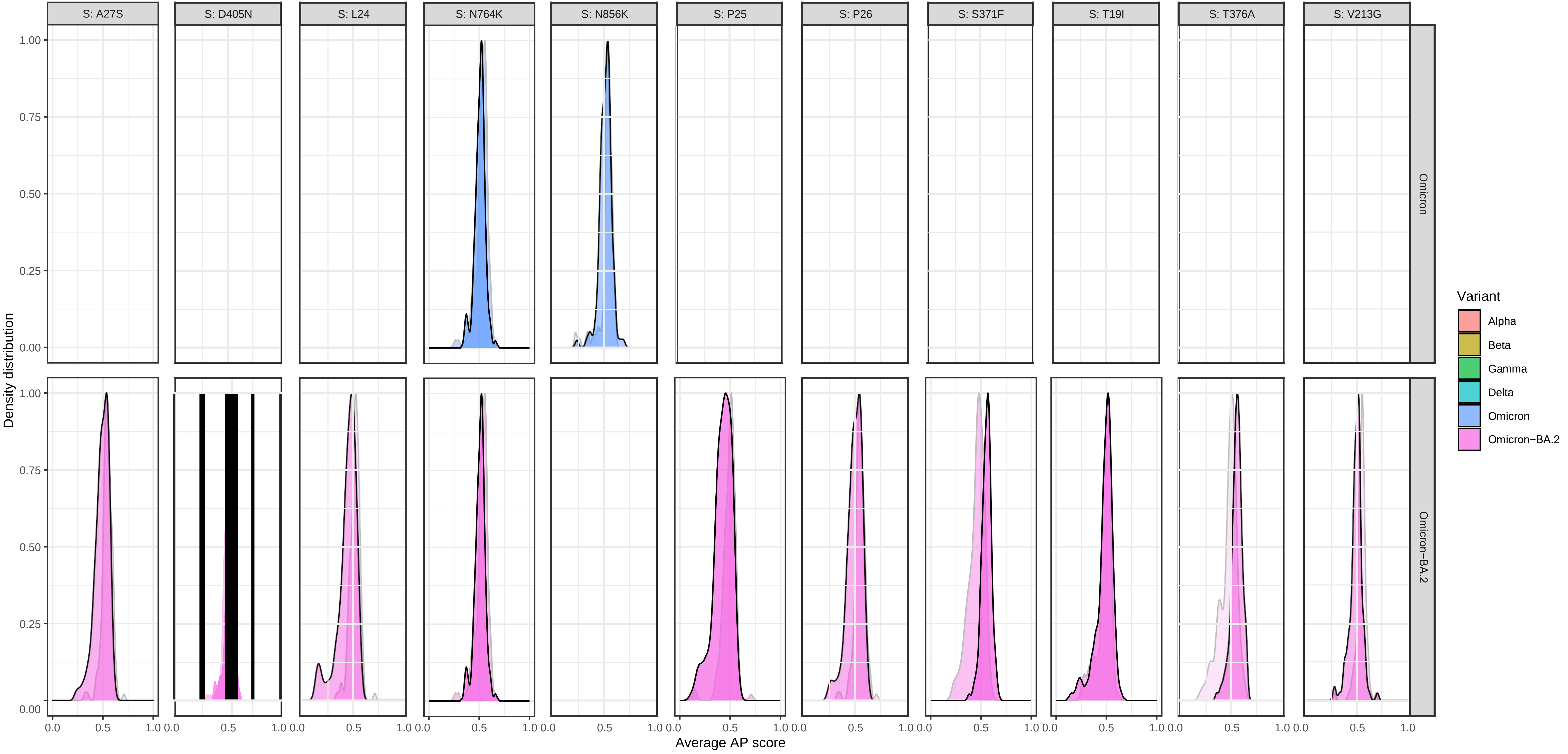

Supplementary Figure S6: Density of average AP scores at each mutation for a mutant (foreground) and wildtype (background) − protein

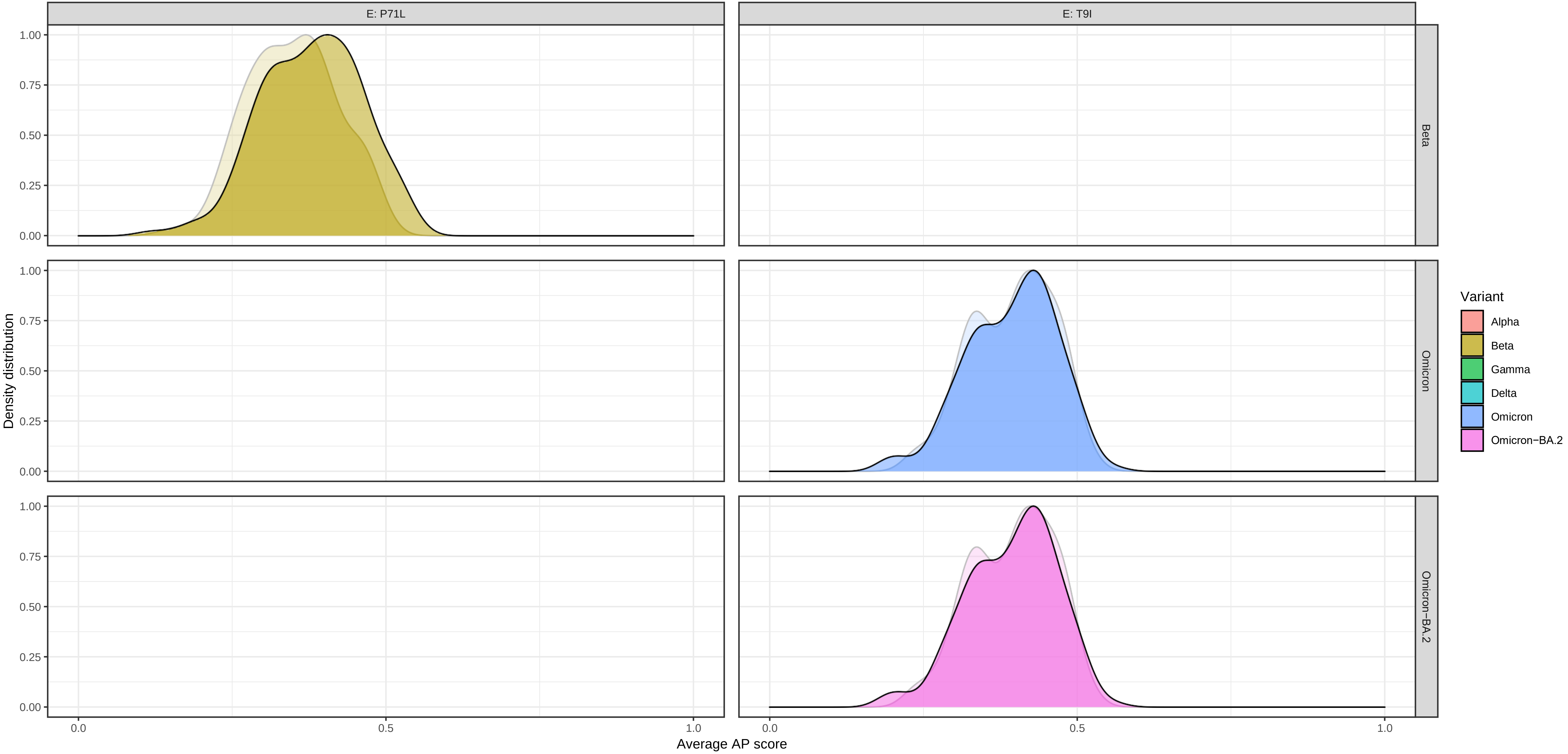

Supplementary Figure S6: Density of average AP scores at each mutation for a mutant (foreground) and wildtype (background) − protein

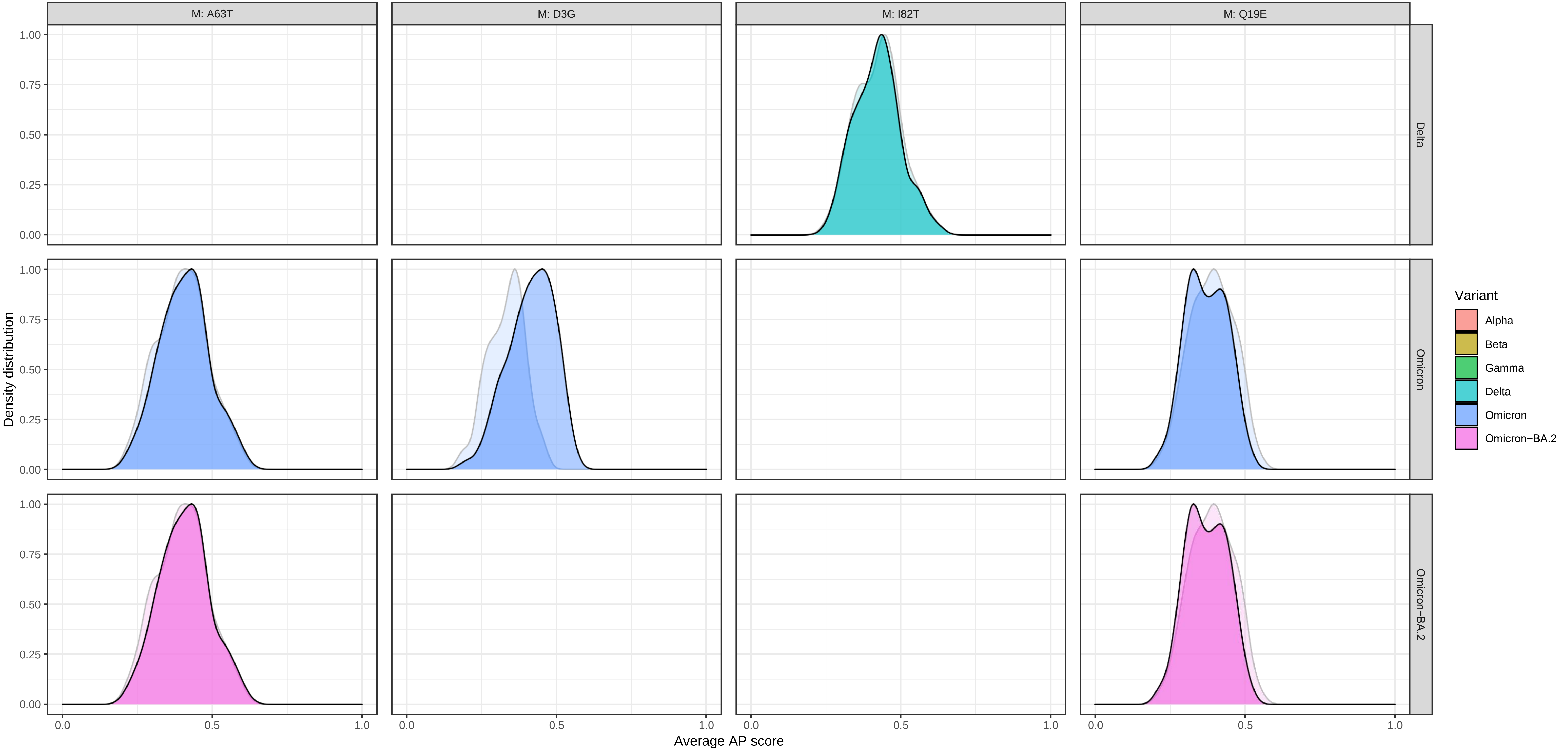

Supplementary Figure S6: Density of average AP scores at each mutation for a mutant (foreground) and wildtype (background) − protein

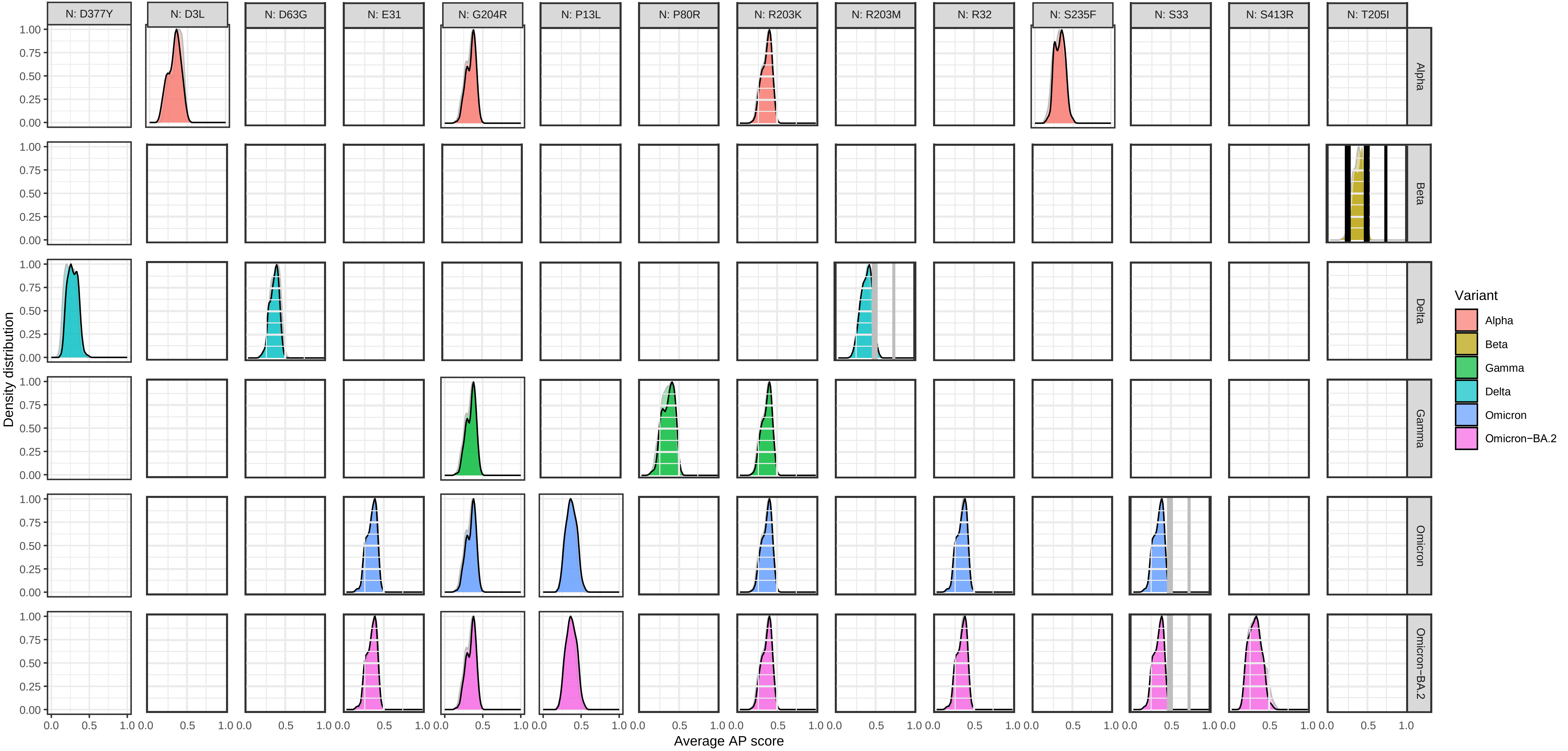

Supplementary Figure S6: Density of average AP scores at each mutation for a mutant (foreground) and wildtype (background) − protein

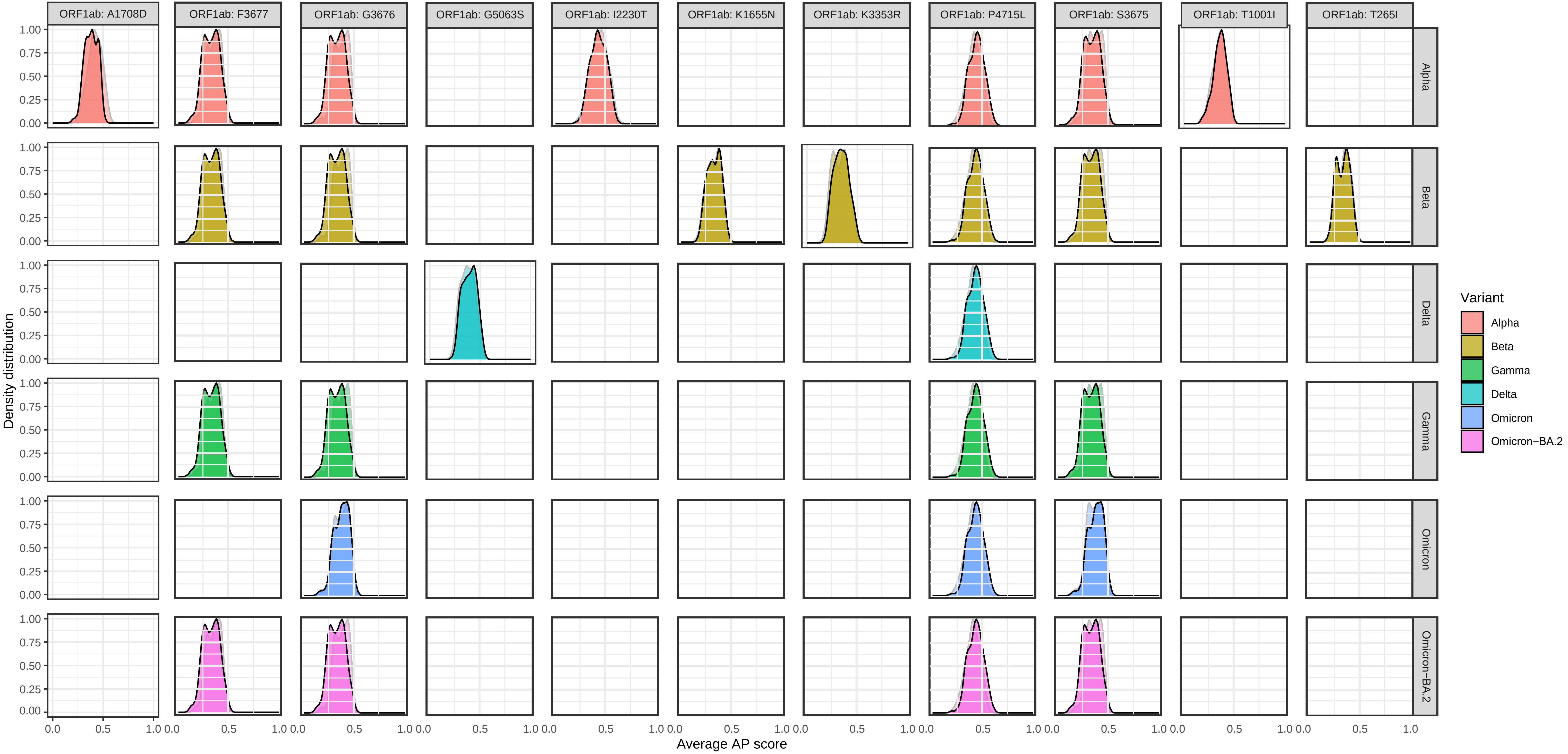

Supplementary Figure S6: Density of average AP scores at each mutation for a mutant (foreground) and wildtype (background) − protein

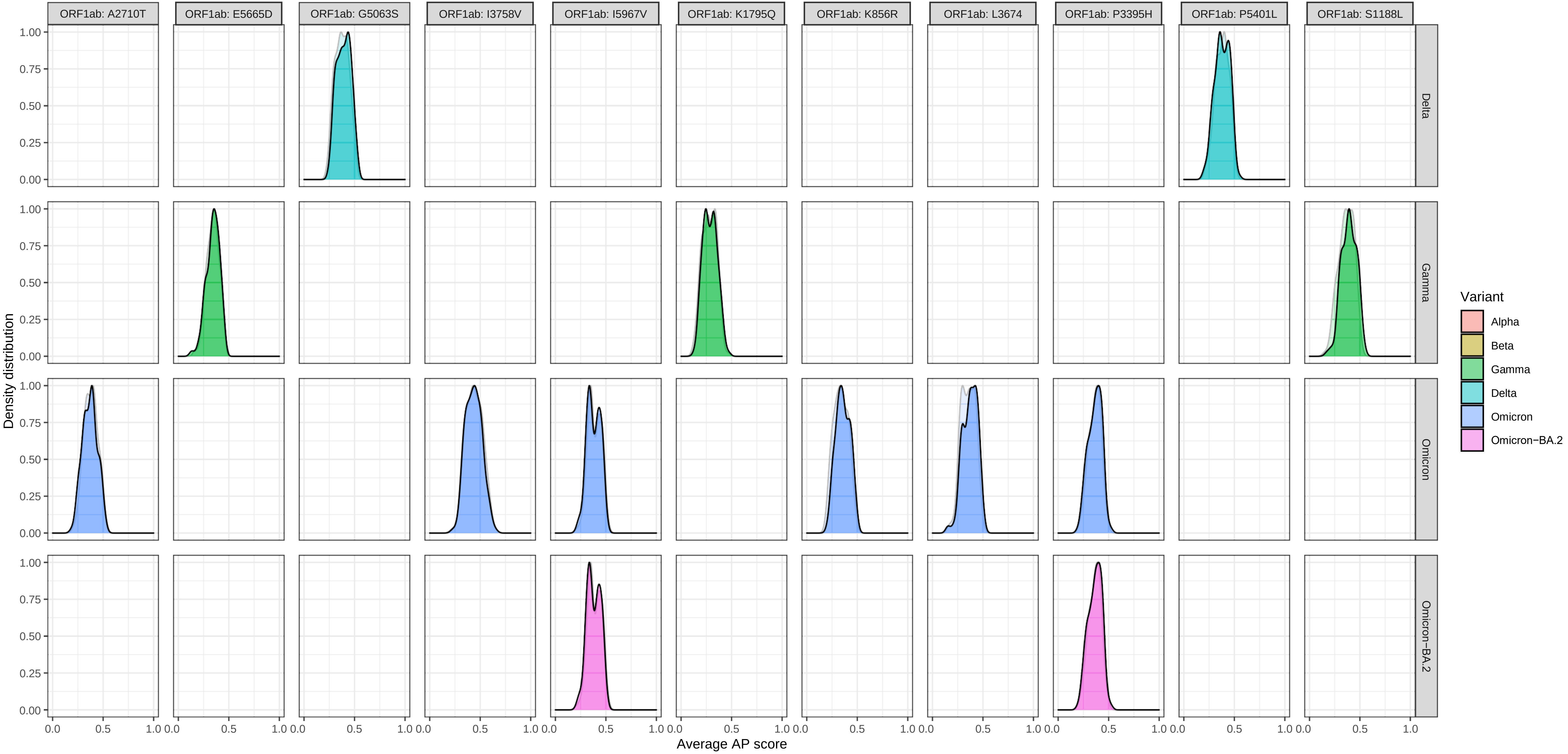

Supplementary Figure S6: Density of average AP scores at each mutation for a mutant (foreground) and wildtype (background) − protein

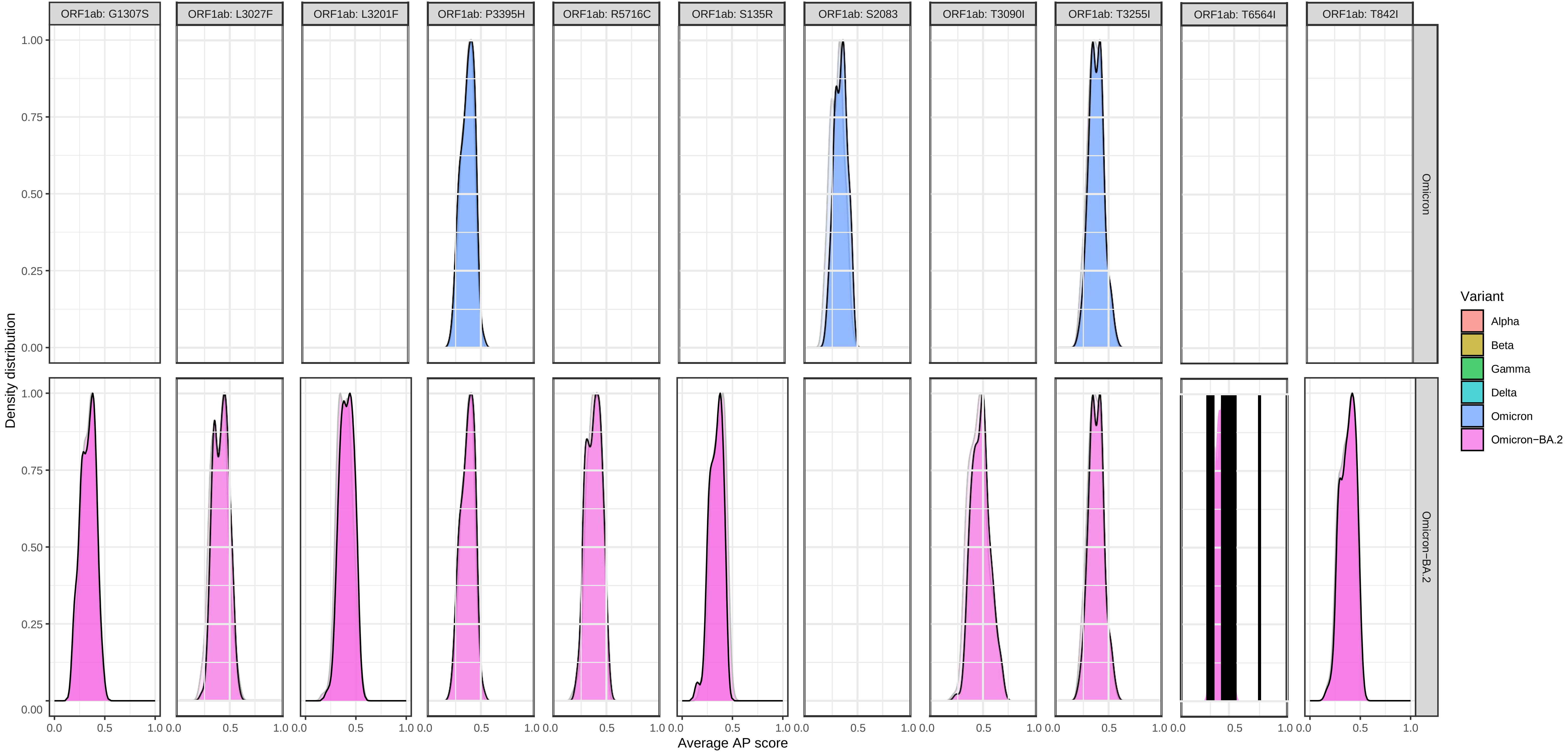

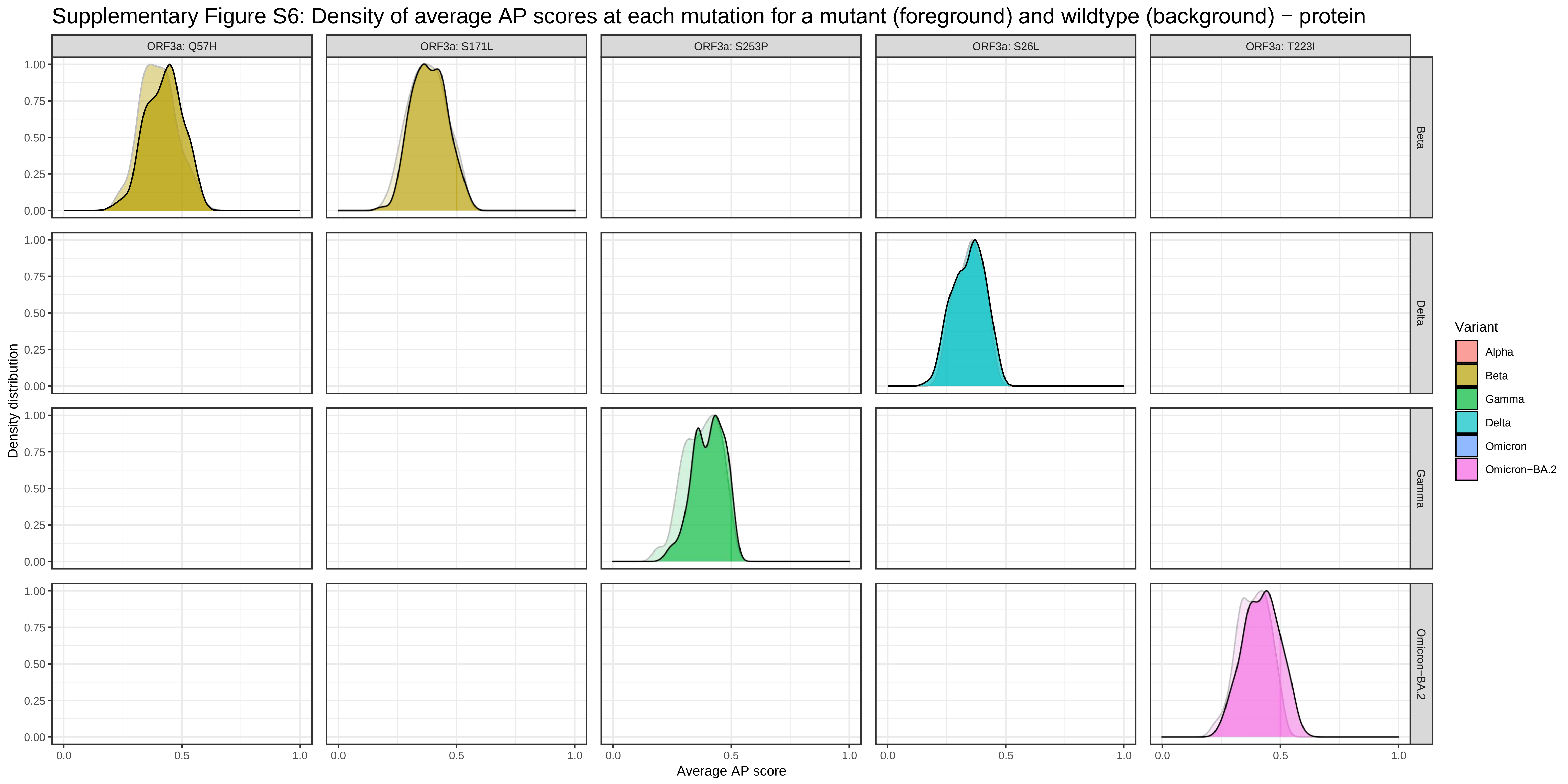

Supplementary Figure S6: Density of average AP scores at each mutation for a mutant (foreground) and wildtype (background) − protein
ORF3a: Q57H
ORF3a: S171L
ORF3a: S253P
ORF3a: S26L
ORF3a: T223I
Beta
Delta
Gamma
Omicron−BA.2
Density distribution
Average AP score
Variant
Alpha
Beta
Gamma
Delta
Omicron
Omicron−BA.2

Supplementary Figure S6: Density of average AP scores at each mutation for a mutant (foreground) and wildtype (background) − protein

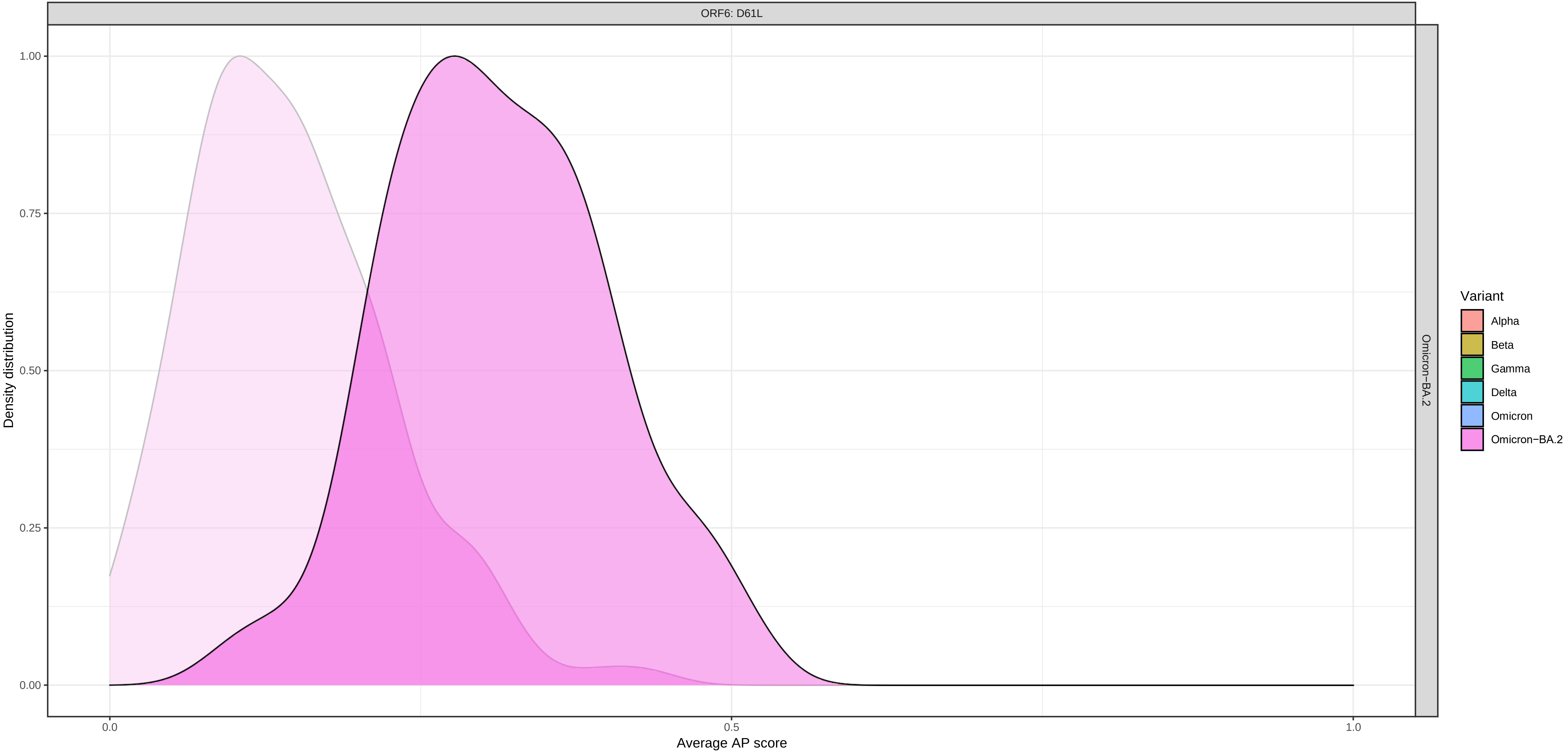

Supplementary Figure S6: Density of average AP scores at each mutation for a mutant (foreground) and wildtype (background) − protein

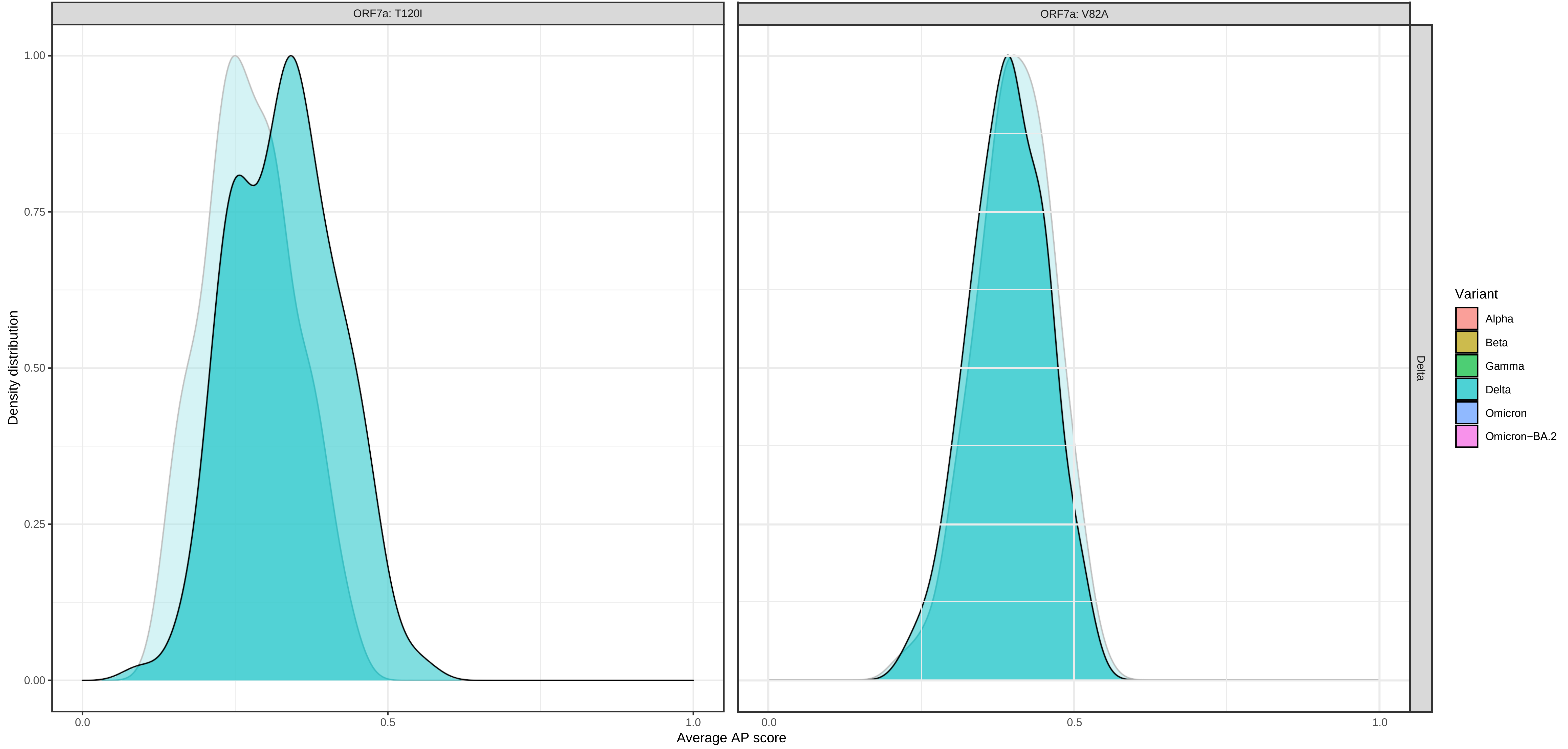

Supplementary Figure S6: Density of average AP scores at each mutation for a mutant (foreground) and wildtype (background) − protein

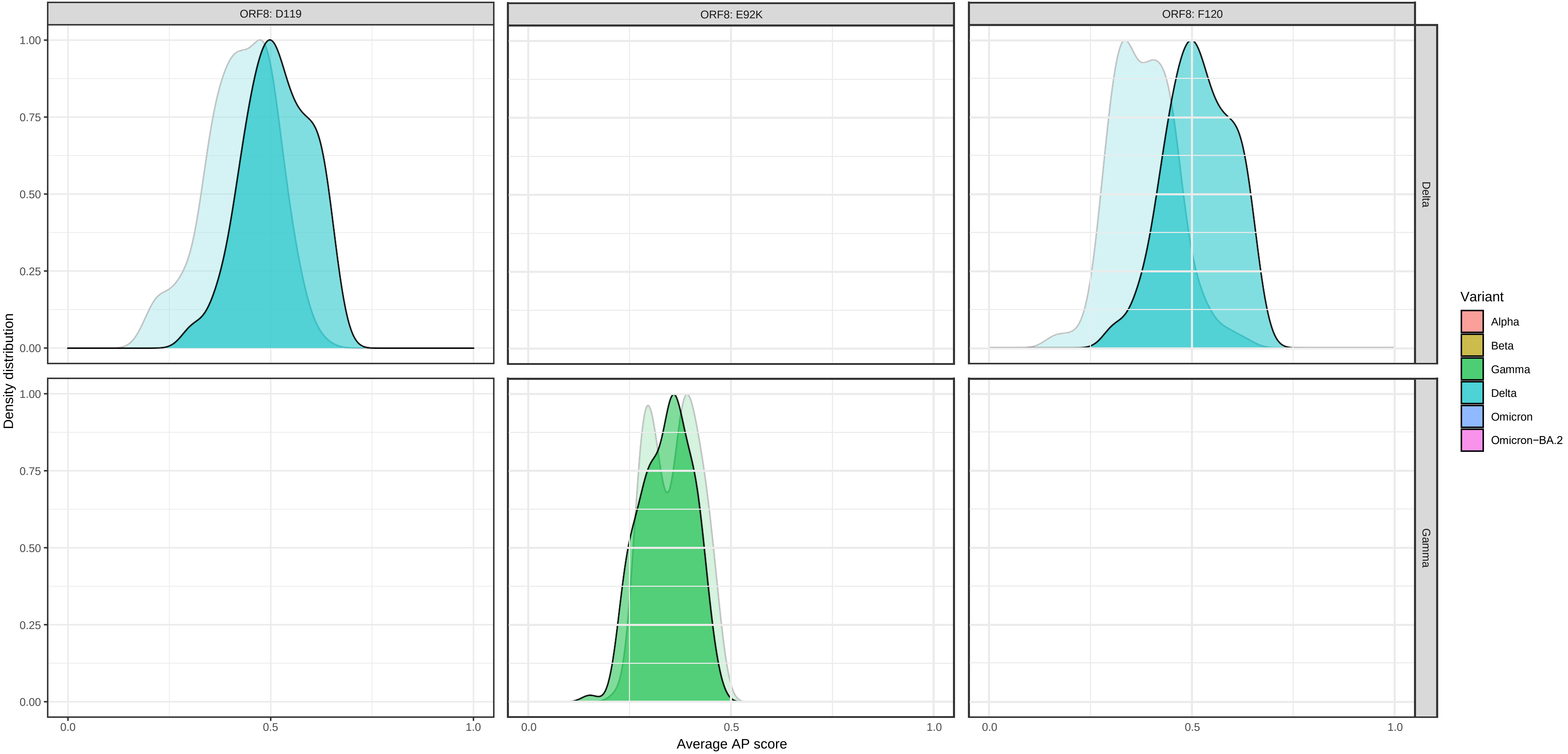

Supplementary Figure S6: Density of average AP scores at each mutation for a mutant (foreground) and wildtype (background) − protein

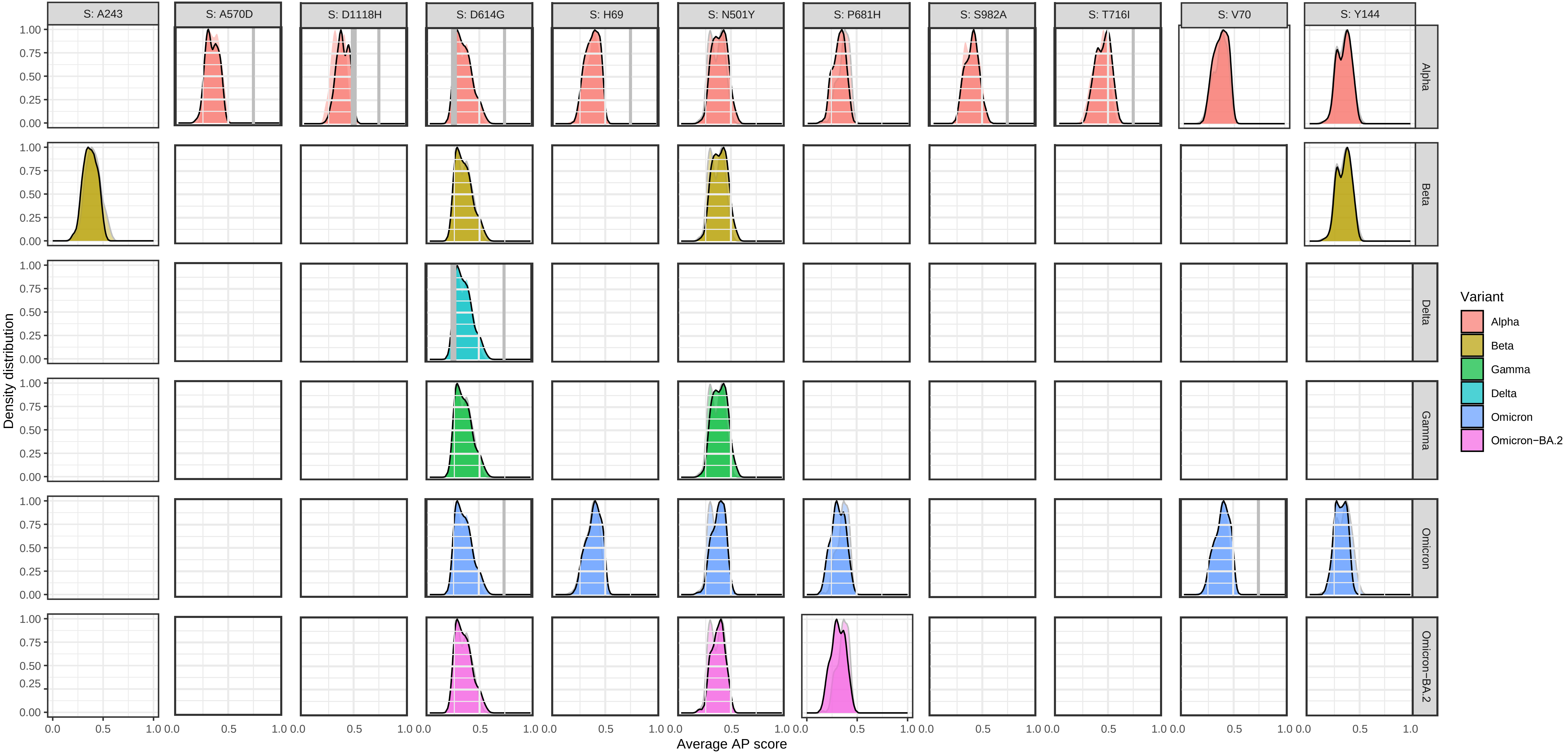

Supplementary Figure S6: Density of average AP scores at each mutation for a mutant (foreground) and wildtype (background) − protein

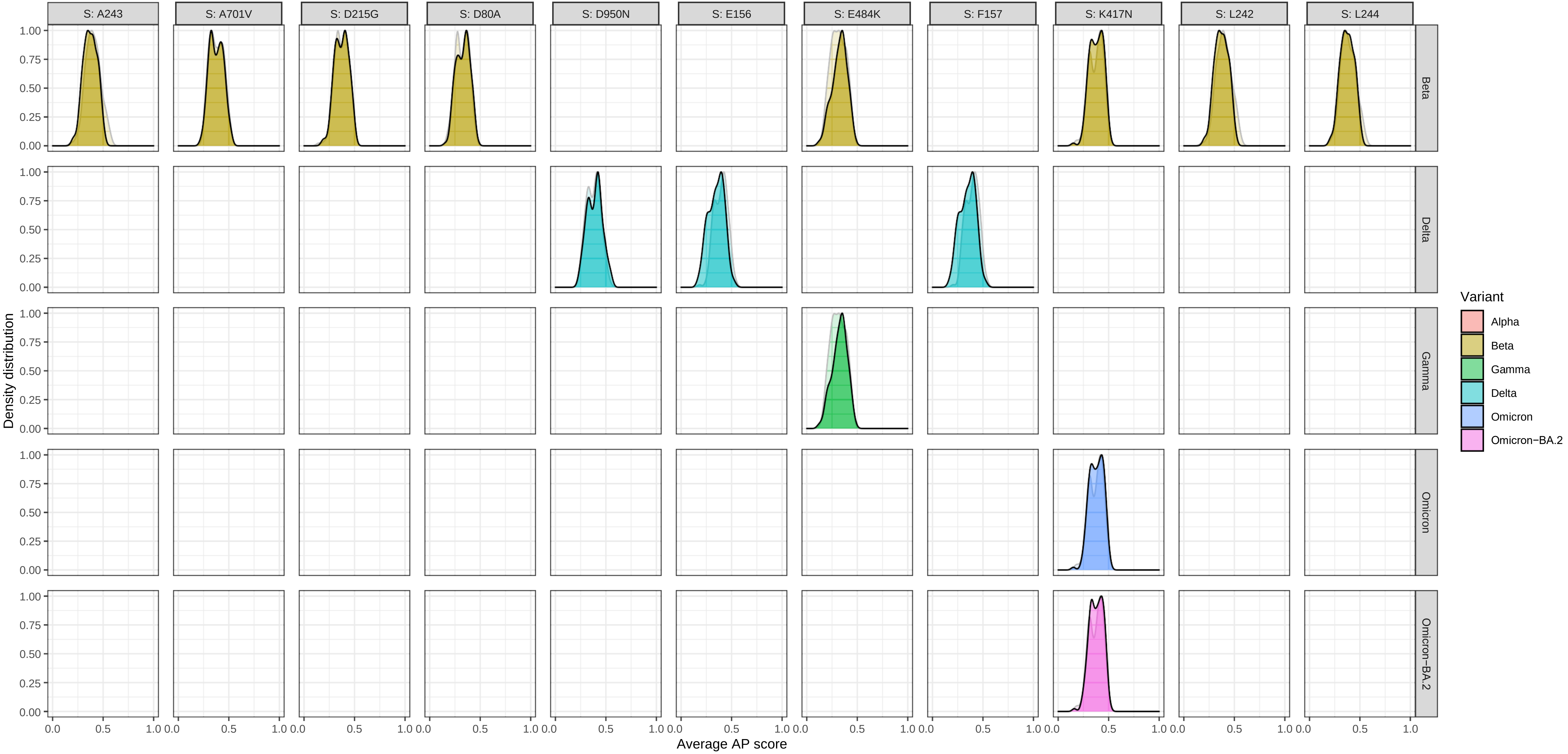

Supplementary Figure S6: Density of average AP scores at each mutation for a mutant (foreground) and wildtype (background) − protein

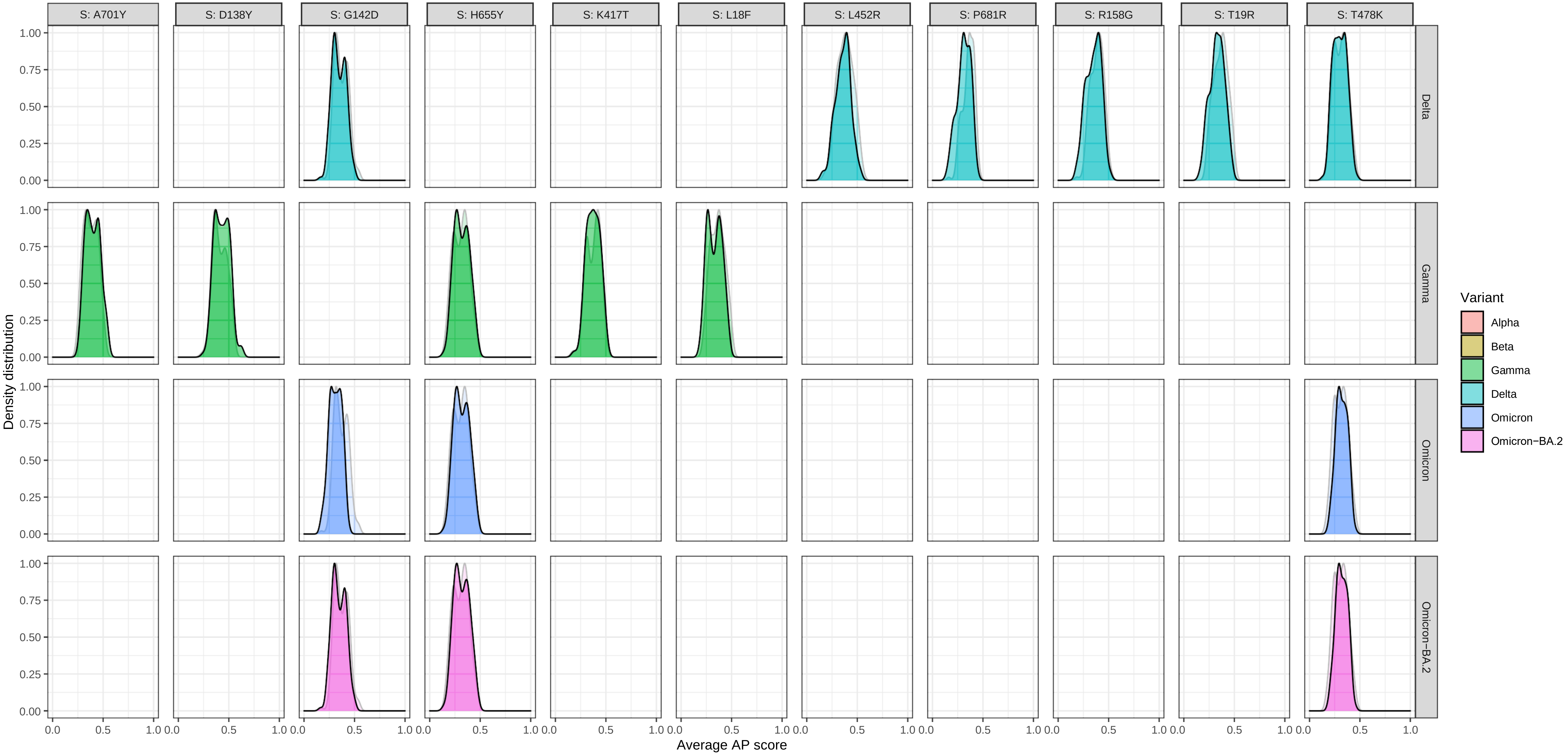

Supplementary Figure S6: Density of average AP scores at each mutation for a mutant (foreground) and wildtype (background) − protein

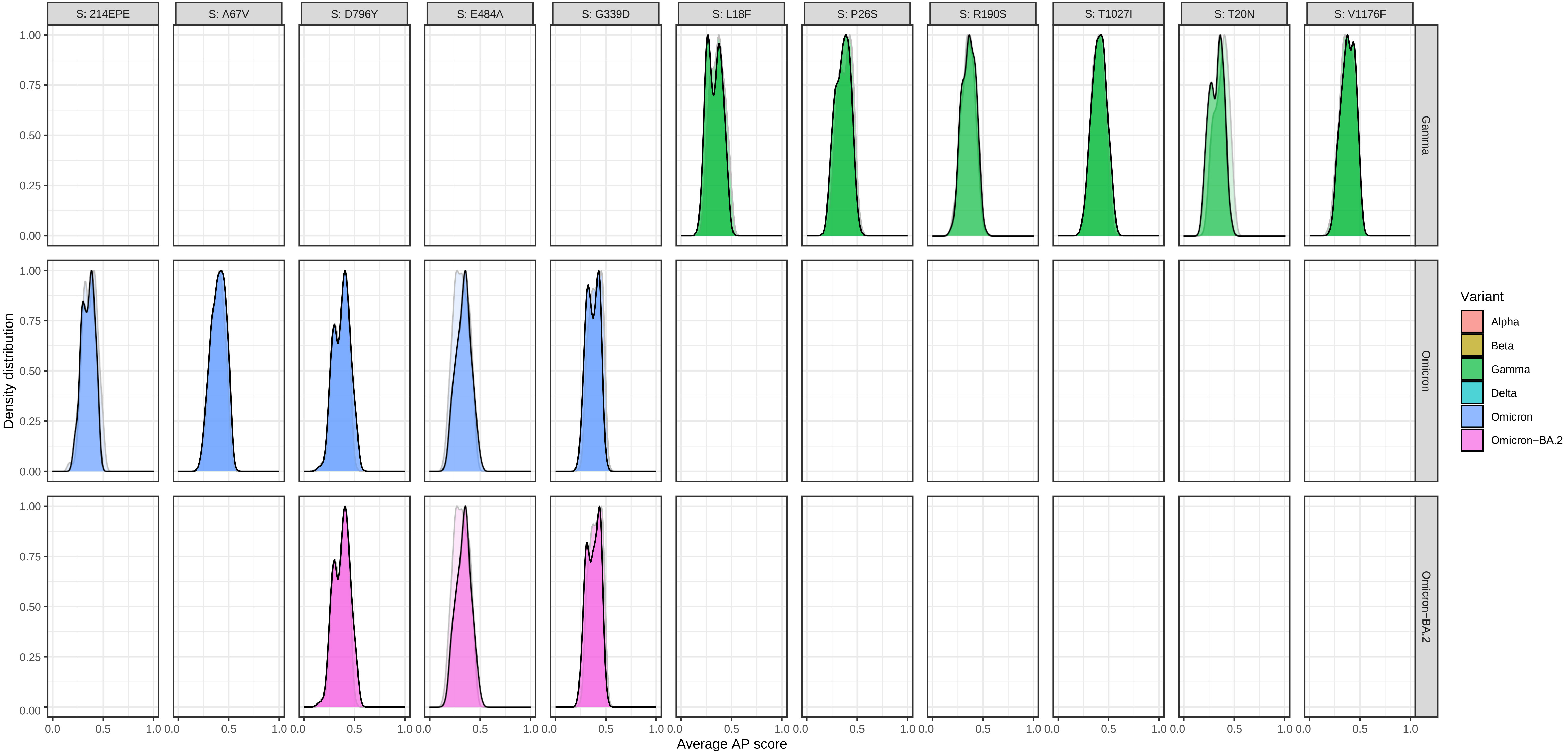

Supplementary Figure S6: Density of average AP scores at each mutation for a mutant (foreground) and wildtype (background) − protein

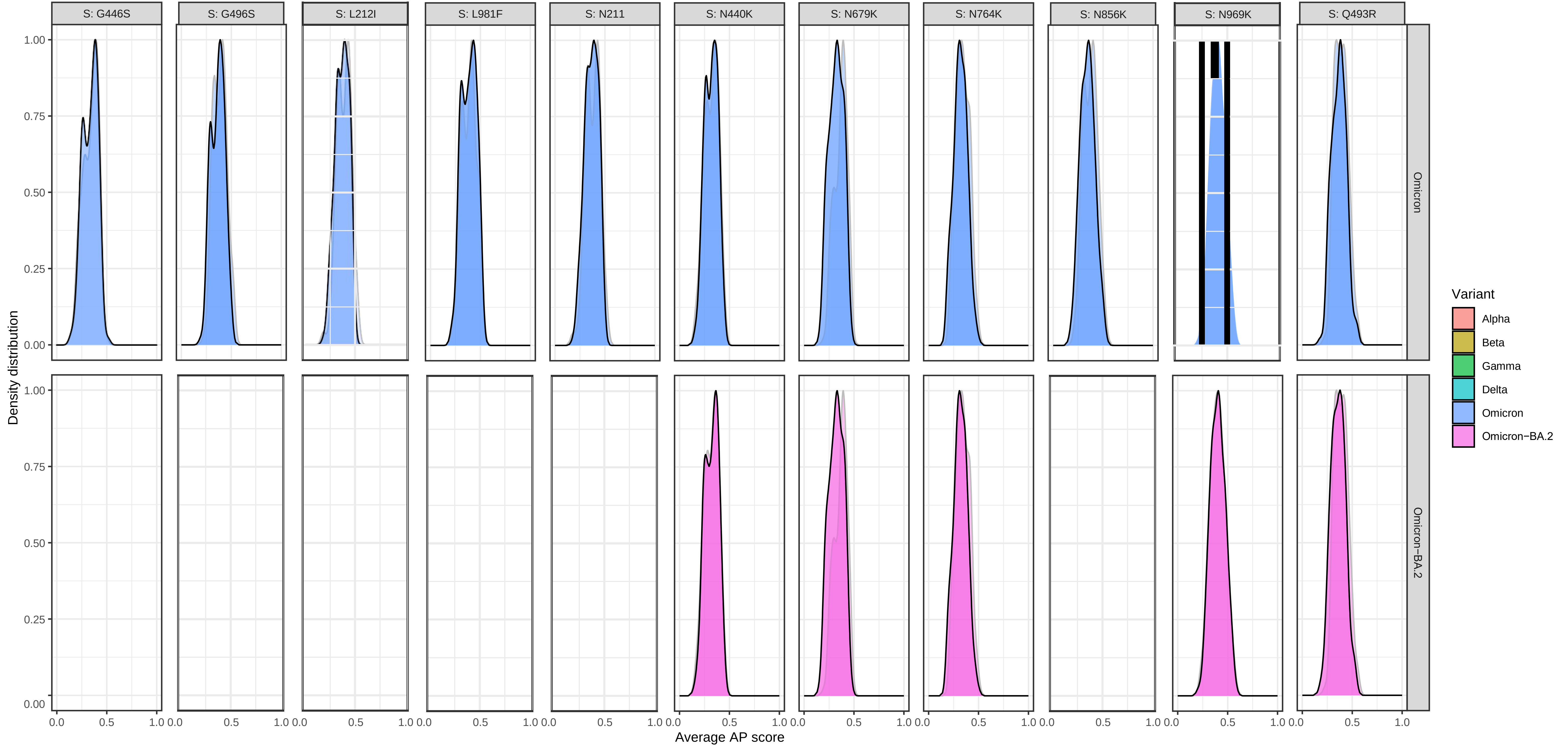

Supplementary Figure S6: Density of average AP scores at each mutation for a mutant (foreground) and wildtype (background) − protein

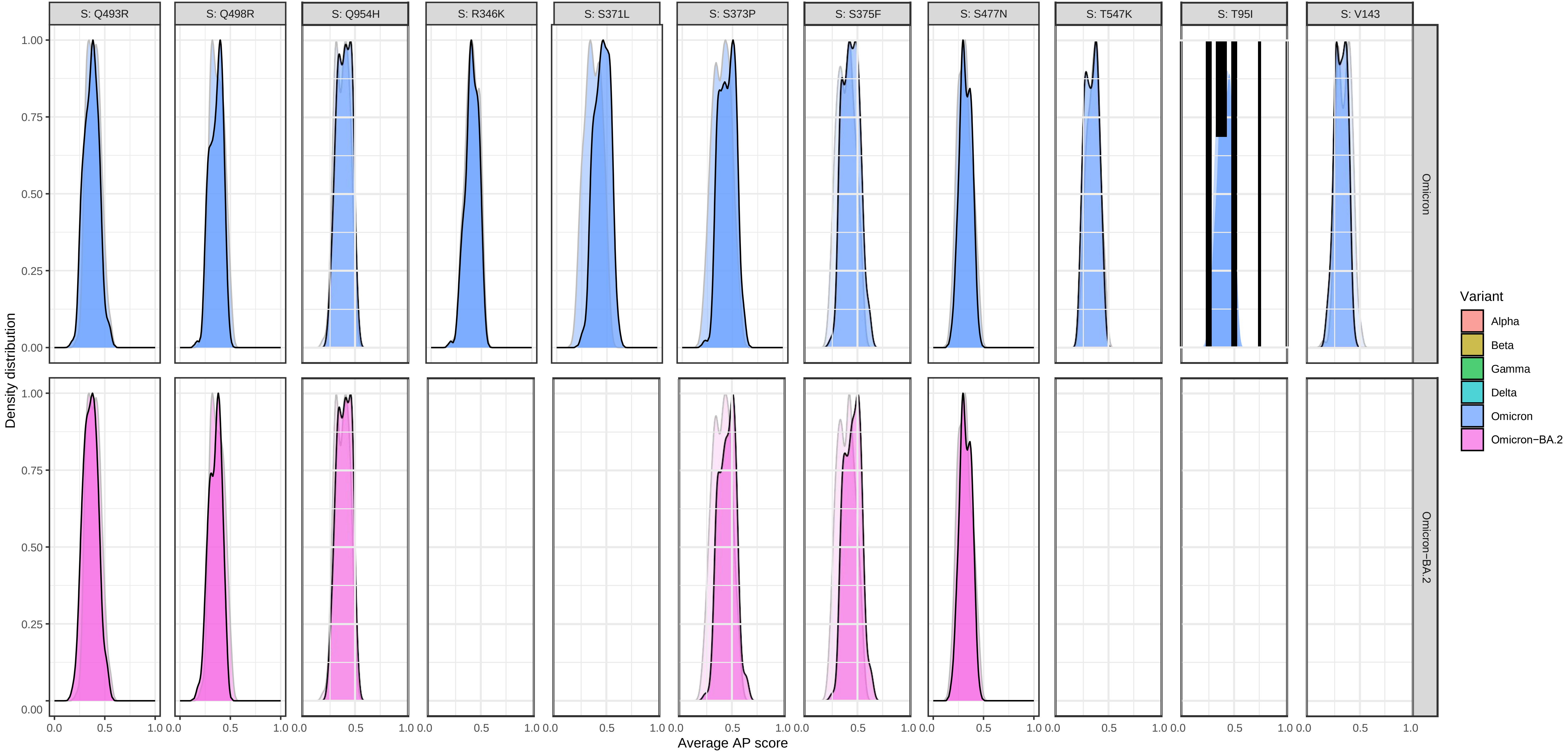

Supplementary Figure S6: Density of average AP scores at each mutation for a mutant (foreground) and wildtype (background) − protein

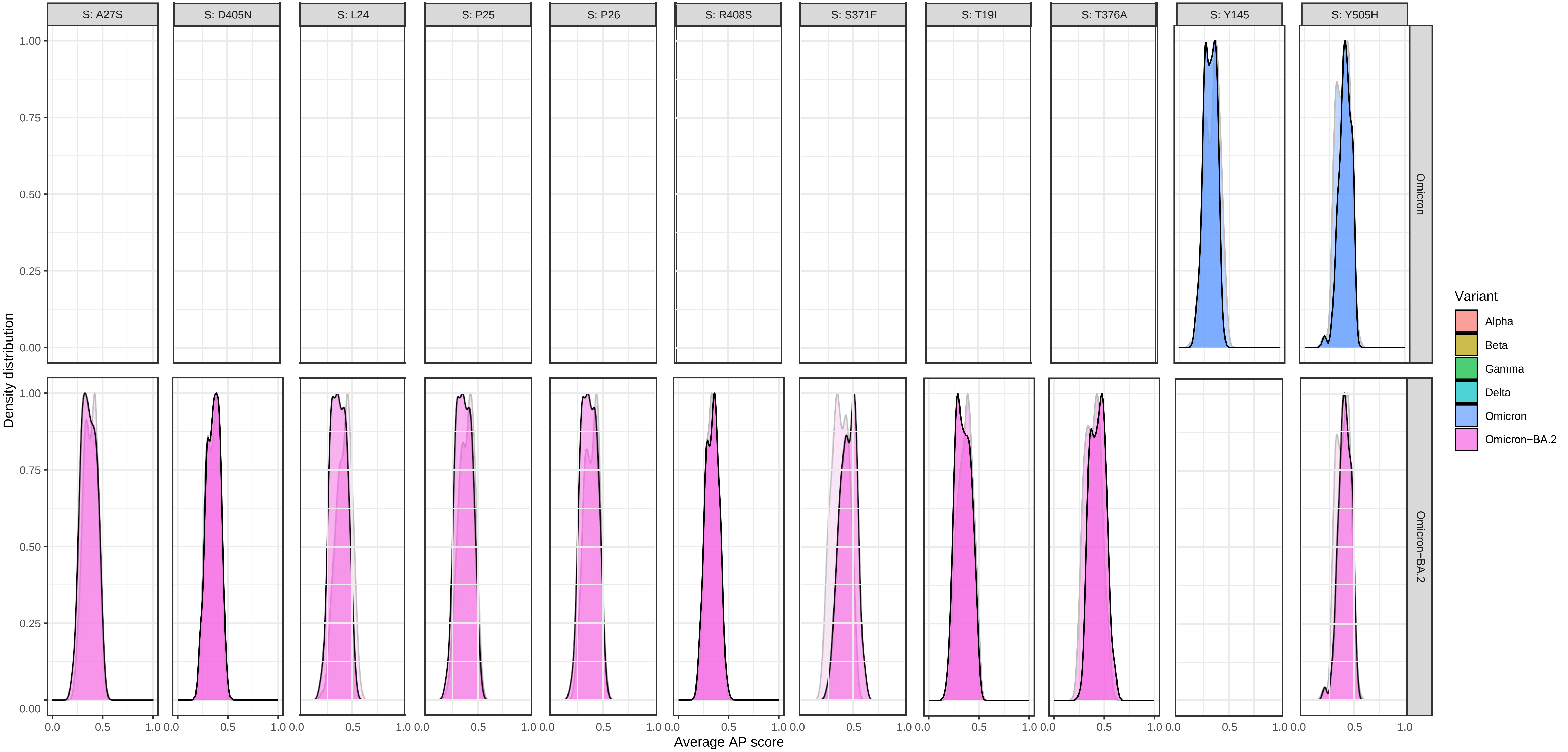

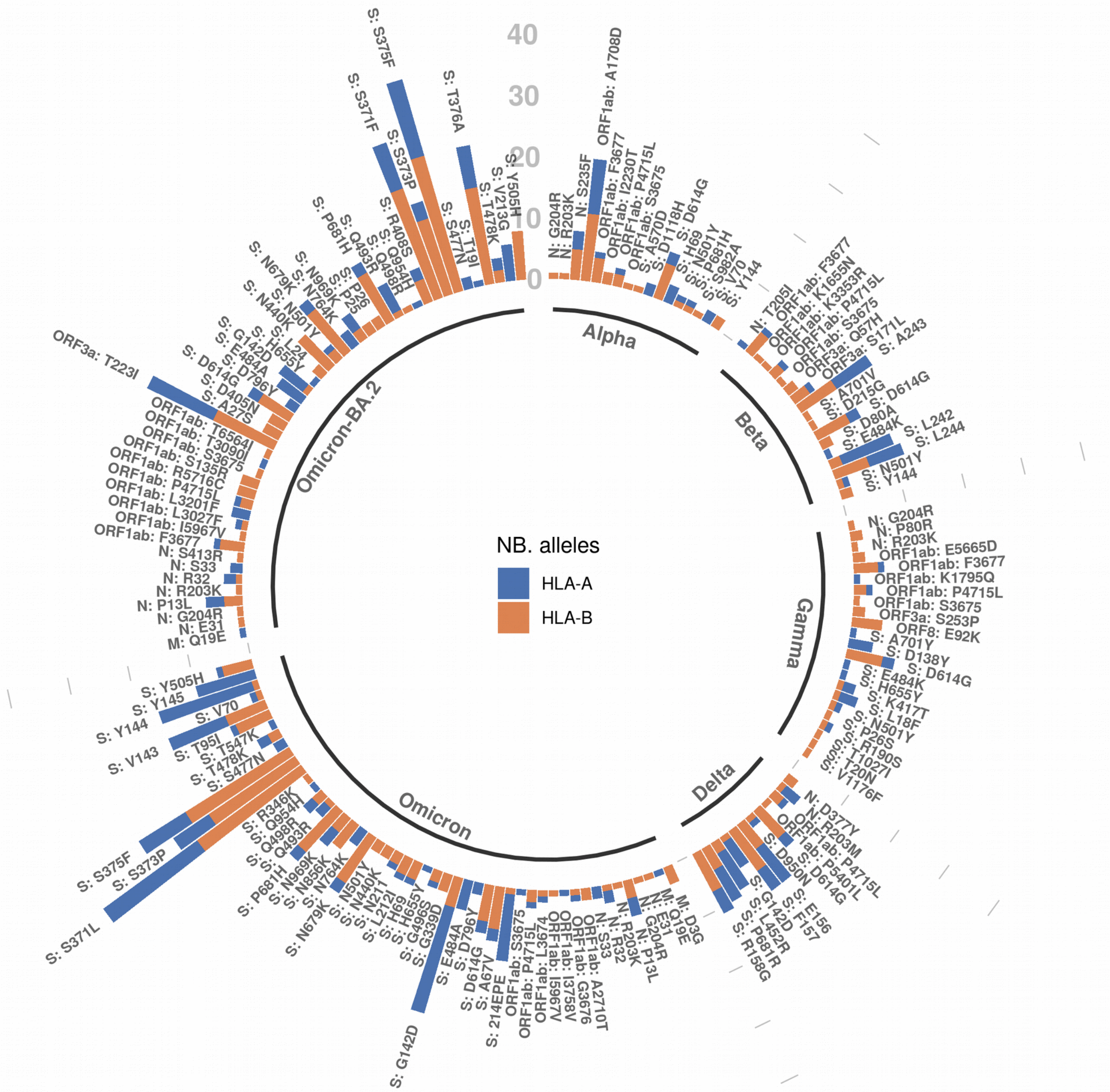

Supplementary Figure S7: This circular plot shows the number of HLA alleles per mutation for which there was a significant difference (p-value < 0.05) from the two-sided Kolmogorov-Smirnov (KS) test applied on the the variant and the wildtype AP score distribution. Only entries with an AP score above 0.5 in either the mutant or wildtype were considered for this plot. The label of each bar contains the protein name and the mutation details separated by colon. Each variant has its own group and the number of HLA-A alleles and HLA-B alleles is displayed separately for each mutation. The plot also includes the newly emerged Omicron-BA2 variant.

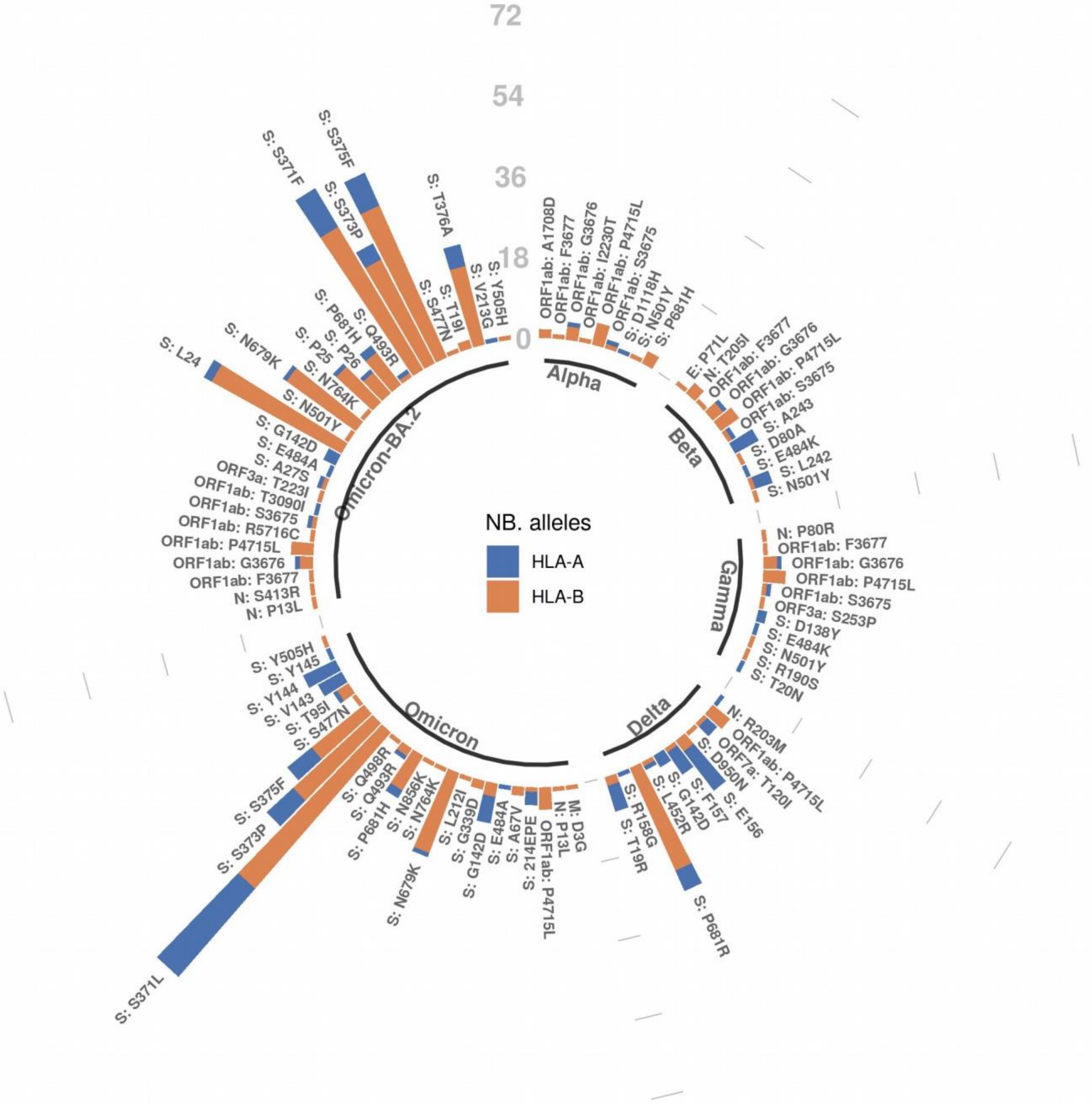

Supplementary Figure S8: This circular plot shows the number of HLA alleles per mutation for which there was a significant difference (p-value < 0.05) from the two-sided Kolmogorov-Smirnov (KS) test applied on the the variant and the wildtype AP score distribution. All AP scores (i.e., no prior filtering) were considered for this plot. The label of each bar contains the protein name and the mutation details separated by colon. Each variant has its own group and the number of HLA-A alleles and HLA-B alleles is displayed separately for each mutation. The plot also includes the newly emerged Omicron-BA2 variant.

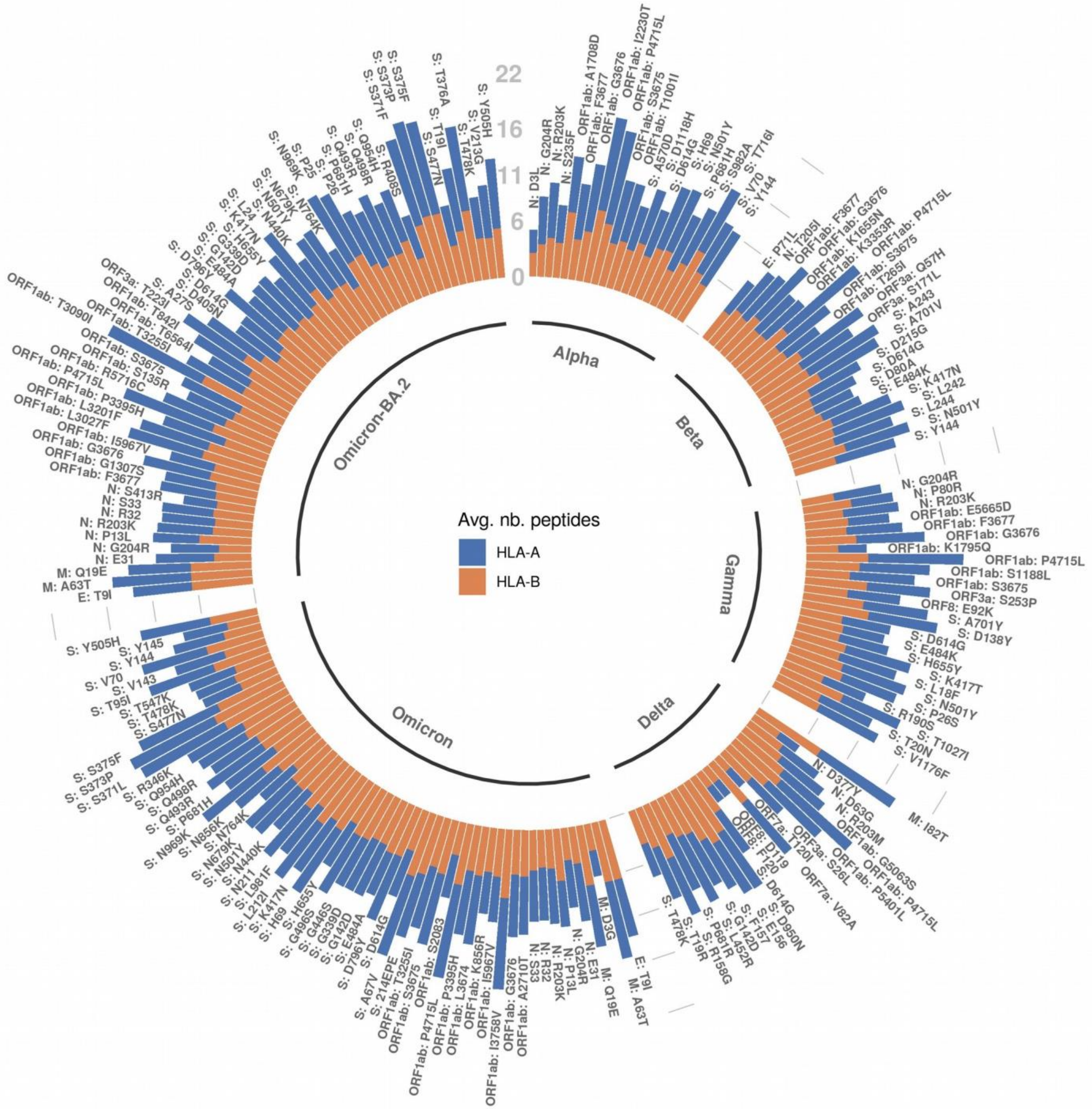

Supplementary Figure S9: This circular plot shows the average number of peptides across all HLA alleles for each of the mutations occurring in each variant. Only entries with an AP score above 0.5 in either the mutant or wildtype were considered for this plot. The label of each bar contains the protein name and the mutation details separated by colon. Each variant has its own group and the average across HLA-A alleles and HLA-B alleles is displayed separately for each mutation. This plot can give an indication about the resolution used for the KS two sided significance test. The plot also includes the newly emerged Omicron-BA2 variant.

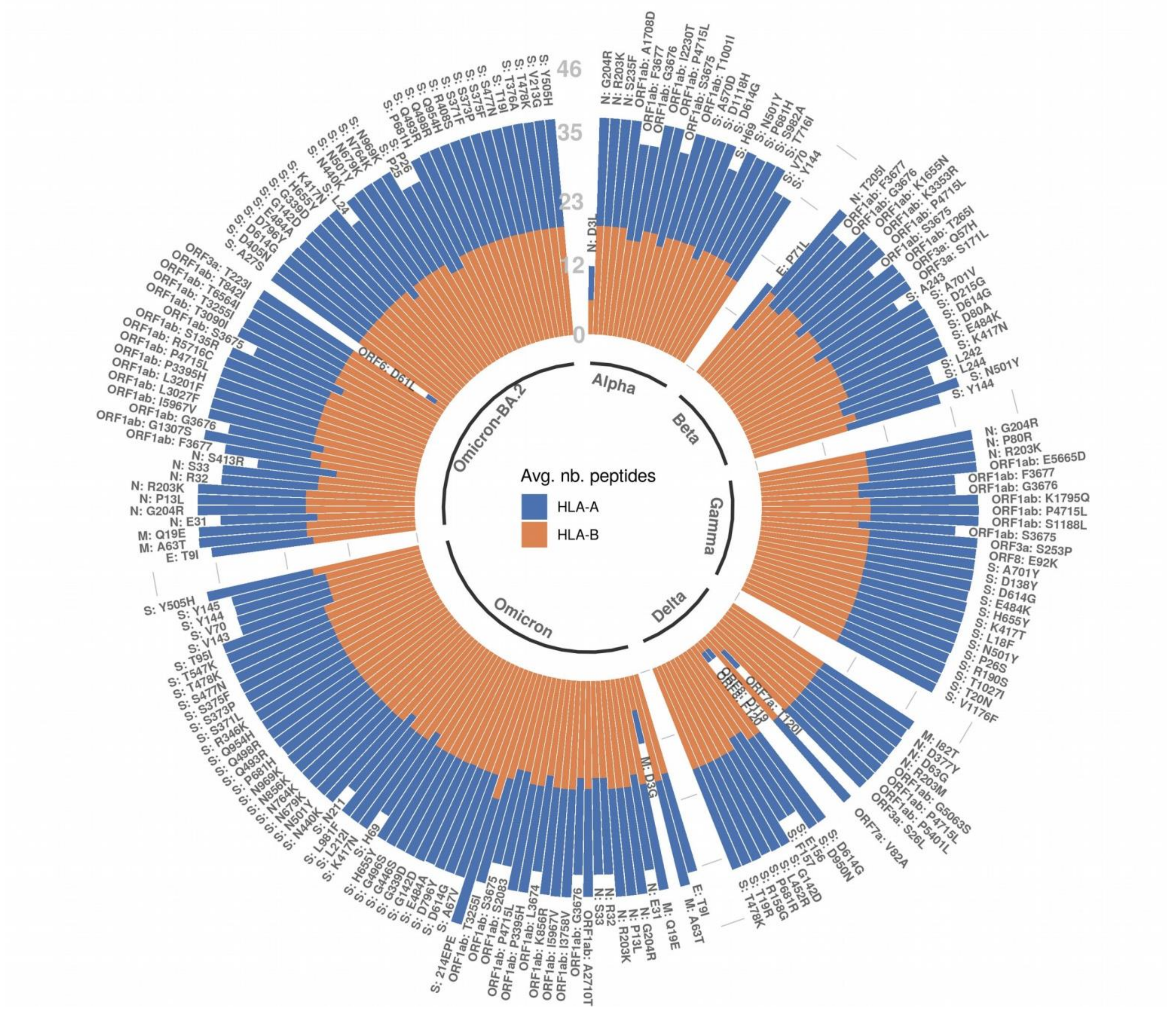

Supplementary Figure S10: This circular plot shows the average number of peptides across all HLA alleles for each of the mutations occurring in each variant. All AP scores (i.e., no prior filtering) were considered for this plot. The label of each bar contains the protein name and the mutation details separated by colon. Each variant has its own group and the average across HLA-A alleles and HLA-B alleles is displayed separately for each mutation. This plot can give an indication about the resolution used for the KS two sided significance test. The plot also includes the newly emerged Omicron-BA2 variant.
